# Early Versus Late Tracheostomy: An Umbrella Review and Meta-Analysis

**DOI:** 10.3390/jcm15176685

**Published:** 2026-08-28

**Authors:** Sven Mostberger, Marija Glisic, Magda R. Gamba, Claudio Perret, Gabi Mueller

**Affiliations:** 1 Faculty of Health Sciences and Medicine, University of Lucerne, 6002 Lucerne, Switzerland; magda.gamba@zhbluzern.ch (M.R.G.); claudio.perret@paraplegie.ch (C.P.); gabi.mueller@paraplegie.ch (G.M.); 2 Swiss Paraplegic Research, 6207 Nottwil, Switzerland; marija.glisic@paraplegie.ch; 3 Institute of Social and Preventive Medicine (ISPM), University of Bern, 3012 Bern, Switzerland; 4 Central and University Library of Lucerne, 6002 Lucerne, Switzerland

**Keywords:** meta-analysis, tracheostomy, pneumonia, mechanical ventilation, length of stay, mortality

## Abstract

**Background:** Optimal tracheostomy timing remains controversial due to conflicting evidence across published systematic reviews. To clarify this ambiguity within specific patient populations (critically ill, traumatic brain injury, stroke, trauma, COVID-19, and spinal cord injury), an umbrella review of systematic reviews was conducted comparing early versus late tracheostomy or prolonged intubation. Additionally, potential benefits in clinical outcomes were evaluated across a mixed-etiology cohort of mechanically ventilated patients. **Methods:** MEDLINE, Embase, Cochrane Library, and Web of Science were searched from inception through July 2024. Systematic reviews evaluating the impact of tracheostomy timing on mechanical ventilation duration, ventilator-associated pneumonia risk, ICU/hospital length of stay, and mortality were included. Two authors independently extracted data using a standardized form. Methodological quality was assessed via AMSTAR 2 and certainty of evidence via GRADE. Random-effects meta-analyses were conducted for each outcome, stratified by study design (RCTs vs. non-RCTs), with subgroup analyses exploring patient subpopulations. **Results:** Evidence was synthesized from 9 systematic reviews (24 unique RCTs, 54 non-RCTs). In RCTs, moderate certainty evidence suggests that early tracheostomy reduces ventilator-associated pneumonia (OR 0.66, 95% CI 0.46 to 0.94, *p* = 0.02) and mechanical ventilation duration (MD −3.76 days, 95% CI −6.01 to −1.52, *p* < 0.001) compared to late tracheostomy or prolonged intubation. Early tracheostomy may reduce ICU length of stay (MD −6.64 days, 95% CI −9.92 to −3.35, *p* < 0.001, low certainty). The effect of early tracheostomy on hospital length of stay and mortality remains uncertain. **Conclusions:** Early tracheostomy shows potential to reduce ventilator-associated pneumonia and mechanical ventilation duration in mixed-etiology cohorts. It may also be associated with shorter ICU length of stay, while its effect on mortality and hospital stay remains uncertain. Future research should establish standardized definitions of tracheostomy timing and prioritize high-quality, etiology-specific RCTs in underrepresented cohorts (e.g., traumatic brain injury, stroke, spinal cord injury).

## 1. Introduction

Optimal tracheostomy timing remains uncertain, despite 10–25% of intensive care unit (ICU) patients undergoing the procedure [1,2]. The most common indication for tracheostomy is acute respiratory failure with expected need for prolonged mechanical ventilation [3]. Tracheostomy offers advantages over prolonged translaryngeal intubation including reduced airway resistance, fewer laryngeal lesions, enabling communication and oral nutrition, increased patient comfort, better pulmonary secretion clearance, and easier nursing care [4]. Tracheostomy risks include pneumonia, pneumothorax, bleeding, wound infection, tracheal stenosis, and death [5]. Deciding when to perform a tracheostomy is challenging, as it involves the difficulty in predicting the need for a prolonged mechanical ventilation and the consequential risks of premature or delayed procedures [6].

Tracheostomy timing impacts outcomes in mechanically ventilated patients. Tracheostomy was performed after 14 days of intubation for a long time, a rule that originated out of the trade-off between the complications of endotracheal intubation and tracheostomy during the 1960s and 1970s [7]. In 1989, intubation was recommended for mechanical ventilation of up to 10 days and tracheostomy for an anticipated requirement of more than 21 days. Daily patient assessments were recommended for the time in between and in cases of uncertainty. However, these recommendations were made based on expert opinion and in absence of strong evidence at the time [8].

Today, optimal tracheostomy timing remains a subject of debate, although the evidence base has grown considerably since 1989. Several meta-analyses aimed at comparing the effect of early tracheostomy (ET) with late tracheostomy (LT) reported conflicting findings. For example, one systematic review (SR) found ET compared to LT to be associated with reduced short-term mortality and ventilator-associated pneumonia (VAP), while another found no such benefits [9,10].

Beyond general ICU populations, various SRs focus on specific sub-populations, such as patients with traumatic brain injury (TBI), spinal cord injury (SCI), or trauma. This has resulted in a fragmented evidence landscape with divergent findings, complicating interpretation and application in clinical practice. Importantly, many existing SRs aggregate data from RCTs and non-RCTs [11,12,13]. This approach is prone to incorporating selection bias from the non-RCTs and overestimating the beneficial effects of ET.

Unlike previously published reviews, this umbrella review is the first to synthesize data across both general and disease-specific critically ill populations. To date, no overarching review has systematically consolidated this evidence base while rigorously isolating evidence from RCTs from non-RCTs.

The present umbrella review, with outcomes stratified by study design (evidence from RCTs vs. non-RCTs), aimed to evaluate how tracheostomy timing influences mechanical ventilation duration, VAP, ICU and/or hospital length of stay (LOS), and mortality in a mixed-etiology cohort of mechanically ventilated patients. Additionally, the review aimed to resolve the conflicting evidence regarding optimal tracheostomy timing within specific patient subpopulations.

## 2. Materials and Methods

The results of this umbrella review have been reported in accordance with previously published guidelines on umbrella reviews [14,15], and following the PRIOR statement. The completed PRISMA 2020 checklist is provided in Supplementary Table S10. The protocol was pre-registered on PROSPERO, the international prospective register of systematic reviews (CRD420250575128).

### 2.1. Data Sources and Search Strategy

MEDLINE, Embase, Cochrane Library, and Web of Science were searched from inception to 9–10 July 2024. The search strategy was developed with a medical librarian (M.R.G.) and incorporated both subject headings and free-text terms extracted from titles and abstracts of relevant articles. Validated search filters were used [16,17], and deduplication of results was performed in Endnote^®^ 20 (Clarivate, Philadelphia, PA, USA) following the Bramer Method [18]. The full search strategies are provided in Supplementary File S1.

### 2.2. Study Selection

Eligibility criteria were developed using the Population, Intervention/Exposure, Comparison, and Outcomes framework. SRs, as defined by the Cochrane Handbook [19], were included if conducted in adult humans and comparing ET to LT or prolonged intubation on at least one of the following outcomes: VAP, mechanical ventilation duration, ICU or hospital LOS, and mortality. Definitions of tracheostomy timing provided within the included reviews were adopted. SRs focused solely on pediatric populations (defined as individuals under 18 years of age) or solely based on case series or case studies were excluded. Titles, abstracts, and full texts were screened by two independent reviewers (S.M., G.M., M.G. or C.P.) via Covidence^®^ systematic review software (Veritas Health Innovation, Melbourne, Australia; www.covidence.org). Discrepancies were resolved by consensus or a third reviewer. To identify overlap, first author and publication year of included primary studies from eligible SRs were extracted into a citation matrix and compared. In case of overlapping or partially overlapping included SRs, the most up-to-date SR for each specific population was used for synthesis. Where multiple SRs were published in the same year, the most comprehensive (defined by the number of primary studies included and relevant outcomes) was chosen for inclusion. Methodological quality of systematic reviews was evaluated post-inclusion and was not an eligibility criterion for review selection. The detailed, population-specific selection process and rationale for all included and excluded systematic reviews are mapped in Supplementary Table S1.

### 2.3. Data Extraction

Two authors (S.M. and G.M.) independently extracted data using a standardized extraction form. Dissents were resolved by discussion or a third author. In case of missing or ambiguous data across SRs, original primary studies were reviewed. A citation matrix with the nine SRs used for synthesis was created to identify primary study overlaps at the comparison and outcome level. From that, overlap was quantified using the Corrected Covered Area (CCA) [20].

Extracted data included the following: lead author, year of publication, design and number of included studies, participants characteristics, definitions of ET and LT, risk of bias assessment and reported summary estimates with their corresponding 95% confidence interval (CI) including heterogeneity measures, publication bias assessment, and subgroup analyses.

### 2.4. Quality Assessment

Methodological quality of SRs eligible for inclusion was assessed independently by two reviewers (S.M. and G.M.) using the AMSTAR 2 tool [21]. Primary study risk of bias assessments were extracted and stratified by outcome and design. Conflicting assessments across SRs were resolved by adopting the most conservative rating. Missing assessments were independently re-evaluated in duplicate by two reviewers using the revised Cochrane risk of bias tool for RCTs or the Newcastle-Ottawa Scale for non-randomized studies [22,23]. Any differences in the assessment results were resolved by consensus or consulting a third reviewer.

### 2.5. Data Synthesis and Analysis

To avoid inflated precision from overlapping primary studies across SRs, deduplicated de novo meta-analyses were performed for each outcome in the main pooled models. For population-specific subgroup analyses, individual primary studies were included in their respective target subgroups (e.g., the same study could contribute to the “critically ill” and “COVID-19” groups), but remained non-overlapping within any single subgroup model. Evidence was stratified by study design (RCTs vs. non-RCTs) to avoid potential bias and to account for methodological differences. Meta-analyses were conducted using the meta esize command in Stata (version 19.5, StataCorp LLC, College Station, TX, USA). For binary outcomes (mortality, VAP), 2 × 2 contingency table counts were extracted. For continuous outcomes (mechanical ventilation duration, ICU, and hospital LOS), group sample sizes, means, and standard deviations were extracted. Where continuous data were reported as medians and interquartile ranges, means and standard deviations were estimated using the methods of Wan et al. [24]. When extracted values differed across systematic reviews, original primary studies were consulted for verification. For mortality, where different systematic reviews reported varying follow-up durations for the same primary study, the longest available follow-up time point was prioritized for meta-analytic pooling. For zero-event trials in binary analyses, a standard continuity correction of 0.5 was applied. No multi-arm trials were present among the included studies. Complete study-level extraction datasets for the primary pooled models and subgroup analyses are provided in Supplementary Data S1 and Supplementary Data S2, respectively. All meta-analyses were performed using random-effects models with the DerSimonian-Laird estimator to account for the anticipated between-study heterogeneity and maintain methodological consistency with the source reviews. As a sensitivity analysis to assess model robustness, restricted maximum likelihood (REML) random-effects models with Knapp– Hartung standard error adjustments were also fitted. The main pooled models comprised all primary studies regardless of their definition of tracheostomy timing. To account for clinical heterogeneity from variable definitions of tracheostomy timing across primary studies, a sensitivity analysis was performed restricting inclusion to primary studies comparing ET (≤7 days) versus LT (>7 days) or no tracheostomy. Outcome data from the performed meta-analyses were summarized in structured tables. For each outcome, pooled effect estimates are reported with 95% CIs, *p*-values, heterogeneity metrics, publication bias assessments and Grading of Recommendations, Assessment, Development, and Evaluation (GRADE) ratings. Results are presented for the total study population and stratified by diagnosis subgroups.

### 2.6. Reporting Bias and Certainty Assessment

If available, measures of reporting bias were extracted from the included SRs. For the de novo meta-analyses comprising 10 or more primary studies, publication bias was assessed using funnel plots and Egger’s test [19]. The GRADE [25] approach was used to evaluate the certainty of evidence for each outcome across five domains, as follows: risk of bias, inconsistency, indirectness, imprecision, and publication bias. Evidence was graded as high, moderate, low, or very low. Certainty of evidence was graded as high, moderate, low, or very low.

### 2.7. Role of the Funding Source

The funding source for the study had no role in design, conduct, and reporting of this review.

## 3. Results

**Figure 1:**
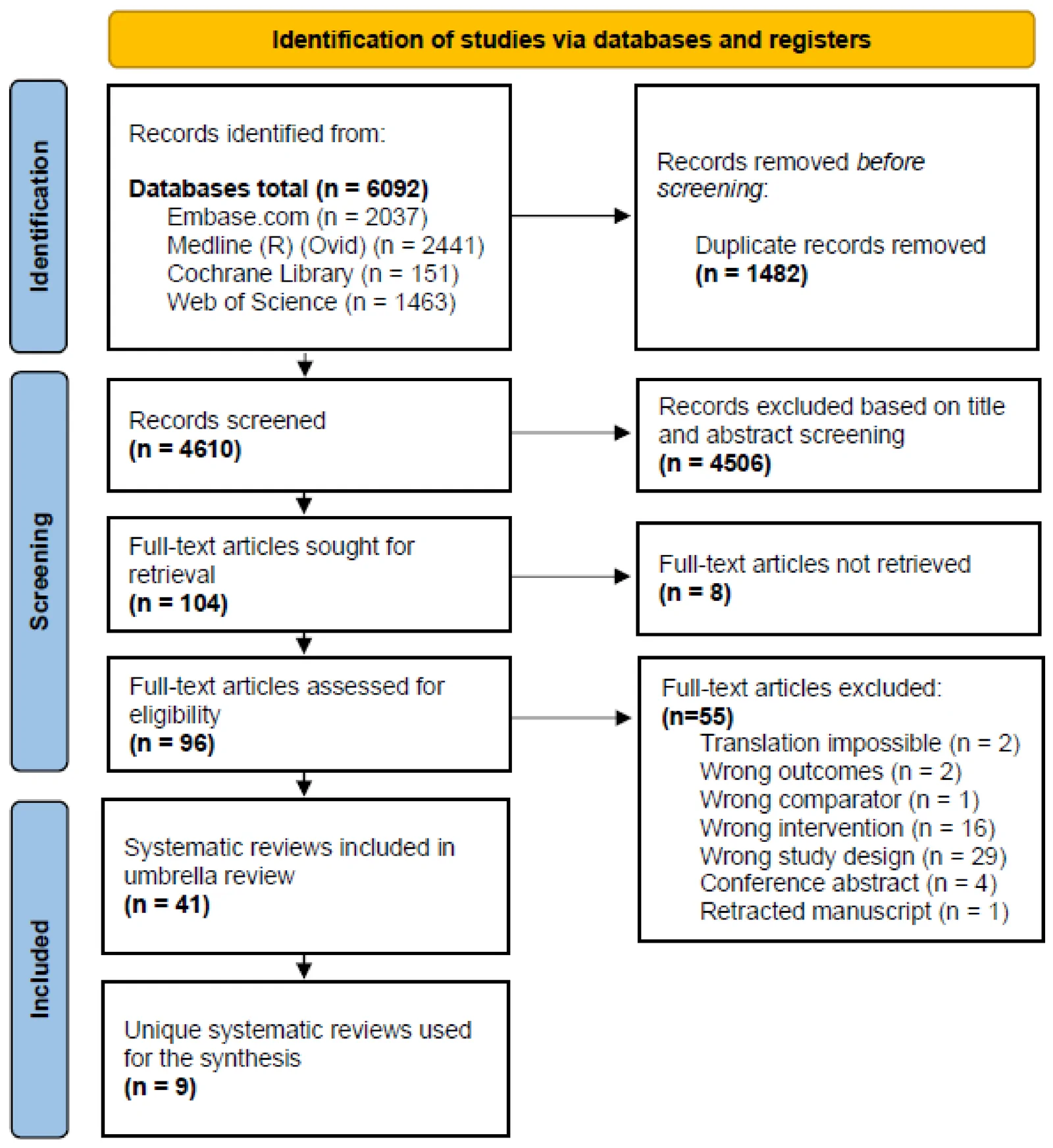
PRISMA 2020 flow diagram of studies included in current review. From [26].

A total of 4610 records were identified. Title and abstract screening yielded 96 full-text articles which were assessed for eligibility. Of these, 55 articles were excluded at this stage (Supplementary Table S2). This umbrella review included 41 articles in total, of which 9 unique SRs representing 78 unique original studies (24 RCTs; 54 non-RCTs) were used for synthesis. The complete study identification, screening, and selection process is illustrated in Figure 1. The specific SR selection process and rationale for synthesis are summarized in Supplementary Table S1.

### 3.1. Study Characteristics

The 41 reviews included were published between 1998 and 2023, with 53.7% being published after 2019. The majority (58.5%) applied no time restrictions for the search strategy and searched for more than three electronic databases. A meta-analysis was provided in 92.7% of the SRs included. The primary focus was on critically ill populations (46.3%), COVID-19 (14.6%), and traumatic brain injury (12.2%).

Among the nine SRs used for synthesis, substantial heterogeneity was observed in the definitions of tracheostomy timing. Three reviews [9,11,13] defined ET as performed within the first 7 days after intubation/initiation of mechanical ventilation, with LT defined as any time thereafter. One defined ET as up to and including day 10 [27], while the other defined it as occurring before day 10 [28]. Three reviews did not provide a single uniform review-level definition, but the upper cut-offs for ET among included primary studies were <10 days [29], ≤10 days [30], and ≤14 days [12]. One categorized tracheostomy timing into three groups (≤4 days, 5–12 days, and ≥13 days) without specifying which intervals constituted early or late [31]. Detailed characteristics of all 41 included articles are presented in Supplementary Table S3, whereas Table 1 focuses specifically on the 9 systematic reviews selected for synthesis.

### 3.2. Primary Study Overlap

Initial study overlap was moderate (CCA = 6%) across the included SRs for synthesis. RCT overlap was very high (CCA 21%), while non-RCT overlap was slight (CCA 1%). On the outcome level of RCTs, overlap was moderate for VAP, mechanical ventilation duration, ICU LOS, and mortality (CCA range: 8–10%), while there was no overlap for hospital LOS. Overlap of non-RCTs was slight across all outcomes (CCA range: 1–4%). While these CCA values represent the historical overlap among previously published SRs, the de novo meta-analyses of pooled effect estimates eliminated any duplicate primary studies. Consequently, zero primary study overlap exists for the pooled effect estimates reported in this review.

### 3.3. Risk of Bias Assessment

One SR was rated as high quality (11.1%) [27], two were rated as moderate (22.2%) [9,12], one was rated as low (11.1%) [28], and five were rated as of critically low quality (55.6%) [11,13,29,30,31]. The leading critical issues identified were AMSTAR 2 item 13—consideration of risk of bias when interpreting and discussing the results of the review, item 15—assessment and discussion of publication bias and its potential impact on the results, item 5—not performing study selection in duplicate, and item 8—inadequate description of included studies (Supplementary Table S4). Sources of bias among primary studies were mainly performance bias in RCTs, due to challenges in blinding, and comparability issues in non-RCTs, resulting from inadequate adjustment for confounding factors (Supplementary Tables S5 and S6).

### 3.4. Main Results

Eight SRs assessed mechanical ventilation duration, ICU length of stay, and ventilator-associated pneumonia [9,11,12,13,27,28,29,30], while hospital LOS was investigated in five reviews [11,12,27,28,30], and mortality was evaluated in all nine [9,11,12,13,27,28,29,30,31].

In RCTs, ET was associated with statistically significant reductions in mechanical ventilation duration (MD −3.76 days, 95% CI −6.01 to −1.52, *p* < 0.001), ICU LOS (MD −6.64 days, 95% CI −9.92 to −3.35, *p* < 0.001), and the odds of VAP (OR 0.66, 95% CI 0.46 to 0.94, *p* = 0.02). No statistically significant differences were observed for hospital LOS (MD −8.72 days, 95% CI −22.95 to 5.52, *p* = 0.23) or mortality (OR 0.84, 95% CI 0.68 to 1.05, *p* = 0.12).

In non-RCTs, ET was associated with statistically significant reductions across mechanical ventilation duration (MD −8.19 days, 95% CI −11.52 to −4.85, *p* < 0.001), ICU LOS (MD −8.59 days, 95% CI −10.54 to −6.63, *p* < 0.001), hospital LOS (MD −7.15 days, 95% CI −9.32 to −4.98, *p* < 0.001), and VAP (OR 0.58, 95% CI 0.47 to 0.71, *p* < 0.001). No statistically significant difference was observed for mortality (OR 1.03, 95% CI 0.81 to 1.30, *p* = 0.82).

Detailed effect estimates including number of pooled studies, heterogeneity metrics, publication bias evaluations, and GRADE certainty of evidence ratings are detailed in Table 2.

### 3.5. Subgroup Analyses

Among RCTs, results for mechanical ventilation duration remained stable in TBI and critically ill, while others showed no significant difference. Results for ICU LOS remained stable in critically ill, stroke, and trauma populations while others showed no significant difference. Results for hospital LOS remained stable in one RCT focusing on trauma, while one RCT focusing on stroke showed no significant difference. Results for VAP remained stable in the subgroup analysis of RCTs focusing on critically ill, while others showed no significant differences. Results for mortality remained stable within COVID-19, stroke, and trauma populations, while in critically ill the odds of death were lower in ET compared to LT (OR 0.71, 95% CI 0.53–0.95, *p* = 0.02). Subgroup-specific effect estimates, heterogeneity metrics, and publication bias assessments for evidence from RCTs are detailed in Table 3. In non-RCTs, observed reductions across mechanical ventilation duration, ICU stay, and hospital stay remained consistent across most clinical subgroups, except for patients with COVID-19 (Supplementary Table S7).

### 3.6. Sensitivity Analyses

In sensitivity analyses pooling only primary studies comparing ET (≤7 days) versus LT (>7 days) or no tracheostomy, pooled effect estimates for RCTs were consistent in direction and statistical significance with the primary models across all outcomes. Findings from observational studies were similarly stable, with the exception of VAP, which lost statistical significance (*p* = 0.05). Full numerical estimates are presented in Supplementary Table S8.

Due to heterogeneous mortality definitions (Supplementary Data S1), a sensitivity analysis restricted to RCTs reporting in-hospital and 60-day mortality was conducted. Results were consistent with the main findings (Supplementary Figure S20).

Findings from an included Bayesian meta-analysis [29] did not differ meaningfully from the frequentist results of this review, but rather provided confirmation using posterior probabilities of benefit. Specifically, the analysis demonstrated benefits favoring ET in mechanical ventilation duration (13 RCTs), ICU LOS (12 RCTs), and VAP (16 RCTs). Additionally, it indicated benefits favoring ET for short-term mortality across 18 RCTs, whereas hospital LOS was not assessed.

### 3.7. Reporting Bias

Based on evidence from RCTs, the pooled analysis of ICU LOS showed high risk of reporting bias, as both visual funnel plot asymmetry and small-study effects (*P_Egger_* = 0.04) were observed. While visual asymmetry was also observed for mechanical ventilation duration, the Egger’s test did not confirm small-study effects (*P_Egger_* = 0.12). Both visual and statistical assessments indicated a low risk of bias for VAP and mortality. Due to the limited number of studies (*n* = 2), the assessment for hospital LOS was considered underpowered to reliably detect reporting bias. Detailed funnel plots and Egger’s test results for non-RCTs and subgroup analyses are provided in the Supplementary Material (Supplementary Table S7 and Supplementary Figures S1–S19).

### 3.8. Certainty of Evidence

Applying the GRADE framework, the certainty of evidence ranged from moderate to very low depending on study design and outcome. Certainty of evidence from RCTs for mechanical ventilation duration and VAP was rated moderate. Mechanical ventilation duration was downgraded once due to very high unexplained heterogeneity (*I*^2^ = 90.54%) and VAP once for high risk of performance bias with a potential for detection bias among included studies. Certainty of evidence was low for ICU LOS, downgraded twice, as follows: once for high risk of performance and detection bias, and once for publication bias (funnel plot asymmetry; *P_Egger_* = 0.04). Findings regarding mortality and hospital LOS were supported by low and very low certainty evidence, respectively. Certainty of evidence from observational studies was very low across all outcomes, mainly because of inadequate adjustment for confounding among the included studies.

Complete GRADE evidence profiles, detailing domain-level judgments and explicit footnote justifications for all rating adjustments, are provided in Supplementary Table S9.

## 4. Discussion

The present umbrella review provides the most comprehensive evidence to date regarding the impact of tracheostomy timing on clinical outcomes across a broad spectrum of patient populations, including those with critical illness, COVID-19, TBI, stroke, trauma, and SCI. Meta-analyses of RCTs showed that ET reduces mechanical ventilation duration and ICU LOS with moderate certainty, and lowers the odds of VAP with low certainty, compared to LT across diverse populations. Findings from non-RCTs were consistent, suggesting that ET may also shorten hospital LOS, although with very low certainty. Neither RCTs nor observational studies demonstrated a difference in overall mortality, except in RCTs within the critically ill subgroup, where ET was associated with lower short-term mortality.

The physiological advantages of tracheostomy support these findings, as follows: reduced dead space, airway resistance, and work of breathing promote earlier weaning compared with translaryngeal intubation [10,35,36]. Reduced sedation needs, and the ability to initiate earlier mobilization and communication likely further contribute to shortened mechanical ventilation duration and ICU stays [36,37]. By decreasing the risk of VAP, ET may indirectly reduce ICU LOS.

The absence of differences in mechanical ventilation duration for the stroke and COVID-19 subgroups should be interpreted in the context of limited RCT data. For instance, evidence from RCTs for mechanical ventilation in stroke patients was limited to a single trial [38] of 60 participants (30 ET/30 LT). Although a trend toward reduced ventilation duration was observed, the effect estimate lacked precision, as reflected by the wide confidence interval. Notably, when the authors of the source review [11] pooled this trial alongside four observational studies, a statistically significant association between early tracheostomy and reduced mechanical ventilation duration was observed. Evidence from RCTs in COVID-19 patients was likewise restricted to a single trial (*n* = 150). Among COVID-19 patients, disease severity is a potential stronger determinant of ventilation duration than tracheostomy timing. Beyond the limited number of trials, inconsistent definitions of ET may have diluted a potential treatment effect of ET on mechanical ventilation duration in the trauma subgroup, as all three pooled trials had varying definitions of ET. In trauma populations, ICU-acquired polyneuropathy and challenges in identifying patients requiring prolonged ventilation may have further attenuated the association.

Subgroup analyses on ICU LOS in TBI and COVID-19 populations were statistically non-significant but were constrained to single RCTs [39,40], carrying a heightened risk of type II error.

The reduction in VAP associated with ET is plausible given the improved pulmonary hygiene, enhanced oral care, and shortened exposure to mechanical ventilation it enables [13,41]. Conversely, neutral VAP results in the TBI and trauma subgroups, which stem from the exact same four primary RCTs, may be explained by variations in VAP diagnostic criteria as well as inconsistent cutoffs used to define ET versus LT across trials. In COVID-19, the neutral results for ET on VAP were again based on a single trial. The impact of tracheostomy timing on mortality remains a debated topic in critical care, due to inconsistent evidence. The finding of a short-term survival benefit for the critically ill subgroup differs from the neutral results reported in the source SR [9]. This statistical discrepancy can be explained by a difference in between-study variance estimation; by estimating a lower absolute variance, the present model assigned greater relative weight to high-precision trials. These results are consistent with previous Bayesian [29] and Network meta-analyses [31], which tend to be more sensitive to treatment effects. The Bayesian analysis showed a short-term survival benefit only when ET was performed within four days, compared to tracheostomy at or beyond day 13. As most existing SRs and RCTs used definitions of ET and LT with seven or 10 days as cutoffs, these may have diluted the treatment effect by grouping responsive patients with those treated after the optimal biological window. ET may lower short-term mortality by reducing VAP through improved mucociliary clearance, minimizing sedation exposure, and facilitating earlier mobilization [29,31].

### 4.1. Study Limitations

This umbrella review has several strengths. Of 41 SRs initially included, the nine most comprehensive were selected for data synthesis. Overlap across primary studies was addressed through de-duplication, and data was stratified by study design (RCT vs. non-RCT) and clinical subgroup. This allowed a more granular synthesis across heterogeneous ICU populations. We also provided transparent assessments of methodological quality (AMSTAR-2) and certainty of evidence (GRADE).

Nonetheless, several limitations warrant caution. First, over half (55.6%) of the reviews included were rated as critically low quality on AMSTAR-2. Flaws in reviews’ methodological quality, such as a lack of pre-registered protocols or deviations from them, risk introducing procedural bias into secondary reporting. However, because this umbrella review conducted original meta-analyses using primary-study-level data, GRADE certainty assessments serve as the primary determinant of evidence confidence, mitigating reliance on secondary review quality. Second, the overall certainty of evidence ranged from moderate to very low, largely due to between-study heterogeneity and the inclusion of observational studies prone to confounding. Interpretation of observational evidence is particularly limited by confounding by indication and selection bias, as patients with better prognoses are more likely to be selected for ET. Conversely, LT cohorts are susceptible to immortal-time bias, as patients must survive long enough to reach the late tracheostomy threshold. These biases confound the effects of tracheostomy timing with patient stability in absence of a robust causal inference framework. Third, substantial heterogeneity (*I*^2^ > 50%) was present in 90% of meta-analyses. Although heterogeneity remained high when pooling only studies with a timing threshold of ≤7 days, pooled effect estimates remained consistent in both direction and statistical significance in accordance with the primary analyses. This indicates that while timing-definition variability exists, it does not invalidate the primary pooled estimates. An inherent limitation of this umbrella review is the heterogeneity of outcome definitions across primary studies. Because the analysis relied on summary data reported by the included systematic reviews, study-level endpoints (e.g., diagnostic criteria for VAP) could not be individually harmonized, contributing to the observed heterogeneity. Stratification by clinical diagnosis (Table 3 and Supplementary Table S7) reduced heterogeneity for mortality and VAP, though it remained high for continuous outcomes such as mechanical ventilation duration and LOS. Meta-regression was not feasible due to the small number of RCTs per subgroup, which limited the statistical power to explore sources of heterogeneity.

### 4.2. Recommendations for Future Research

Future studies should establish standardized definitions of tracheostomy timing, incorporating diagnosis-specific criteria where clinically indicated to identify the optimal temporal window that maximizes clinical benefit. In absence of such standardized definitions, using cut-offs of ≤7 days for early and >7 days for late tracheostomy is recommended, as it aligns with most of the existing evidence. Primary studies on tracheostomy timing should prioritize high-quality RCTs, or if not feasible, observational studies with proper and transparent causal inference methods in specific diagnostic groups, where high-quality evidence is scarce (e.g., TBI, Stroke, SCI). Future systematic reviews focusing on such underrepresented subgroups should only be conducted once a substantial quantity of new high-quality primary studies has been published to avoid redundant syntheses.

## 5. Conclusions

Overall, results from meta-analyses indicate reductions in ventilator-associated pneumonia and mechanical ventilation duration for early tracheostomy in mixed-etiology cohorts, with moderate certainty. Early tracheostomy may be associated with shorter ICU length of stay, while the effect of early tracheostomy on hospital length of stay and mortality remains uncertain. As such, optimal tracheostomy timing remains patient- and context-dependent. Future research should aim to standardize definitions of tracheostomy timing and identify the optimal temporal window that maximizes clinical benefit. Future research should prioritize high-quality, etiology-specific RCTs in underrepresented cohorts (e.g., TBI, stroke, SCI). Further systematic reviews are only warranted once a substantial volume of such primary evidence has been published.

## Figures and Tables

**Table 1:** Characteristics of systematic reviews included in the evidence synthesis.

Authors, Year	Country	Population	No. of Included Studies (Design)	ET Definition	Comparator Groups	Health Outcomes	AMSTAR-2 Rating
Chorath et al., 2021 [9]	United States	Critically Ill *	17 (RCTs only)	≤7 days	ET vs. LT	VAP, MV duration, ICU LOS, Mortality	Moderate
Quinn et al., 2022 [29]	United Kingdom	Critically Ill *	19 (RCTs only)	<10 days	ET vs. LT	Mortality, VAP, ICU LOS, Hospital LOS, MV duration	Critically low
Kishihara et al., 2023 [31]	Japan	Critically Ill *	8 (RCTs only)	Not applicable	Tracheostomy timing	Mortality	Critically low
Cai et al., 2017 [13]	China	Trauma	20 (6 RCTs; 14 non-RCTs)	≤7 days	ET vs. LT/PI	Mortality, ICU LOS, Hospital LOS, MV duration, Pneumonia	Critically low
Villemure-Poliquin et al., 2020 [28]	Canada	Trauma (non-neurological)	9 (RCTs only)	<10 days	ET vs. LT/PI	Mortality, ICU LOS, Hospital LOS, MV duration, VAP	Low
Foran et al., 2022 [30]	Canada	Spinal Cord Injury	17 (non-RCTs only)	≤10 days	ET vs. LT/PI	Mortality, MV duration, ICU LOS, Hospital LOS, VAP	Critically low
Bertini et al., 2023 [12]	Italy	Traumatic Brain Injury	19 (5 RCTs; 14 non-RCTs)	≤14 days	ET vs. LT	MV duration, VAP, ICU LOS, Hospital LOS, Mortality	Moderate
Qiu et al., 2023 [11]	China	Stroke	9 (2 RCTs; 7 non-RCTs)	≤7 days	ET vs. LT	Mortality, Hospital LOS, ICU LOS, MV duration, VAP	Critically low
Szafran et al., 2023 [27]	Germany	COVID-19	25 (1 RCT; 24 non-RCTs)	≤10 days	ET vs. LT	Mortality, MV duration, VAP, ICU LOS, Hospital LOS	High

* No definition of the critically ill population was provided. AMSTAR-2 = a measurement tool to assess the methodological quality of systematic reviews; COVID-19 = coronavirus disease 2019; ET = early tracheostomy; ICU = intensive care unit; LOS = length of stay; LT = late tracheostomy; MV = mechanical ventilation; PI = prolonged intubation; RCT = randomized controlled trial; VAP = ventilator-associated pneumonia.

**Table 2:** Pooled effect estimates and certainty of evidence for early versus late tracheostomy in mechanically ventilated patients of mixed etiology.

Outcome	No. of Studies	Effect Estimate (95%CI)	*P*	*I* ^2^	*P_het_*	tau^2^	Publication Bias (*P_Egger_*) ^a^	Certainty of Evidence (GRADE)
Evidence from RCTs								
MV Duration (days)	16	MD −3.76 (−6.01 to −1.52)	<0.001	90.5%	<0.001	16.41	0.12	⊕⊕⊕◯ Moderate
ICU LOS (days)	13	MD −6.64 (−9.92 to −3.35)	<0.001	96.4%	<0.001	28.16	0.04	⊕⊕◯◯ Low
Hospital LOS (days)	2	MD −8.72 (−22.95 to 5.52)	0.23	83.0%	0.02	89.16	-	⊕◯◯◯ Very low
VAP	19 ^b^	OR 0.66 (0.46 to 0.94)	0.02	56.7%	<0.001	0.30	0.42	⊕⊕⊕◯ Moderate
Mortality	22	OR 0.84 (0.68 to 1.05)	0.13	41.5%	0.02	0.09	0.71	⊕⊕◯◯ Low
Evidence from non-RCTs								
MV Duration (days)	27	MD −8.19 (−11.52 to −4.85)	<0.001	98.5%	<0.001	67.53	0.60	⊕◯◯◯ Very low
ICU LOS (days)	30	MD −8.59 (−10.54 to −6.63)	<0.001	97.1%	<0.001	22.14	0.61	⊕◯◯◯ Very low
Hospital LOS (days)	22	MD −7.15 (−9.32 to −4.98)	<0.001	71.4%	<0.001	12.09	0.05	⊕◯◯◯ Very low
VAP	31 ^c,d^	OR 0.58 (0.47 to 0.71)	<0.001	62.8%	<0.001	0.14	0.05	⊕◯◯◯ Very low
Mortality	42 ^e^	OR 1.03 (0.81 to 1.30)	0.82	55.7%	<0.001	0.21	0.46	⊕◯◯◯ Very low

^a^ Publication bias was assessed through funnel plot symmetry (Supplementary Figures S1–S9) and Egger’s regression test, with *p* < 0.10 indicating significant asymmetry (small-study effects). ^b^ The study by Dunham (1984) [32] was removed from this analysis as the outcome was respiratory sepsis. ^c,e^ The study by Elkbuli (2019) [33] was removed from this analysis due to an expression of concern. ^d^ The study by Rizk (2011) [34] was removed from this analysis as the outcome was a composite measure of pulmonary occurrence. ⊕⊕⊕◯ Moderate certainty; ⊕⊕◯◯ Low certainty; ⊕◯◯◯ Very low certainty; CI = confidence interval; GRADE = Grading of Recommendations Assessment, Development and Evaluation; *I*^2^ = inconsistency (heterogeneity) index; ICU = intensive care unit; LOS = length of stay; MD = mean difference; MV = mechanical ventilation; OR = odds ratio; *P_Egger_* = *p*-value for Egger’s test of publication bias; *P_het_* = *p*-value for heterogeneity; RCT = randomized controlled trial; tau^2^ = between-study variance; VAP = ventilator-associated pneumonia.

**Table 3:** Estimates for early versus late tracheostomy stratified by clinical diagnosis: evidence from randomized controlled trials.

Population	Outcome	No. of Studies	Effect Estimate (95% CI)	*P*	*I* ^2^	*P_het_*	tau^2^	Publication Bias (*P_Egger_*) ^a^
Critically Ill	MV Duration (days)	11	MD −2.96 (−5.61 to −0.31)	0.03 ^b^	92.02%	<0.001	15.88	0.35
	ICU LOS (days)	9	MD −7.52 (−12.29 to −2.75)	<0.001	95.35%	<0.001	42.76	-
	VAP	15	OR 0.60 (0.40 to 0.90)	0.01	64.68%	<0.001	0.36	0.22
	Mortality	12	OR 0.71 (0.53 to 0.95)	0.04 ^c^	45.25%	<0.001	0.09	0.40
Traumatic Brain Injury	MV Duration (days)	3	MD −4.12 (−6.42 to −1.82)	<0.001	0.00%	0.60	0.00	-
	ICU LOS (days)	1	MD −3.00 (−7.27 to −1.27)	0.17	-	-	-	-
	VAP	4	OR 0.97 (0.52 to 1.79)	0.91	0.00%	0.79	0.00	-
	Mortality	5	OR 2.01 (0.81 to 4.99)	0.13	33.53%	0.20	0.35	-
Stroke	MV Duration (days)	1	MD 1.93 (−1.03 to 4.89)	0.20	-	-	-	-
	ICU LOS (days)	2	MD −1.71 (−2.60 to −0.82)	<0.001 ^d^	48.18%	0.16	0.24	-
	Hospital LOS (days)	1	MD −2.35 (−6.33 to 1.63)	0.25	-	-	-	-
	Mortality	3	OR 0.68 (0.26 to 1.77)	0.43	75.83%	0.02	0.53	-
Trauma	MV Duration (days)	3	MD −8.37 (−18.10 to 1.35)	0.09	90.80%	<0.001	66.80	-
	ICU LOS (days)	1	MD −21.00 (−29.36 to −12.64)	<0.001	-	-	-	-
	Hospital LOS (days)	1	MD −4.25 (−4.94 to −3.56)	<0.001	-	-	-	-
	VAP	4 ^e^	OR 0.97 (0.52 to 1.79)	0.91	0.00%	0.79	0.00	-
	Mortality	4	OR 1.17 (0.48 to 2.87)	0.73	46.34%	0.13	0.37	-
Trauma (non-neurological)	MV Duration (days)	4	MD −2.25 (−8.41 to 3.90)	0.47	96.46%	<0.001	37.43	-
	ICU LOS (days)	3	MD −5.69 (−12.85 to 1.46)	0.12	91.57%	<0.001	35.70	-
	Mortality	9	OR 0.82 (0.66 to 1.01)	0.07	22.60%	0.24	0.02	-
COVID-19	MV Duration (days)	1	MD −1.50 (−5.76 to 2.76)	0.49	-	-	-	-
	ICU LOS (days)	1	MD −0.50 (−5.33 to 4.33)	0.84	-	-	-	-
	VAP	1	OR 1.09 (0.21 to 5.57)	0.92	-	-	-	-
	Mortality	1	OR 0.74 (0.38 to 1.47)	0.39	-	-	-	-

^a^ Publication bias was assessed through funnel plot symmetry (Supplementary Figures S10–S12) and Egger’s regression test, with *p* < 0.10 indicating significant asymmetry (small-study effects). ^b^ This parameter was no longer statistically significant (*p* = 0.06) in a sensitivity analysis using a random-effects model with restricted maximum likelihood (REML) estimation and Knapp-Hartung standard error adjustments. ^c^ This parameter was no longer statistically significant (*p* = 0.06) in a sensitivity analysis using a random-effects model with restricted maximum likelihood (REML) estimation and Knapp–Hartung standard error adjustments. ^d^ This parameter was no longer statistically significant (*p* = 0.17) in a sensitivity analysis using a random-effects model with restricted maximum likelihood (REML) estimation and Knapp–Hartung standard error adjustments. ^e^ The study by Dunham (1984) [32] was removed from this analysis as the outcome was respiratory sepsis. CI = confidence interval; *I*^2^ = inconsistency (heterogeneity) index; ICU = intensive care unit; LOS = length of stay; MD = mean difference; MV = mechanical ventilation; OR = odds ratio; *P_Egger_* = *p*-value for Egger’s test of publication bias; *P_het_* = *p*-value for heterogeneity; RCT = randomized controlled trial; tau^2^ = between-study variance; VAP = ventilator-associated pneumonia.

## Data Availability

All data generated or analyzed during this study are included in this published article and its Supplementary Information Files.

## Supplementary Materials

The following supporting information can be downloaded at: https://www.mdpi.com/article/10.3390/jcm15176685/s1, Supplement Table S1. Selection Matrix and Rationale for Systematic Reviews Included in Quantitative Synthesis Across Clinical Populations; Supplement Table S2. Characteristics of excluded studies; Supplement Table S3. General characteristics of included systematic reviews and meta-analyses; Supplement Table S4. Critical appraisal of included systematic reviews and meta-analyses in synthesis based on AMSTAR-2 domains; Supplement Table S5. Risk of bias assessment of primary randomized controlled trials identified within the included systematic reviews using the Cochrane risk of bias tool (RoB 1); Supplement Table S6. Risk of bias assessment of primary non-randomized studies identified within the included systematic reviews using the Newcastle-Ottawa Scale (NOS); Supplement Table S7. Estimates for early versus late tracheostomy stratified by clinical diagnosis: evidence from observational studies; Supplement Table S8. Pooled effect estimates for early (≤7 days) versus late (>7 days) or no tracheostomy in patients with heterogeneous causes of critical illness; Supplement Table S9. Certainty of evidence assessment for early versus late tracheostomy using the GRADE framework; Supplement Table S10. PRISMA 2020 Checklist; Supplement Figure S1. Funnel plot of standard error by mean difference for mechanical ventilation duration (evidence from RCTs); Supplement Figure S2. Funnel plot of standard error by mean difference for ICU length of stay (evidence from RCTs); Supplement Figure S3. Funnel plot of standard error by log odds ratio for ventilator-associated pneumonia (evidence from RCTs); Supplement Figure S4. Funnel plot of standard error by log odds ratio for mortality (evidence from RCTs); Supplement Figure S5. Funnel plot of standard error by mean difference for mechanical ventilation duration (evidence from non-RCTs); Supplement Figure S6. Funnel plot of standard error by mean difference for ICU length of stay (evidence from non-RCTs); Supplement Figure S7. Funnel plot of standard error by mean difference for hospital length of stay (evidence from non-RCTs); Supplement Figure S8. Funnel plot of standard error by log odds ratio for ventilator-associated pneumonia (evidence from non-RCTs); Supplement Figure S9. Funnel plot of standard error by log odds ratio for mortality (evidence from non-RCTs); Supplement Figure S10. Funnel plot of standard error by mean difference for mechanical ventilation duration: Subgroup analysis of critically ill patients (evidence from RCTs); Supplement Figure S11. Funnel plot of standard error by log odds ratio for ventilator-associated pneumonia: Subgroup analysis of critically ill patients (evidence from RCTs); Supplement Figure S12. Funnel plot of standard error by log odds ratio for mortality: Subgroup analysis of critically ill patients (evidence from RCTs); Supplement Figure S13. Funnel plot of standard error by mean difference for ICU length of stay: Subgroup analysis of traumatic brain injury patients (evidence from non-RCTs); Supplement Figure S14. Funnel plot of standard error by mean difference for hospital length of stay: Subgroup analysis of traumatic brain injury patients (evidence from non-RCTs); Supplement Figure S15. Funnel plot of standard error by log odds ratio for ventilator-associated pneumonia: Subgroup analysis of traumatic brain injury patients (evidence from non-RCTs); Supplement Figure S16. Funnel plot of standard error by log odds ratio for mortality: Subgroup analysis of traumatic brain injury patients (evidence from non-RCTs); Supplement Figure S17. Funnel plot of standard error by log odds ratio for mortality: Subgroup analysis of COVID-19 patients (evidence from non-RCTs); Supplement Figure S18. Funnel plot of standard error by log odds ratio for ventilator-associated pneumonia: Subgroup analysis of spinal cord injury patients (evidence from non-RCTs); Supplement Figure S19. Funnel plot of standard error by log odds ratio for short-term mortality: Subgroup analysis of spinal cord injury patients (evidence from non-RCTs); Supplement Figure S20. Forest plot of early versus late tracheostomy for mortality, restricted to RCTs reporting in-hospital mortality and 60-day follow-up.

## Abbreviations

CCA	Corrected Covered Area
ICU	Intensive Care Unit
ET	Early Tracheostomy
LT	Late Tracheostomy
SR	Systematic Review
VAP	Ventilator-associated Pneumonia
TBI	Traumatic Brain Injury
SCI	Spinal Cord Injury
LOS	Length of Stay
