# Supplementary material for: Early Versus Late Tracheostomy: An Umbrella Review and Meta-Analysis
Source: J Clin Med. 2026 Aug 28;15(17):6685. doi: 10.3390/jcm15176685 (PMC13566620; doi:10.3390/jcm15176685)
Supplement: Supplementary file 1 [file jcm-15-06685-s001.zip › jcm-4451223-supplementary/5_Supplemental Material.pdf]

Table of Contents

Supplement Table S1 .... **2**
Supplement Table S2 4
Supplement Table S3 7
Supplement Table S4 21
Supplement Table S5 24
Supplement Table S6 26
Supplement Table S7 28
Supplement Table S8 29
Supplement Table S9 30
Supplement Table S10 31
Supplement Figure S1 34
Supplement Figure S2 34
Supplement Figure S3 35
Supplement Figure S4 35
Supplement Figure S5 36
Supplement Figure S6 36
Supplement Figure S7 37
Supplement Figure S8 37
Supplement Figure S9 38
Supplement Figure S10 39
Supplement Figure S11 39
Supplement Figure S12 40
Supplement Figure S13 40
Supplement Figure S14 41
Supplement Figure S15 41
Supplement Figure S16 42
Supplement Figure S17 42
Supplement Figure S18 43
Supplement Figure S19 43
Supplement Figure S20 44
References 45

Supplement Table S1 Selection Matrix and Rationale for Systematic Reviews Included in Quantitative Synthesis Across Clinical Populations

Population	Included SRs by type of primary studies (n = 41)	Used for Synthesis (n = 9)	Reason for Selection
**Critically Ill**	**Mixed:**<br>Adly 2018<br>Maziak 1998<br><br>**Interventional:**<br>Kishihara 2023<br>Quinn 2022<br>Chorath 2021<br>Deng 2021<br>Wang 2019<br>Meng 2016<br>Andriolo 2015<br>Hosokawa 2015<br>Siempos 2015<br>Szakmany 2015<br>Liu CC 2014<br>Liu X 2014<br>Huang 2014<br>Gomes Silva 2012<br>Wang 2011<br>Griffiths 2005<br><br>**Observational:**<br>Shan 2013	**Chorath 2021**<br>**Kishihara 2023**<br>**Quinn 2022**	**Chorath 2021**<br>Selected as the primary SR for this population, as it was the newest and most comprehensive SR using frequentist pairwise MA<br><br>**Kishihara 2023 & Quinn 2022**<br>Mainly included to compare findings from other methodological approaches (network MA and Bayesian MA) with those from frequentist pairwise MA (Chorath 2021)
**Traumatic Brain Injury (TBI)**	**Mixed:**<br>Bertini 2023<br>Marra 2021<br>Lu 2018<br><br>**Interventional:**	**Bertini 2023**	Newest and most comprehensive SR for this population

	-<br><br>**Observational:**<br>De Franca 2020<br>Selvakumar 2022		
**Stroke**	**Mixed:**<br>Qiu 2023<br>Premraj 2023<br>De Franca 2021<br><br>**Interventional:**<br>-<br><br>**Observational:**<br>**-**	**Qiu 2023**	**Qiu 2023** was selected over Premraj 2023 for this population, as it synthesized on a much broader set of clinical relevant outcomes, including VAP and duration of mechanical ventilation compared to Premraj 2023, which focused mainly on mortality
**Acute Brain Injury**	**Mixed:**<br>Wahlster 2021<br><br>**Interventional:**<br>McCredie 2017<br><br>**Observational:**<br>Tavares 2023	**None**	No SR was selected for synthesis from this population as there was too much overlap with the stroke and TBI SRs
**Trauma**	**Mixed:**<br>Dunham 2016<br>Cai 2017<br><br>**Interventional:**<br>Villemure-Poliquin 2020<br><br>**Observational:**<br>**-**	**Cai 2017**<br>**Villemure-Poliquin 2020**	**Cai 2017** was selected for the general trauma population as this review was the newest and most comprehensive on a general trauma population

			**Villemure-Poliquin 2020** was selected because it focused on non-neurological injured trauma patients
**Spinal Cord Injury**	**Mixed:**<br>-<br><br>**Interventional:**<br>-<br><br>**Observational:**<br>Foran 2022<br>Mubashir 2021	**Foran 2022**	**Foran 2022** was selected for the spinal cord injury population as this review was the newest and most comprehensive for this population
**COVID-19**	**Mixed:**<br>Szafran 2023<br>Ferro 2021<br>Staibano 2021<br><br>**Interventional:**<br>-<br><br>**Observational:**<br>Battaglini 2022<br>Chong 2022<br>Ji 2022	**Szafran 2023**	**Szafran 2023** was selected for the COVID-19 population as this review was the newest and most comprehensive for this population

Supplement Table S2 Characteristics of excluded studies

Study	Reason for exclusion
Ansems 2023(1)	Conference Abstract

Arora 2012(2)	Wrong study design
Baron 2016(3)	Wrong study design
Barquist 2006(4)	Wrong study design
Bause 1999(5)	Wrong study design
Benito 2021(6)	Wrong intervention
Bhasarkar 2023(7)	Wrong study design
Bittner 2012(8)	Wrong study design
Blackwood 2014(9)	Wrong intervention
Blot 2008(10)	Wrong study design
Burns 2009(11)	Wrong intervention
Burns 2014(12)	Wrong intervention
Camacho 2014(13)	Wrong intervention
Cirillo 2015(14)	Wrong study design
Cox 2012(15)	Wrong study design
Craven 2022(16)	Wrong study design
Damas 2015(17)	Wrong study design
Damuth 2015(18)	Wrong intervention
Delaney 2006(19)	Wrong intervention
Dettmer 2015(20)	Conference abstract
Devanand 2024(21)	Wrong study design
Ding 2017(22)	Wrong intervention
Dunham 2014(23)	Wrong study design
Filice 2021(24)	Wrong study design
Freeman 2000(25)	Wrong intervention
Gadkaree 2016(26)	Wrong study design
Gandia-Martinez 2010(27)	Wrong study design
Garrubba 2009(28)	Wrong intervention
Gastmeier 2007(29)	Wrong intervention
Ghanima 2024(30)	Wrong study design
Goligher 2009(31)	Wrong study design
Groves 2007(32)	Wrong study design
Gupta 2020(33)	Wrong study design
He 2024(34)	Wrong comparator
Herritt 2018(35)	Wrong study design
Jerjes 2011(36)	Wrong study design
Johnson-Obaseki 2016(37)	Wrong intervention
Khan 2020(38)	Wrong intervention

Lamb 2020(39)	Wrong study design
Lou 2023(40)	Wrong intervention
Lu 2011(41)	Translation impossible
Mohammadi 2021(42)	Wrong study design
Muscedere 2011(43)	Wrong intervention
Nkwonta 2017(44)	Conference Abstract
Ramirez 2007(45)	Wrong study design
Roquilly 2015(46)	Wrong outcomes
Saffle 2002(47)	Wrong study design
Schreiber 2021(48)	Conference abstract
Sharma 2021(49)	Wrong intervention
Shen 2022(50)	Wrong study design
Szakmany 2013(51)	Wrong study design
TheEditorsOfTheLancetRespiratory 2015(52)	Retracted manuscript
Toeg 2017(53)	Wrong outcomes
Wu 2019(54)	Translation impossible
Zheng 2012(55)	Wrong study design

Supplement Table S3 General characteristics of included systematic reviews and meta-analyses

Author, Year	Country	Objective	Inclusion Criteria	Meta-analysis (yes/no)	Type of included studies	Protocol registration (Yes/No)	Number of included studies	Literature search time span	Databases Searched	Population	Definition of exposure	Comparator groups	Health outcomes	Narrative synthesis
**Araujo de Franca** et al., 2021**(56)**	BR	Assess the impact of ET on stroke patients' outcomes during hospitalization.	Articles written in English, French, Spanish, and Portuguese, that associated ET and LT with, at least, one relevant clinical outcome	Yes	Observational and interventional	No	3	From inception - 2018	Bireme, PubMed, and Cochrane	Stroke<br><br>hemorrhagic (97) or ischemic (11)	NA	ET vs LT	Primary outcome: VAP rates<br><br>Secondary outcomes: MV duration, ICU and hospital LOS, Mortality	NA
**Battaglini** et al., 2022**(57)**	IT	Summarise evidence on overall mortality in patients with COVID-19 and tracheostomy, investigating tracheostomy timing and technique	Adult patients with suspected or confirmed SARS-CoV-2; COVID-19 patients who received a tracheostomy during their ICU course;<br>20 or more patients.	Yes	Observational	Yes	47	2020 - 2022	PubMed, Embase, Scopus, and the Cochrane trial registry	Adults with COVID-19<br><br>Not further defined	Main analysis time to tracheostomy (continuous)<br><br>Sensitivity analysis: ET = <14 or <16.5 d LT = >= 14 or >=16.5 d	Main analysis time to tracheostomy (continuous)<br><br>Sensitivity analysis: ET vs LT	Primary outcome: all-cause mortality<br><br>Secondary outcomes: ICU and hospital LOS, duration of MV	NA
**Liu, C. C. et al., 2014(58)**	CA	Investigate whether ET leads to improved outcomes compared with LT	Randomized or quasirandomized study, critically ill patients requiring prolonged MV, at least one of the relevant outcomes	Yes	Interventional	Yes	11	1983-2013	MEDLINE (including PubMed) and Embase	Critical care (mixed)	For subgroup analysis only:<br>ET ≤ 3 days, 4-5 days, 7-8 days	ET vs LT	Pneumonia Incidence, Length of MV, ICU LOS, Hospital mortality	NA
**De Franca et al., 2020(59)**	BR	Elucidate the impact of early	English, French, Spanish, or	Yes	Observational	No	8	From inception - 2018	Lilacs, PubMed, and Cochrane databases	Traumatic brain injury	NA	ET vs. LT	ICU stay, hospital stay,	NA

		tracheostomy on hospitalization outcomes in patients with traumatic brain injury	Portuguese studies; with: TBI as the base trauma; clearly formulated RQ; patient's admission assessment; minimum follow-up during hospital stay; and minimum two relevant outcomes.							Not further defined			mortality rates, ventilator-associated pneumonia (VAP)	
**McCredie et al., 2017(60)**	CA	Determine effects of ET compared to LT or prolonged Intubation on long-term, all-cause mortality in acutely brain-injured patients	RCTs and quasi-randomized trials comparing ET to LT or prolonged intubation in intubated adults with acute brain injury. Reported all-cause mortality	Yes	Interventional	Yes	10	From inception - 2015	MEDLINE, EMBASE, CINAHL, CENTRAL, and Web of Science	Acute brain injury	ET ≤ 10 days LT > 10 days	ET vs. LT/PI	Primary outcome: Long-term mortality<br><br>Secondary outcomes: Short-term mortality, Hospital mortality, ICU mortality, VAP, Duration of MV, ICU LOS	NA
**Mubashir et al., 2021(61)**	USA	Assess the optimal timing of tracheostomy in SCI patients and evaluate the potential benefits of early versus late tracheostomy	English studies with adult acute traumatic SCI patients, who underwent subsequent early or late percutaneous or surgical tracheostomy. No concomitant TBI in the study population	Yes	Observational and interventional	Yes	8	From inception - 2019	Medline (Ovid), PubMed, Embase, Cochrane Central, Cochrane Database of Systematic Reviews, and PsycINFO	adult patients (age ≥18 years) with acute traumatic SCI	ET was defined as either <7 or <10 days (≤7 or ≤10 days) after patient admission or after initiation of mechanical ventilation	ET vs. LT	primary outcomes hospital mortality and ICU LOS<br><br>Secondary outcomes Duration of MV, pneumonia rates	NA
**Foran et al., 2022(62)**	CA	Evaluate and synthesize evidence regarding the	RCTs that compared either the timing of tracheostomy,	Yes	Observational	Yes	17 (15 cohort 2 case	From inception - 2020	MEDLINE, EMBASE,	acute SCI patients	NA	ET vs LT/PI	Primary outcome: Short-term mortality	NA

		timing of tracheostomy in patients with acute traumatic SCI	or tracheostomy and PI, in patients with acute SCI, and cohort studies that included acute SCI patients receiving ET or LT/PI in the ICU				studies, Beom 2018 and Bellamy 1973)		CINAHL, Scopus, Web of Science, and CENTRAL				Secondary outcomes: MV duration, ICU LOS, Hospital LOS, VAP incidence	
**Marra et al., 2021(63)**	IT	Evaluate the effects of timing (early vs. late) of tracheostomy on mortality and incidence of VAP in traumatic brain-injured patients	English language; TBI as the main cause of trauma; clear outcome; reliable patient's admission assessment; LT clearly defined and not confused with prolonged intubation; and a minimum of two relevant outcomes	Yes	Observational and interventional	No	9	NA	PubMed	TBI<br><br>Not further defined	NA	ET vs. LT	ICU stay, hospital stay, MV duration, mortality rates, VAP	NA
**Wahlster et al., 2021(64)**	USA	Synthesize reported long-term outcomes in patients undergoing tracheostomy after severe acute brain injury (SABI)	adult (age>18 years) patients who underwent tracheostomy after SABI	Yes	Observational and interventional	Yes	19	1990-2019	PubMed, EMBASE and the Cochrane Library	SABI<br><br>defined as stroke, including acute ischemic stroke (AIS), intracranial hemorrhage (ICH) and aneurysmal subarachnoid hemorrhage (SAH), including TBI and HIE	NA	ET vs LT	Short-term mortality	NA
**Bertini et al., 2023(65)**	IT	To compare the effects of ET vs. LT on the MV duration in patients with TBI	all studies that mentioned tracheostomy for TBI in critically ill patients	Yes	Observational and interventional	Yes	19	From inception - 2022	MEDLINE, Scopus, Web Of Science, Cochrane Library	TBI<br><br>Not further defined	NA	ET vs. LT	Main outcomes: MV duration and VAP<br><br>Secondary outcomes: ICU LOS,	NA

													Hospital LOS, mortality.	
**Premraj et al., 2023(66)**	IT	Investigate the impact of tracheostomy timing on all-cause mortality in patients with severe stroke	peer-reviewed publications, preprints, and published abstracts including ICU-admitted stroke patients who received a tracheostomy during their stay and >20 patients. Studies with paediatric population and unknown timing of tracheostomy were excluded	Yes	Observational and interventional	Yes	13	From inception – 2022	PubMed, Google Scholar, EMBASE, Scopus, and the Cochrane trial registry	ICU-admitted patients who had stroke (either acute ischaemic stroke, AIS or intracerebral haemorrhage, ICH)<br><br>Stroke type was represented by the ratio of acute ischaemic stroke to intracerebral haemorrhage (AIS: ICH) in each study	mean time between initiation of MV and tracheostomy (days).	Mean time to tracheostomy	Primary outcome: all-cause mortality<br><br>Secondary outcomes: MV duration, ICU LOS and hospital LOS.	No
**Selvakumar et al., 2022(67)**	USA	Determine the impact of early, intermediate, and late tracheostomy in adult trauma patients with severe TBI on outcomes	English Cohort studies investigating the impact of tracheostomy timing on clinical outcomes of adult severe TBI patients	No	Observational	No	9	From inception - 2021	PubMed, Google Scholar, EMBASE, MedLine, Web of Science, Cochrane, and CINAHL	Adult severe TBI patients<br><br>Patients ≥16 years of age who received a tracheostomy following a clinical diagnosis of severe TBI (using either GCS ≤ 8 or head AIS ≥ 4).	NA	Early, intermediate, late tracheostomy	ICU-LOS, duration MV, VAP and in-hospital mortality	Yes
**Qiu et al., 2023(68)**	CN	Compare efficacy and safety between ET and LT in stroke patients	RCTs and observational studies. Adult Patients with stroke	Yes	Observational and interventional	Yes	9	From inception - 2023	Embase, PubMed, and the Cochrane Library	Adult patients with either ischemic or hemorrhagic stroke (including	ET (≤7 days) and LT (＞7)	ET vs. LT	Mortality, Hospital LOS, ICU LOS, MV duration, VAP	No

			who received tracheostomy intervention during prolonged mechanical ventilation							intracerebral hemorrhage, subarachnoid hemorrhage)				
**Wang et al., 2019(69)**	USA	Assess tracheotomy timing for critically ill ventilated patients and determine the outcomes' reliability	RCTs, mechanically ventilated patients, an intervention that includes ET for the treatment of critically ill patients receiving MV compared to LT, and the report of at least one relevant outcome	Yes	Interven tional	No	18	1984 - 2017	PubMed, EMBASE, and Cochrane	Critically ill patients<br><br>Not further defined	NA	ET vs LT	VAP, ICU LOS, mortality, duration of MV.	No
**Tavares et al., 2023(70)**	BR	Ascertain if the benefit of ET can be found in patients with severe TBI and stroke and if the benefit will remain across pathologies.	Retrospective / Prospective studies, and case series written in English, French, Spanish, or Portuguese; patients suffered exclusively from head injury or diagnosed with stroke; minimum follow-up during hospital stay; PI was not considered as the LT group, and a minimum of two relevant outcomes	Yes	Observa tional	Yes	11	NA	Lilacs, PubMed, and Cochrane	Severe TBI and stroke<br><br>Glasgow Coma Scale (GCS) <=8 at admission for a patient to be considered as severe TBI	NA	ET vs LT	ICU LOS, hospital LOS, mortality rates, or VAP diagnosis.	Yes
**Ferro et al., 2021(71)**	UK	To determine whether the timing of tracheostomy post-intubation	Patients diagnosed with COVID-19 who had undergone tracheostomy; contained outcome data	Yes	Observa tional	No	3	2019-2021	MEDLINE, PubMed Central, Embase, the Web of Science Core Collection and	COVID-19	Outcome data were pooled only for those studies	ET. vs LT	mortality	No

		influences outcomes	specific to tracheostomy on mortality, decannulation, mechanical weaning, and complications						the Cochrane Library		defining early tracheostomy as <14 days post-intubation			
**Adly et al., 201(72)**	EG	Evaluate the appropriate timing of tracheostomy in patients with prolonged intubation regarding relevant outcomes	English language to identify prospective, randomized controlled studies assessing the appropriate timing of tracheostomy in prolonged intubated patients	Yes	Observational and interventional	No	43<br>(41 adults)<br>(2 pediatric)	between 1987 and 2017	Medline	Patients with prolonged endotracheal intubation	Sensitivity analysis (ET= within 7d, 14d, 21d)	ET vs. LT	Incidence of hospital-acquired pneumonia, mortality, ICU LOS and duration of MV	No
**Andriolo et al., 2015(73)**	BR	Evaluate the effectiveness and safety of ET vs LT in critically ill adults predicted to be on prolonged mechanical ventilation with different clinical conditions	All RCTs or QRCTs comparing ET against LT for critically ill patients	Yes	Interventional	Yes	8	1966-2013	CENTRAL, MEDLINE, EMBASE, LILACS, PEDro, CINAHL	Critically ill adults (>= 18 years) expected to be on prolonged mechanical ventilation (24 hours to 21 consecutive days, six or more hours per day)	ET 2 - 10 days after tracheal intubation	ET vs. LT	Primary outcomes Mortality, MV duration<br>Secondary outcomes ICU LOS, VAP	Yes
**Chong and Tan, 2022(74)**	SGP	To determine clinical and resource utilization benefits of ET versus LT among COVID-19 patients	Studies of critically ill COVID-19 adults (age >18 years) undergoing ET or LT; which required IMV for respiratory failure and had a definition of ET at a cutoff of 10 or 14 days after IMV	Yes	Observational	Yes	12	2020-2021	Pubmed, Cochrane Library, Scopus, and Embase	Critically ill COVID-19 adults (age >18 years)<br>Diagnosis of COVID-19 was made by reverse transcriptase-polymerase chain reaction (RT-PCR) in	The definitions of early and late tracheostomy were based on the definitions of each study<br>(10 or 14d)	ET vs LT	Primary outcomes: all-cause in-hospital and ICU mortality<br>Secondary outcomes: IMV-duration, ICU LOS	No

										all cases using upper respiratory tract samples				
**Deng et al., 2021(75)**	CN	To compare the treatment efficacy of ET vs LT in critically ill patients undergoing MV	RCTs of critically ill patients who received MV with ET as intervention and LT as control; including relevant outcomes	Yes	Interventional	No	15	From inception - 2019	PubMed, Embase, and the Cochrane library	Critically ill patients who received MV	Based on the definition of each individual study	ET vs LT	Short-term mortality, VAP, ICU LOS, MV duration, and hospital LOS	No
**Gomes Silva et al., 2012(76)**	BR	To evaluate the effectiveness and safety of ET versus LT in critically ill adult patients predicted to be on prolonged mechanical ventilation and with different clinical conditions	All RCTs or QRCTs published in any language. Studies published in abstract form were included if sufficient information on their methods and results were provided.<br><br>All outcomes found in the primary studies were considered.	No	interventional	Yes	4	1966 - 2010	CENTRAL, MEDLINE, EMBASE, LILACS, PEDro	Critically ill adult (>= 18 years) patients expected to be on prolonged MV (ventilation for from 24 hours to 21 consecutive days, six or more hours per day)	ET (from 2-10 days after intubation)<br><br>vs<br><br>LT (performed later than 10 days after intubation)	ET vs. LT	Primary outcomes Mortality, Duration of MV<br><br>*Secondary outcomes* ICU LOS, VAP	Yes
**Hosokawa et al., 2015(77)**	BEL	Clarify the potential benefits of early versus late tracheotomy	RCTs that compared outcomes associated with early and late tracheotomy.	Yes	Interventional	No	12	From inception - 2015	PubMed and CENTRAL	Not defined<br><br>Critically ill (ICU setting)	Very early tracheostomy (<4days)<br><br>early tracheostomy (<10days)<br><br>late tracheostomy (>10days)	Very early vs early vs late tracheostomy	MV duration, ICU stay, pneumonia, Mortality	No
**Ji et al., 2022(78)**	CN	To evaluate the impact of ET compared to LT	Studies which compared ET vs. LT	Yes	Observational and	Yes	14	2019 - 2021	Medline, Embase, Cochrane, Scopus,	COVID-19 patients undergoing tracheostomy	ET/LT cut-off ≤ 14 days after	ET vs LT	Primary outcomes: MV duration,	No

		on COVID-19 patients' outcomes	and provided data on at least 1 primary outcome Case reports, reviews, editorials, commentaries, and practice guidelines were excluded		interventional				medRxiv, bioRxiv, and Research Square		initiation of IMV		ICU LOS, overall mortality<br><br>Secondary outcomes: VAP	
**Kishihara et al., 2023(79)**	JP	To perform an NMA of multiple tracheostomy timings using data from RCTs, assess the effect on short-term mortality and adverse events	RCTs comparing tracheostomy timing	Yes	Interventional	Yes	8	From inception - 2023	MEDLINE, CENTRAL, ClinicalTrials.gov, and World Health Organization International Clinical Trials Platform Search Portal	Patients who were 18 years of age or older and mechanically ventilated.	Three groups of tracheostomy timing based on the clinical importance and previous studies: ≤ 4 days, 5–12 days, and ≥ 13 days	Tracheostomy timing	Short-term mortality	
**Liu, X. et al., 2014(80)**	CN	To compare the effect of ET and LT on VAP incidence and short-term mortality	Prospective, randomized, or quasi-randomized trials on tracheotomy in adult patients (older than 15 years) requiring artificial ventilation. The intervention measure was ET compared with LT.	Yes	Observational and interventional	No	11	From inception - 2014	PubMed, Embase, Cochrane Central Register of Controlled Trials, Highwire Press, Springer, CBM, CNKI, Clinical Trials.gov, and the Chinese Clinical Trial Registry	critically ill patients adult patients (older than 15 years) who received mechanical ventilation	NA	ET vs. LT	VAP incidence, short-term mortality	No
**Maziak et al., 1998(81)**	CA	To examine the impact of tracheostomy timing on relevant outcomes of critically ill patients in the ICU	All study designs that examined the impact of tracheostomy at a selected time interval at the ICU on a relevant outcome in mechanically ventilated patients	No	Observational and interventional	No	5	From 1966 - 1996	MEDLINE	Mechanically ventilated patients in the ICU	NA	Tracheostomy timing	MV duration; hospital LOS, VAP	Yes

**Meng et al., 2016(82)**	CN	To compare the outcomes of ET with LT in critically ill patients with prolonged MV	RCTs; where patients were assigned to the ET group or the LT group and that included at least one relevant outcome	Yes	Interventional	No	9	From inception - 2014	PubMed, EMBASE and the Cochrane Library	Critically ill patients with prolonged mechanical ventilation (MV)	ET ≤ 10 days after intubation<br><br>LT > 10 days	ET vs. LT.	Mortality, MV duration, ICU LOS and VAP	No
**Quinn et al., 2022(83)**	UK	Using Bayesian methods to estimate posterior probabilities of the effect of ET on clinical outcomes	RCTs with an intervention group with ET, a comparison group with LT, and one relevant outcome, intention-to-treat analysis	Yes	Interventional	No	19	From inception - 2022	PubMed, EMBASE, and the Cochrane library	ICU patients requiring MV	NA	ET vs LT	Short-term mortality, VAP, ICU LOS, MV duration, and hospital LOS	No
**Shan et al., 2013(84)**	CN	Discuss the potential benefits of ET compared to LT with meta-analysis of observational studies	Prospective or retrospective observational studies, which compared ET (< 7 d) with LT (>= 7 d); written in English	Yes	Observational	No	6	From inception - 2013	MEDLINE, EMBASE, J-STAGE, the Cochrane Library, Global Health, International Pharmaceutical Abstracts, ISI Web of Science, the China National Knowledge Internet, and the grey literature (SIGLE) databases	Critically ill patients who were admitted to ICUs	ET <7d<br>LT ≥ 7d	ET vs LT	Mortality, Mv duration, ICU stay, hospital stay, VAP	No
**Staibano et al., 2021(85)**	CA	To evaluate the association of tracheostomy with COVID-19 patient outcomes and the risk of SARS-CoV-2 transmission among health care	Observational cohort studies, case series (n ≥ 5), and RCTs that reported clinical outcomes in adult patients with COVID-19 who were receiving IMV and identified	Yes	Observational and interventional	Yes	69	2020 - 2021	EMBASE, Medline, and Web of Science	Adult (age, ≥18 years) patients with COVID-19	ET ≤ 14 days<br>LT > 14 d<br>Based on mean or median time from intubation to tracheostomy	ET vs LT	ICU LOS, MV duration	No

		professionals (HCPs).	as candidates for tracheostomy (ST or PDT), studies that evaluated clinical end points selected a priori, studies that reported SARS-CoV-2 transmission among HCPs.										
**Szafran et al., 2023(86)**	GER	To assess the benefits and harms of ET compared to LT in critically ill COVID-19 patients	RCTs and non-randomized studies of interventions (NRSI) evaluating ET compared to LT during SARS-CoV-2 infection	Yes	Observational and interventional	Yes	25	From inception - 2022	Cochrane COVID-19 Study Register, which comprises CENTRAL, PubMed, Embase, ClinicalTrials.gov, WHO International Clinical Trials Registry Platform, and medRxiv, as well as Web of Science and WHO COVID-19 Global literature on coronavirus disease	Adults with a confirmed diagnosis of COVID-19 (as described in the study) that were admitted to the ICU due to respiratory failure, were mechanically ventilated, and underwent surgical or percutaneos tracheostomy	ET ≤ 10days after intubation LT >10 days	ET vs LT	Mortality, need for invasive mechanical ventilation, ventilator-associated pneumonia, ICU/ hospital LOS	No
**Szakmany et al., 2015(87)**	UK	To summarize the available evidence on ET. Specifically, to confirm the effects of ET compared to LT or PI on critical care	RCTs where one of the groups received ET, and the alternative is prolonged tracheal intubation with the potential for a tracheostomy to be placed	Yes	Interventional	Yes	14	1950-2014	CENTRAL; MEDLINE; EMBASE; CINAHL; the NHS Trusts Clinical Trials Register and Current Controlled Trials; LILACS; KoreaMED; MEDCARIB;	Adult critically ill patients expected to require prolonged MV of between 24 h and 21 consecutive days, for more than 6 h per day.	ET ≤ 10d of mechanical ventilation LT/PI >10d	ET vs. LT/PI	Primary outcomes: short-term mortality (within 60d), duration of MV, ICU LOS.<br><br>Secondary outcomes: VAP and	No

		resource utilization and short-term mortality							INDMED; PANTELEIMON; Ingenta; ISI Web of Knowledge and the National Trials Register				mortality at longest follow-up.	
**Cai et al., 2017(88)**	CN	Assess the influence of tracheostomy timing on outcomes among trauma patients.	RCTs or non-RCTs including prospectively observational studies, retrospective cohort studies and case-control studies reporting at least one relevant outcome	Yes	Observational and interventional	No	20	From inception - 2016	PubMed, Web of Science, Cinahl and Cochrane Library	Adult patients with either blunt or penetrating trauma	ET ≤ 7 days after initiation of translaryngeal intubation. LT was any time thereafter	ET vs. LT/PI	Primary outcome: mortality<br><br>Secondary outcomes: Injury Severity Score, Glasgow Coma Scale, hospital or ICU LOS, MV duration and pneumonia incidence	Qual. + Quant Synthesis
**Chorath et al., 2021(89)**	US	To compare the association of ET vs LT with VAP and ventilator days in critically ill adults	RCTs comparing ET and LT with any primary outcomes	Yes	Interventional	Yes	17	From inception - 2020	MEDLINE, CINAHL, Cochrane Central Register of Controlled Trials	Critically ill	ET ≤ 7 days after initiation of mechanical ventilation.<br><br>LT > 7 days or PI	ET vs. LT/PI	Primary outcomes: VAP and MV days.<br><br>Secondary outcomes: short-term mortality, ICU LOS	Yes
**Dunham et al., 2006(90)**	US	Assess outcomes in trauma patients undergoing ET	Retrospective and RCT trauma patient studies with one relevant outcome	Yes	Observational and interventional	No	13	NA	Abstract databases for the Eastern and American Associations for the Surgery of Trauma and Medline	Trauma patients	NA	ET vs. LT/PI	Mortality, pneumonia, MV duration, ICU LOS	Yes
**Griffiths et al., 2005(91)**	UK	To compare outcomes in critically ill patients	RCTs and QRCTs that compared ET with either LT or PI	Yes	Interventional	No	5	From inception - 2004	Medline, CINAHL, Embase, the Cochrane	Critically Ill	ET ≤ 7d after admission to the ICU,	ET vs. LT/PI	The primary outcome: Mortality	No

		undergoing artificial ventilation who received a tracheostomy early or late in their treatment							Central Resister of Clinical Trials, the National Research Register, the NHS Trusts Clinical Trials Register, the Medical Research Council UK database, the NHS Research and Development Health Technology Assessment Programme, and the British Heart Foundation database		initiation of translaryngeal intubation, and mechanical ventilation<br><br>LT was any time thereafter		secondary outcomes: ICU LOS, duration of MV, and incidence of VAP	
**Huang et al., 2014(92)**	CN	To compare important outcomes between ET and LT or PI for critically ill patients receiving long-term ventilation during their treatment.	RCTs of critically ill adult patients who were admitted to ICUs, and required prolonged MV and who were assigned to either an ET group or a LT/PI group, regardless of the tracheotomy technique used. Studies should contain data for at least one of the relevant outcomes.	Yes	Interventional	No	9	From inception - 2013	PubMed, EMBASE and the Cochrane Library	Critically Ill	ET ≤ 10 days after initiation of intubation,<br><br>LT > 10 days	ET vs. LT/PI	Primary outcome: mortality<br><br>Secondary outcomes: duration of MV, length of ICU stay and VAP	No
**Lu et al., 2018(93)**	CN	To clarify whether ET can also improve	RCTs, prospective observational studies, or retrospective	Yes	Observational and	No	8	1995 - 2017	PubMed, Web of Science, Elsevier	Severe TBI (Glasgow Coma Scale score	ET <10 days	ET vs. LT/PI	ICU / hospital LOS, MV duration,	No

		outcomes in patients with severe TBI	cohort studies with isolated severe TBI patients, comparing: ET versus LT/PI on relevant outcomes		interven tional				ScienceDirect, and Cochrane Library	<=8)			mortality, VAP	
**Siempos et al., 2015(94)**	US	To assess the benefit of early versus late or no tracheostomy on mortality and pneumonia in critically ill patients who need mechanical ventilation	RCTs and quasi-randomised con trolled trials that compared ETwith late or no tracheostomy in critically ill patients receiving mechanical ventilation and reported all-cause mortality or incidence of pneumonia as eligible for inclusion. patients receiving prolonged translaryngeal intubation	Yes	Interven tional	Yes	16	From inception - 2013	PubMed, CINAHL, Embase, Web of Science, Directory of Open Access Journals, and the Cochrane Central Register of Controlled Trials	Critically Ill	ET = done during the first week after translaryng eal intubation.<br><br>LT= as being done any time after the first week of MV	ET vs. LT/PI	All-cause mortality or incidence of pneumonia	No
**Villemure-Poliquin et al., 2020(95)**	CA	To evaluate the effect of ET vs. LT/PI in nonneurologi cally injured mechanically ventilated critically ill adult patients	RCTs comparing ET to LT or PI In non-neurologically adults (at least 75%). On at least one relevant outcome	Yes	Interven tional	Yes	9	From inception - 2022	MEDLINE, EMBASE, Web of Science, CINAHL, and CENTRAL	Non-neurologicall y injured mechanically ventilated critically ill adult patients	ET (< 10 d after intubation)<br><br>LT (≥ 10 days after intubation)	ET vs. LT/PI	Primary outcome: all-cause mortality<br><br>Secondary outcomes: ICU / hospital LOS, MV duration, VAP	Yes
**Wang et al., 2011(96)**	CN	To systematically review and quantitatively synthesize all RCTs, comparing important	RCTs that compared ET with either LT or PI in critically ill adult patients	Yes	Interven tional	No	7	From inception - 2011	PubMed, CINAHL, Embase, the Cochrane Central Register of Controlled Trials, the National	Critically ill adult patients	ET ≤ 7d after initiation of translaryng eal intubation and MV	ET vs. LT/PI	Primary outcomes: short-term mortality and VAP incidence	

		outcomes in ventilated critically ill patients who received ET or LT							Research Register, the National Health Service Trusts Clinical Trials Register, and the Medical Research Council UK database		LT = anytime thereafter		Secondary outcomes: long-term mortality, MV duration, ICU and hospital LOS	

AIS = acute ischemic stroke; BEL = Belgium; BR = Brazil; CA = Canada; CENTRAL = Cochrane Central Register of Controlled Trials; CN = China; COVID-19 = coronavirus disease 2019; EG = Egypt; ET = early tracheostomy; GCS = Glasgow Coma Scale; GER = Germany; HIE = hypoxic-ischemic encephalopathy; ICH = intracerebral hemorrhage; ICU = intensive care unit; IMV = invasive mechanical ventilation; IT = Italy; JP = Japan; LOS = length of stay; LT = late tracheostomy; MV = mechanical ventilation; NA = not applicable; NMA = network meta-analysis; PDT = percutaneous dilatational tracheostomy; PI = prolonged intubation; QRCT = quasi-randomized controlled trial; RCT = randomized controlled trial; RQ = research question; SABI = severe acute brain injury; SARS-CoV-2 = severe acute respiratory syndrome coronavirus 2; SCI = spinal cord injury; SGP = Singapore; ST = surgical tracheostomy; TBI = traumatic brain injury; UK = United Kingdom; US = United States; USA = United States; VAP = ventilator-associated pneumonia.

Supplement Table S4 Critical appraisal of included systematic reviews and meta-analyses in synthesis based on AMSTAR-2 domains

Author, publication year	Q1	Q2	Q3	Q4	Q5	Q6	Q7	Q8	Q9[†]	Q10	Q11	Q12	Q13	Q14	Q15	Q16	Overall assessment
Chorath, 2021	y	y	n[1]	y	y	y	y	n[2]	y	n[3]	y	y	y	y	y	y	moderate
Kishihara, 2023	y	y	n[1]	y	y	y	n[2]	n[3]	y	n[4]	y	y	n[5]	y	n[6]	y	critically low
Quinn, 2022	y	n[1]	n[2]	y	n[3]	n[4]	y	n[5]	n[6]	n[7]	y	n[8]	n[9]	y	n[10]	y	critically low
Qiu, 2023	y	y	n[1]	y	n[2]	n[3]	y	y	y	n[4]	y	n[5]	n[6]	y	y	y	critically low
Bertini, 2023	y	y	n[1]	y	n[2]	n[3]	y	n[4]	y	n[5]	y	y	y	y	y	y	moderate
Foran, 2022	y	y	n[1]	y	n[2]	y	n[3]	y	y	n[4]	y	y	n[5]	y	n[6]	y	critically low
Szafran, 2023	y	y	y	y	y	y	y	y	y	y	y	y	y	y	y	y	high
Cai, 2017	y	n[1]	n[2]	y	n[3]	n[4]	y	y	y	n[5]	n[6]	y	n[7]	n[8]	y	y	critically low
Villemure-Poliquin, 2020	y	y	n[1]	y	n[2]	y	y	n[3]	y	y	y	y	n[4]	y	y	y	low

AMSTAR-2 overall assessment rating: high—the review provides an accurate and comprehensive summary of the results of the available studies that addresses the question of interest; moderate—the review has more than one weakness, but no critical flaws. It may provide an accurate summary of the results of the available studies; low—the review has a critical flaw and may not provide an accurate and comprehensive summary of the available studies that address the question of interest; or critically low—the review has more than one critical flaw and should not be relied on to provide an accurate and comprehensive summary of the available studies.

n = no; na = not applicable; Q = Question; y = yes.

Q1: Did the research questions and inclusion criteria for the review include the components of PICO?.

Q2: Did the report of the review contain an explicit statement that the review methods were established prior to the conduct of the review and did the report justify any significant deviations from the protocol?.

Q3: Did the review authors explain their selection of the study designs for inclusion in the review?.

Q4: Did the review authors use a comprehensive literature search strategy?.

Q5: Did the review authors perform study selection in duplicate?.

Q6: Did the review authors perform data extraction in duplicate?.

Q7: Did the review authors provide a list of excluded studies and justify the exclusions?.

Q8: Did the review authors describe the included studies in adequate detail?.

Q9: Did the review authors use a satisfactory technique for assessing the risk of bias (RoB) in individual studies that were included in the review?.

Q10: Did the review authors report on the sources of funding for the studies included in the review?.

Q11: If meta-analysis was performed did the review authors use appropriate methods for statistical combination of results?.

Q12: If meta-analysis was performed, did the review authors assess the potential impact of RoB in individual studies on the results of the meta-analysis or other evidence synthesis?.

Q13: Did the review authors account for RoB(only for RCTs -> or other suitable tool) in individual studies when interpreting/discussing the results of the review?.

Q14: Did the review authors provide a satisfactory explanation for, and discussion of, any heterogeneity observed in the results of the review?

Q15: If they performed quantitative synthesis did the review authors carry out an adequate investigation of publication bias (small study bias) and discuss its likely impact on the results of the review?.

Q16: Did the review authors report any potential sources of conflict of interest, including any funding they received for conducting the review?.

Chorath, 2021:
[1]no justification for including only RCTs
[2]no outcomes described
[3]no funding of included studies reported

Kishihara, 2023:
[1]no justification of including only RCTs
[2]no list of excluded studies
[3]can't find/access online supplement, they mention characteristics of included studies are described in e-Table 4
[4]can't find/access online supplement, maybe in online supplement
[5]no mentioning of RoB in the discussion/interpretation of results
[6]<10 studies -> did not test for funnel asymmetry, but did not discuss potential impact of publication bias on the results

Quinn, 2022:
[1]protocol not mentioned, needed for this Bayesian analysis?
[2]no justification for including only RCTs
[3]not mentioned that study selection was done in duplicate
[4]ot mentioned that data extraction was done in duplicate
[5]outcomes not described
[6]no RoB assessed
[7]did not report on sources of funding of included studies
[8]no RoB
[9]no RoB
[10]No investigation of publication bias

Qiu, 2023:
[1]no justification for included study design
[2]full-text not screened in duplicate
[3]data-extraction not in dplicate
[4]funding of included studies not reported
[5]no RoB
[6]no RoB

Bertini, 2023:
[1]no justification for included study design
[2]only abstract screening in duplicate
[3] nothing mentioned that data extraction was done in duplicate
[4] outcomes not described
[5] did not report on funding of included studies

Foran, 2022:
[1]no justification for included study design
[2]title and abstract screening not in duplicate
[3]no list of reasons for exclusion
[4]did not report on funding of included studies
[5]did not take into account Newcastle Ottawa Scale (NOS) when interpreting/discussing results of the review
[6]no assessment of publication bias

Szafran, 2023:
-

Cai, 2017:
[1] no protocol
[2]no justification for included study design
[3] full text screening not in duplicate
[4] data extraction not in duplicate
[5] sources of funding of included studies not reported
[6] pooled RCTs and observational studies without separate effect estimates
[7] no account for RoB when interpreting/discussing the results of the review
[8] heterogeneity not sufficiently explained

Villemure-Poliquin, 2020
[1]no justification for included study design
[2] full-text screening not in duplicate
[3] population and outcomes not described
[4] no discussion of RoB when interpreting the findings

Supplement Table S5 Risk of bias assessment of primary randomized controlled trials identified within the included systematic reviews using the Cochrane risk of bias tool (RoB 1)

**Source Systematic Reviews**	**Study, year**	**Random sequence generation**	**Allocation concealment**	**Performance bias**	**Detection bias**	**Attrition bias**	**Selective reporting**	**Other bias**
Cai, 2017	Barquist, 2006	Low	Low	Low	Low	Low	Low	Low
Chorath, 2021; Villemure-Poliquin, 2020	Blot, 2008	Low	Low	High	Unclear	Low	Low	Low
Chorath, 2021; Qiu, 2023	Bösel, 2013	Low	Unclear	Low	Unclear	Low	Unclear	Unclear
Qiu, 2023	Bösel, 2022	Low	Low	High	Low	Low	Unclear	Unclear
Chorath, 2021; Cai 2017	Bouderka, 2004	Unclear	Unclear	Unclear	Unclear	Low	Low	High
Chorath, 2021	Bylappa, 2011	Unclear	Unclear	Low	Unclear	Low	Low	High
Chorath, 2021; Villemure-Poliquin, 2020	Diaz-Prieto, 2014	Low	High	High	Unclear	Low	Low	High
Chorath, 2021; Cai, 2017	Dunham, 2014	High	Unclear	Low	Unclear	Unclear	Unclear	High
Szafran, 2023	Eeg-Olofsson, 2022	Unclear	Low	Low	Unclear	Low	Low	Low
Chorath, 2021	Filaire, 2015	Unclear	Low	Low	Unclear	Low	Low	Low
Quinn, 2022	Goo, 2022	Unclear	Low	High	Unclear	Low	Low	Low
Chorath, 2021	Karlović, 2018	Unclear	Low	Low	Unclear	Low	Low	Low
Chorath, 2021	Koch, 2012	Low	Low	Low	Low	Low	Low	Low
Quinn, 2022	Mohammed, 2014	Unclear	Unclear	High	High	Low	Unclear	Low
Chorath, 2021; Cai, 2017	Rodriguez, 2004	High	High	Unclear	Unclear	Low	Unclear	Low
Bertini, 2023	Roushdy, 2018	High	High	High	High	Unclear	Low	High
Chorath, 2021; Villemure-Poliquin, 2020	Rumbak, 2004	Unclear	Low	High	Unclear	Low	High	High

Chorath, 2021; Villemure-Poliquin, 2020	Saffle, 2002	Low	Low	High	Unclear	Low	Low	Low
Kishihara, 2023	Shekhar, 2022	Unclear	Unclear	High	Unclear	High	Low	Low
Chorath, 2021; Cai, 2017; Villemure-Poliquin, 2020	Sugerman, 1997	High	High	High	Unclear	High	Unclear	Unclear
Chorath, 2021; Villemure-Poliquin, 2020	Terragni, 2010	Low	Low	High	Low	Low	Low	Low
Chorath, 2021; Villemure-Poliquin, 2020	Trouillet, 2011	Low	Low	High	Unclear	Low	Low	Low
Chorath, 2021; Villemure-Poliquin, 2020	Young, 2013	Low	Low	High	Low	Low	Low	Low
Chorath, 2021; Villemure-Poliquin, 2020	Zheng, 2012	Low	Low	High	Unclear	Low	Low	Low

Supplement Table S6 Risk of bias assessment of primary non-randomized studies identified within the included systematic reviews using the Newcastle-Ottawa Scale (NOS)

Source Reviews	Study	Risk	Selection	Comparability	Outcome / Exposure	NOS-score
Foran, 2022	Babu, 2013	High	***	*	**	6
Foran, 2022	Choi, 2013	High	***	**	*	6
Foran, 2022	Flanagan, 2018	Low	***	**	**	7
Foran, 2022	Galeiras, 2018	Low	****	**	**	8
Foran, 2022	Ganuza, 2011	Low	****	**	**	8
Foran, 2022	Guirgis, 2016	Low	****	*	**	7
Foran, 2022	Holscher, 2014	High	***	0	**	5
Foran, 2022	Jeon, 2015	Low	****	**	**	8
Foran, 2022	Khan, 2020	Low	****	**	**	8
Foran, 2022	Kornblith, 2013	Low	****	*	**	7
Foran, 2022	Lozano, 2018	Low	****	*	**	7
Foran, 2022	Romero, 2009	Low	****	**	**	8
Foran, 2022	Vitaz, 2001	High	***	0	**	5
Foran, 2022	Wu, 2013	High	NA[a]			
Qiu, 2023	Chen, 2019	Low	****	**	**	8
Qiu, 2023	Alsherbini, 2014	Low	****	**	***	9
Qiu, 2023	Hallan, 2022	Low	****	**	***	9
Qiu, 2023	Gessler, 2015	Low	****	**	***	9
Qiu, 2023	Elsayed, 2022	Low	****	**	**	8
Qiu, 2023	Shen, 2022	Low	***	**	***	8
Cai, 2017	Kluger, 1996	High	***	0	***	7
Cai, 2017, Szafran, 2023	Armstrong, 1998	High	***	0	***	6
Cai, 2017, Szafran, 2023	Arabi, 2004	Low	****	**	***	9
Cai, 2017	Hyde, 2015	Low	***	**	**	7
Cai, 2017	Lesnik, 1992	High	NA[b]			
Cai, 2017	Chintamani, 2005	Low	***	**	***	
Cai, 2017	Pinheiro, 2010	High	***	0	**	5
Bertini, 2023	Ahmed, 2007	High	****	0	***	7

Bertini, 2023	Wang, 2012	High	****	0	***	7
Bertini, 2023	Huang, 2013	High	****	0	***	7
Bertini, 2023	Alali, 2014	Low	****	**	***	9
Bertini, 2023	Siddiqui, 2015	High	****	0	***	7
Bertini, 2023	Shibahashi, 2017	High	****	0	***	7
Bertini, 2023	Khalili, 2017	High	****	0	***	7
Bertini, 2023	Lu, 2019	High	****	0	***	7
Bertini, 2023	McLaughlin, 2019	Low	****	**	***	9
Bertini, 2023	Sheehan, 2019	High	****	0	***	7
Bertini, 2023	Robba, 2020	Low	****	**	***	9
Bertini, 2023	Ismail, 2021	High	****	0	***	7
Szafran, 2023	Livneh 2021	High	****	0	***	7
Szafran, 2023	Avilés-Jurado, 2020	High	****	0	***	7
Szafran, 2023	Glibbery 2020	Low	***	**	**	7
Szafran, 2023	Takhar 2020	High	****	0	***	7
Szafran, 2023	Tetaj 2021	Low	****	**	**	8
Szafran, 2023	Arnold 2022	High	****	0	**	6
Szafran, 2023	Battaglini 2021	Low	****	*	**	7
Szafran, 2023	Hernandez 2022	Low	****	**	**	8
Szafran, 2023	Mahmood 2021	High	***	0	**	5
Szafran, 2023	Tsonas 2022	Low	****	**	**	8
Szafran, 2023	Prats-Uribe 2021	Low	****	**	***	9
Szafran, 2023	Volo 2021	Low	****	**	**	8
Szafran, 2023	Angel 2021	Low	****	**	**	8
Szafran, 2023	Dal 2022	Low	****	*	***	8
Szafran, 2023	Kuno 2021	Low	****	*	***	8

[a] Full text available in Mandarin only. We conservatively assigned the study to be at high risk of bias, because formal assessment was not possible.
[b] Document not available in electronic format and physical copy could not be retrieved in time for analysis. We conservatively assigned the study to be at high risk of bias, because formal assessment was not possible.

Supplement Table S7 Estimates for early versus late tracheostomy stratified by clinical diagnosis: evidence from observational studies

Population	Outcome	No. of studies	Effect estimate (95% CI)	*P*	I^2	P_{het}	tau^2	*Publication bias* (P_{Egger})[a]
Traumatic Brain Injury	MV Duration (days)	8	MD -6.06 (-8.57 to -3.54)	**< 0.001**	95.76%	< 0.001	9.71	-
	ICU LOS (days)	11	MD -7.16 (-8.85 to -5.47)	**< 0.001**	85.62%	< 0.001	4.88	0.05
	Hospital LOS (days)	10	MD -7.31 (-9.05 to -5.57)	**< 0.001**	43.83%	0.07	2.39	0.18
	VAP	10[b]	OR 0.55 (0.44 to 0.68)	**< 0.001**	17.14%	0.29	0.02	0.06
	Mortality	12[c]	OR 1.32 (0.91 to 1.90)	0.14	41.29%	0.07	0.12	0.05
Stroke	MV Duration (days)	4	MD -6.19 (-9.39 to -2.99)	**< 0.001**[d]	76.17%	0.01	7.92	-
	ICU LOS (days)	4	MD -6.35 (-8.10 to -4.59)	**< 0.001**	80.47%	< 0.001	1.86	-
	Hospital LOS (days)	2	MD -7.81 (-10.46 to -5.17)	**< 0.001**	0.00%	0.98	0.00	-
	VAP	3	OR 1.13 (0.97 to 1.31)	0.11	0.00%	0.80	0.00	-
	Mortality	4	OR 0.97 (0.79 to 1.18)	0.73	0.00%	0.50	0.00	-
Trauma	MV Duration (days)	7	MD -12.38 (-19.75 to -5.02)	**< 0.001**	95.35%	< 0.001	87.87	-
	ICU LOS (days)	7[e]	MD -11.24 (-15.75 to -6.72)	**< 0.001**	97.99%	< 0.001	29.74	-
	Hospital LOS (days)	4	MD -13.56 (-29.46 to 2.33)	0.09	85.04%	< 0.001	195.55	-
	VAP	9[f]	OR 0.37 (0.23 to 0.61)	**< 0.001**	58.20%	0.01	0.27	-
	Mortality	9	OR 0.97 (0.63 to 1.47)	0.87	25.34%	0.22	0.10	-
COVID-19	MV Duration (days)	4	MD -0.44 (-5.03 to 4.15)	0.85	65.59%	0.03	12.35	-
	ICU LOS (days)	4	MD -7.25 (-13.19 to -1.30)	**0.02**[g]	65.54%	0.03	23.52	-
	Hospital LOS (days)	2	MD 1.89 (-11.30 to 15.07)	0.78	84.34%	0.01	76.86	-
	VAP	4	OR 0.65 (0.50 to 0.86)	**< 0.001**	0.00%	0.74	0.00	-
	Mortality	10	OR 1.15 (0.76 to 1.74)	0.52	42.70%	0.07	0.17	0.42
Spinal Cord Injury	MV Duration (days)	8	MD -13.96 (-20.68 to -7.24)	**< 0.001**	94.20%	< 0.001	72.50	-
	ICU LOS (days)	8	MD -10.52 (-16.19 to -4.85)	**< 0.001**	89.07%	< 0.001	29.56	-
	Hospital LOS (days)	6	MD -8.17 (-12.51 to -3.83)	**< 0.001**	0.00%	0.43	51.02	-
	VAP	10	OR 0.64 (0.49 to 0.83)	**< 0.001**	0.00%	0.58	0.00	0.38
	Mortality[g]	10	OR 0.83 (0.36 to 1.91)	0.66	54.81%	0.02	0.88	0.93

[a] Publication bias was assessed through funnel plot symmetry (Supplement Figures 13-19) and Egger's regression test, with $P < 0.10$ indicating significant asymmetry (small-study effects).
[b, c] The study by Elkbuli (2019) was removed from this analysis due to an Expression of concern.
[d] This parameter was no longer statistically significant (p = 0.05) in a sensitivity analysis using a random-effects model with restricted maximum likelihood (REML) estimation and Knapp-Hartung standard error adjustments.
[e] The study by D'Amelio (1994) was removed from this analysis as measures of dispersion could not be obtained
[f] The study by Rizk (2011) was removed from this analysis as the outcome was a composite measure of pulmonary occurrence.
[g] This parameter was no longer statistically significant (p = 0.08) in a sensitivity analysis using a random-effects model with restricted maximum likelihood (REML) estimation and Knapp-Hartung standard error adjustments.
[g] short-term mortality
CI = confidence interval; ICU = intensive care unit; LOS = length of stay; MD = mean difference; MV = mechanical ventilation; OR = odds ratio; P_{Egger} = p-value for Egger's test of publication bias; P_{het} = p-value for heterogeneity; RCT = randomized controlled trial; VAP = ventilator-associated pneumonia.

Supplement Table S8 Pooled effect estimates for early (≤7 days) versus late (>7 days) or no tracheostomy in patients with heterogeneous causes of critical illness

Outcome	No. of studies	Effect estimate (95%CI)	*P*	I^2	P_{het}	tau²	Publication bias (P_{Egger})[a]
**Evidence from RCTs**							
MV Duration	13	MD -3.41 (-5.94 to -0.89)	**0.01**[b]	91.9%	< 0.001	17.43	0.18
ICU LOS	11	MD -6.31 (-9.85 to -2.78)	**< 0.001**	96.9%	< 0.001	27.81	0.03
Hospital LOS	2	MD -8.72 (-22.95 to 5.52)	0.23	89.2%	0.02	89.16	-
VAP	15	OR 0.60 (0.41 to 0.88)	**0.01**	60.5%	< 0.001	0.29	0.13
Mortality	17	OR 0.82 (0.65 to 1.03)	0.09	41.2%	0.04	0.08	0.95
**Evidence from non-RCTs**							
MV Duration	11	MD -7.05 (-9.27 to -4.83)	**< 0.001**	75.4%	< 0.001	8.86	0.71
ICU LOS	12	MD -8.23 (-10.34 to -6.13)	**< 0.001**	94.0%	< 0.001	9.35	0.99
Hospital LOS	7	MD -9.51 (-13.69 to -5.33)	**< 0.001**	82.0%	< 0.001	23.27	-
VAP	10[c, d]	OR 0.73 (0.54 to 1.00)	0.05	67.7%	< 0.001	0.14	0.92
Mortality	13[e]	OR 0.82 (0.57 to 1.16)	0.25	30.8%	0.14	0.10	0.32

[a] Publication bias was assessed through funnel plot symmetry and Egger's regression test, with $P < 0.10$ indicating significant asymmetry (small-study effects).
[b] This parameter was no longer statistically significant (p = 0.050) in a sensitivity analysis using a random-effects model with restricted maximum likelihood (REML) estimation and Knapp-Hartung standard error adjustments.
[c, e] The study by Elkbuli (2019) was removed from this analysis due to an Expression of concern.
[d] The study by Rizk (2011) was removed from this analysis as the outcome was a composite measure of pulmonary occurrence.
CI = confidence interval; GRADE = Grading of Recommendations Assessment, Development and Evaluation; I^2 = inconsistency (heterogeneity) index; ICU = intensive care unit; LOS = length of stay; MD = mean difference; MV = mechanical ventilation; OR = odds ratio; P_{Egger} = p-value for Egger's test of publication bias; P_{het} = p-value for heterogeneity; RCT = randomized controlled trial; tau^2 = between-study variance; VAP = ventilator-associated pneumonia.

Supplement Table S9 Certainty of evidence assessment for early versus late tracheostomy using the GRADE framework

No. of systematic reviews	No. of original studies	Design	Risk of bias	Inconsistency	Indirectness[a]	Imprecision[b]	Other considerations (publication bias)	No. of individuals in early tracheostomy group	No. of individuals in late tracheostomy group	Association estimate	Quality	Importance
**Mechanical ventilation duration (various populations)**												
7	16	RCTs	None	Serious[c]	None	None	None	1123	1110	MD -3.76 (-6.01 to -1.52)	Moderate	Very important
5	27	Observational	Serious[d]	None	None	None	None	1743	2259	MD -8.19 (-11.52 to -4.85)	Very low	Very important
**ICU Length of stay (various populations)**												
6	13	RCTs	Serious[e]	None	None	None	Serious[f]	1386	1399	MD -6.64 (-9.92 to -3.35)	Low	Very important
5	30	Observational	Serious[g]	None	None	None	None	3104	3649	MD -8.59 (-10.54 to -6.63)	Very low	Very important
**Hospital Length of stay (various populations)**												
2	2	RCTs	Very serious[h]	Serious[i]	None	Serious[j]	None	237	249	MD -8.72 (-22.95 to 5.52)	Very low	Very important
5	22	Observational	Serious[k]	Serious[l]	None	None	Serious[m]	1699	2009	MD -7.15 (-9.32 to -4.98)	Very low	Very Important
**Ventilator-associated pneumonia (various populations)**												
5	19	RCTs	Serious[n]	None	None	None	None	1185	1195	OR 0.66 (0.46 to 0.94)	Moderate	Very important
5	31	Observational	Serious[o]	None	None	None	None	4009	4988	OR 0.58 (0.47 to 0.71)	Very low	Very important
**Mortality (various populations)**												
7	22	RCTs	Serious[p]	Serious[q]	None	None	None	1892	1888	OR 0.84 (0.68 to 1.05)	Low	Important
5	42	Observational	Serious[r]	None	None	None	None	6200	7727	OR 1.03 (0.81 to 1.30)	Very low	Important

GRADE = Grading of Recommendations Assessment, Development and Evaluation; ICU = intensive care unit

[a] We decided not to downgrade for indirectness for the following reasons: population is overall representative of population who requires tracheostomy, outcomes are standardized (e.g., mortality, VAP, LOS, and ventilation duration) and definition of early and late tracheostomy varied across studies, but also there is no an official cut-off to define early vs late tracheostomy

[b] Considering the associated costs, burden to the patient and potential complications with prolonged ventilation, ICU and hospital length of stay, difference in a single day between the two groups has been considered clinically relevant. In case of binary outcomes (mortality and VAP), OR≥1.10 or ≤0.90 has been considered as clinically relevant. Sample size of ≥2000 individuals in observational studies has been considered as sufficient., and 600 in case or RCTs.

[c] 95% Cis across the studies partially overlap. The overall association estimate is significant but heterogeneity is very high (90.54%), which is not explained by subgroup analysis (p =0.54).

[d] We downgraded once as 48% of included studies were at moderate-to-high risk, mainly due to issues in comparability (inadequate adjustment for key confounders) on the Newcastle-Ottawa Scale.

[e] We downgraded once, as included studies were at high risk of performance bias with a potential for detection bias.

[f] We downgraded once due to funnel plot asymmetry and a significant Egger's test (p = 0.04).

[g] We downgraded once as 55% of included studies were at moderate-to-high risk of bias, mainly due to issues in comparability (inadequate adjustment for key confounders) on the Newcastle-Ottawa Scale.

[h] We downgraded twice for very serious risk of bias due to concerns with random sequence generation, allocation concealment, and blinding of participants/personnel across the included studies.

[i] 95% Cis across the studies partially overlap. The overall association estimate is non-significant and heterogeneity is very high (83.09%). However, subgroup analysis per disease group has shown that those with Trauma and early tracheostomy had a 17 days (-28.12 to -5.88) shorter hospital LOS in comparison to late tracheostomy.

[j] Downgraded once for small sample size (n=486).

[k] We downgraded once as 64% of included studies were at moderate-to-high risk of bias, mainly due to issues in comparability (inadequate adjustment for key confounders) on the Newcastle-Ottawa Scale.

[l] 95% Cis across the studies partially overlap. The overall association estimate is significant but heterogeneity is high (71.45%), which is not explained adequately by subgroup analysis (p =0.61).

[m] We downgraded once due to funnel plot asymmetry and a significant Egger's test (p = 0.05).

[n] We downgraded once, as included studies were at high risk of performance bias with a potential for detection bias.

[o] We downgraded once as 65% of included studies were at moderate-to-high risk of bias, mainly due to issues in comparability (inadequate adjustment for key confounders) on the Newcastle-Ottawa Scale.

[p] We downgraded once, as included studies were at high risk of performance bias with a potential for detection bias.

[q] 95% Cis across the studies partially overlap. The overall association estimate is non-significant and heterogeneity is moderate. However, subgroup analysis per disease group has shown that Critically Ill with early tracheostomy had 30% lower odds of dying in comparison with late tracheostomy.
[r] We downgraded once as 58% of included studies were at moderate-to-high risk of bias, mainly due to issues in comparability (inadequate adjustment for key confounders) on the Newcastle-Ottawa Scale.
Note: Since we re-pooled original studies, we have treated each outcome as a de novo meta-analysis and applied standard GRADE to that pooled evidence. For original studies not assessed by the included systematic reviews or for which primary reports were unavailable, a high risk of bias was conservatively assumed to avoid overestimating the certainty of the evidence.

Supplement Table S10 PRISMA 2020 Checklist

Section and Topic	Item #	Checklist item	Location where item is reported
**TITLE**			
Title	1	Identify the report as a systematic review.	1
**ABSTRACT**			
Abstract	2	See the PRISMA 2020 for Abstracts checklist.	1
**INTRODUCTION**			
Rationale	3	Describe the rationale for the review in the context of existing knowledge.	2
Objectives	4	Provide an explicit statement of the objective(s) or question(s) the review addresses.	2
**METHODS**			
Eligibility criteria	5	Specify the inclusion and exclusion criteria for the review and how studies were grouped for the syntheses.	6-7
Information sources	6	Specify all databases, registers, websites, organisations, reference lists and other sources searched or consulted to identify studies. Specify the date when each source was last searched or consulted.	6
Search strategy	7	Present the full search strategies for all databases, registers and websites, including any filters and limits used.	6
Selection process	8	Specify the methods used to decide whether a study met the inclusion criteria of the review, including how many reviewers screened each record and each report retrieved, whether they worked independently, and if applicable, details of automation tools used in the process.	6-7
Data collection process	9	Specify the methods used to collect data from reports, including how many reviewers collected data from each report, whether they worked independently, any processes for obtaining or confirming data from study investigators, and if applicable, details of automation tools used in the process.	7-8
Data items	10a	List and define all outcomes for which data were sought. Specify whether all results that were compatible with each outcome domain in each study were sought (e.g. for all measures, time points, analyses), and if not, the methods used to decide which results to collect.	6
	10b	List and define all other variables for which data were sought (e.g. participant and intervention characteristics, funding sources). Describe any assumptions made about any missing or unclear information.	6
Study risk of bias assessment	11	Specify the methods used to assess risk of bias in the included studies, including details of the tool(s) used, how many reviewers assessed each study and whether they worked independently, and if applicable, details of automation tools used in the process.	8
Effect measures	12	Specify for each outcome the effect measure(s) (e.g. risk ratio, mean difference) used in the synthesis or presentation of results.	7-8
Synthesis	13a	Describe the processes used to decide which studies were eligible for each synthesis (e.g. tabulating the study intervention characteristics and	8-9

Section and Topic	Item #	Checklist item	Location where item is reported
methods		comparing against the planned groups for each synthesis (item #5)).	
	13b	Describe any methods required to prepare the data for presentation or synthesis, such as handling of missing summary statistics, or data conversions.	8-9
	13c	Describe any methods used to tabulate or visually display results of individual studies and syntheses.	8-9
	13d	Describe any methods used to synthesize results and provide a rationale for the choice(s). If meta-analysis was performed, describe the model(s), method(s) to identify the presence and extent of statistical heterogeneity, and software package(s) used.	8-9
	13e	Describe any methods used to explore possible causes of heterogeneity among study results (e.g. subgroup analysis, meta-regression).	8-9
	13f	Describe any sensitivity analyses conducted to assess robustness of the synthesized results.	8-9
Reporting bias assessment	14	Describe any methods used to assess risk of bias due to missing results in a synthesis (arising from reporting biases).	10
Certainty assessment	15	Describe any methods used to assess certainty (or confidence) in the body of evidence for an outcome.	10
**RESULTS**			
Study selection	16a	Describe the results of the search and selection process, from the number of records identified in the search to the number of studies included in the review, ideally using a flow diagram.	10, Figure 1
	16b	Cite studies that might appear to meet the inclusion criteria, but which were excluded, and explain why they were excluded.	Supplement Table S2
Study characteristics	17	Cite each included study and present its characteristics.	Table 1, Supplement Table S3
Risk of bias in studies	18	Present assessments of risk of bias for each included study.	14, Supplement Tables S4&S5
Results of individual studies	19	For all outcomes, present, for each study: (a) summary statistics for each group (where appropriate) and (b) an effect estimate and its precision (e.g. confidence/credible interval), ideally using structured tables or plots.	16
Results of syntheses	20a	For each synthesis, briefly summarise the characteristics and risk of bias among contributing studies.	15
	20b	Present results of all statistical syntheses conducted. If meta-analysis was done, present for each the summary estimate and its precision (e.g. confidence/credible interval) and measures of statistical heterogeneity. If comparing groups, describe the direction of the effect.	15
	20c	Present results of all investigations of possible causes of heterogeneity among study results.	16-19
	20d	Present results of all sensitivity analyses conducted to assess the robustness of the synthesized results.	16-19
Reporting biases	21	Present assessments of risk of bias due to missing results (arising from reporting biases) for each synthesis assessed.	19-20

Section and Topic	Item #	Checklist item	Location where item is reported
Certainty of evidence	22	Present assessments of certainty (or confidence) in the body of evidence for each outcome assessed.	20
**DISCUSSION**			
Discussion	23a	Provide a general interpretation of the results in the context of other evidence.	21-23
	23b	Discuss any limitations of the evidence included in the review.	23-24
	23c	Discuss any limitations of the review processes used.	23-24
	23d	Discuss implications of the results for practice, policy, and future research.	23-24
**OTHER INFORMATION**			
Registration and protocol	24a	Provide registration information for the review, including register name and registration number, or state that the review was not registered.	1
	24b	Indicate where the review protocol can be accessed, or state that a protocol was not prepared.	1
	24c	Describe and explain any amendments to information provided at registration or in the protocol.	None
Support	25	Describe sources of financial or non-financial support for the review, and the role of the funders or sponsors in the review.	10, 26
Competing interests	26	Declare any competing interests of review authors.	26
Availability of data, code and other materials	27	Report which of the following are publicly available and where they can be found: template data collection forms; data extracted from included studies; data used for all analyses; analytic code; any other materials used in the review.	26

From: Page MJ, McKenzie JE, Bossuyt PM, Boutron I, Hoffmann TC, Mulrow CD, et al. The PRISMA 2020 statement: an updated guideline for reporting systematic reviews. BMJ 2021;372:n71. doi: 10.1136/bmj.n71

For more information, visit: http://www.prisma-statement.org/

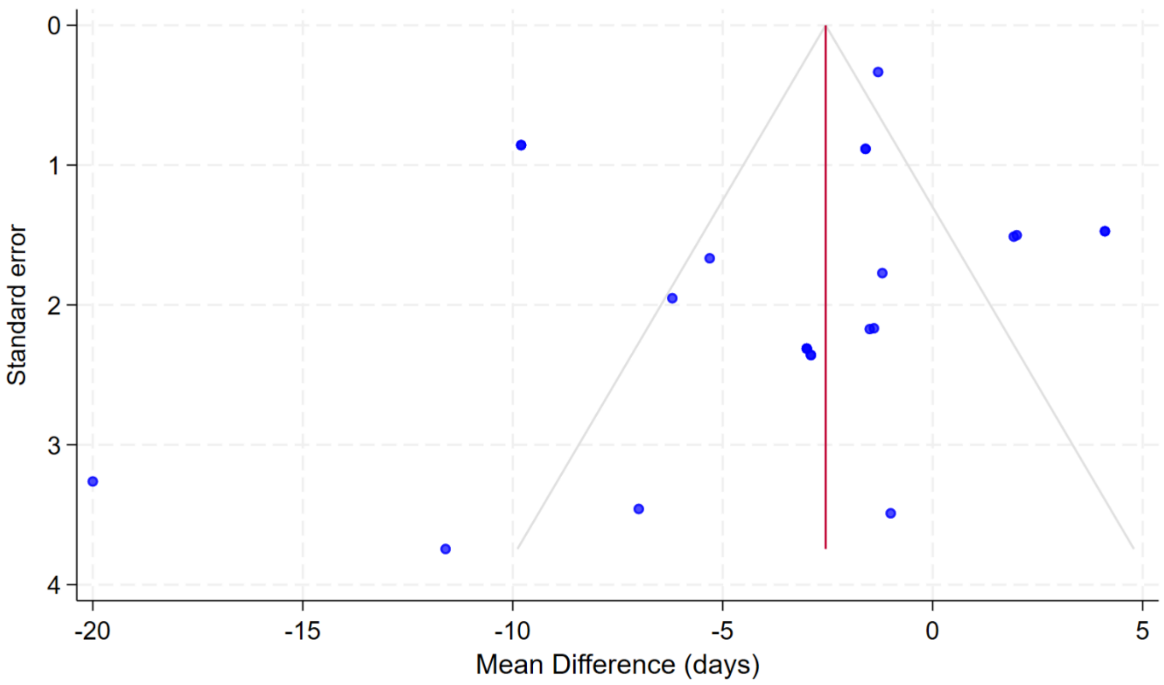

Note. Negative mean difference values favor early tracheostomy (shorter duration of mechanical ventilation); positive values favor late tracheostomy.

Supplement Figure S1 Funnel plot of standard error by mean difference for mechanical ventilation duration (evidence from RCTs)

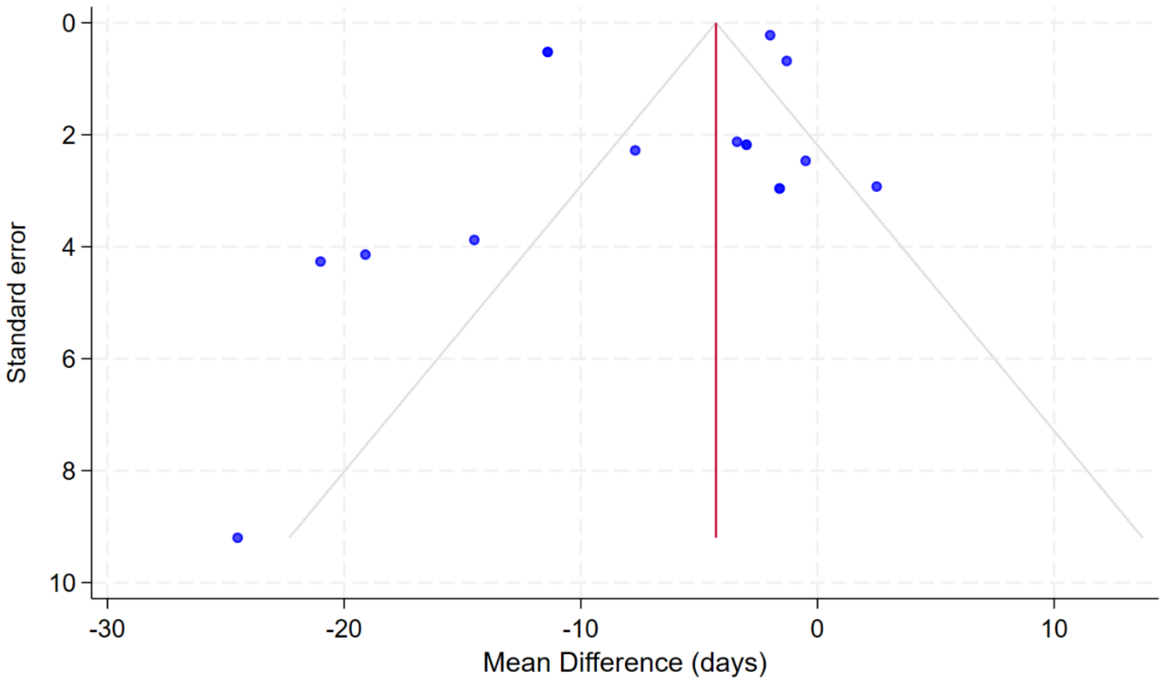

Note. Negative mean difference values favor early tracheostomy (shorter ICU length of stay); positive values favor late tracheostomy.

Supplement Figure S2 Funnel plot of standard error by mean difference for ICU length of stay (evidence from RCTs)

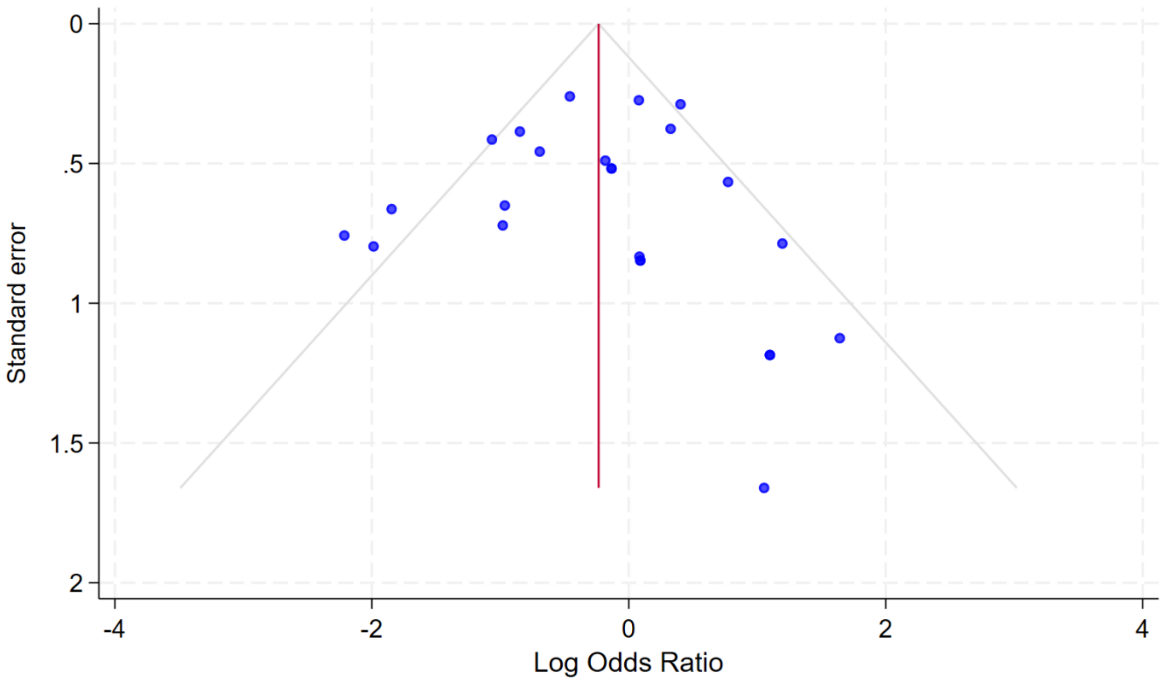

Note. Log odds ratio values less than 0 indicate a lower incidence of ventilator-associated pneumonia in the early tracheostomy group, while values greater than 0 indicate a lower incidence in the late tracheostomy group.

Supplement Figure S3 Funnel plot of standard error by log odds ratio for ventilator-associated pneumonia (evidence from RCTs)

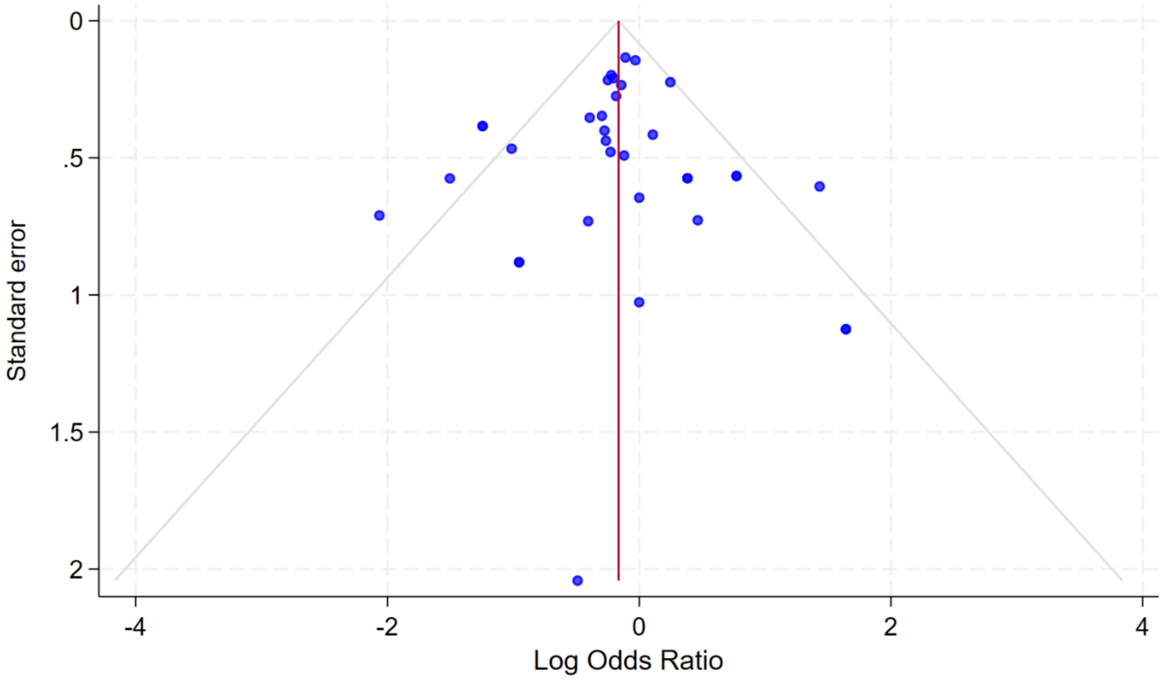

Note. Log odds ratio values less than 0 indicate a lower risk of mortality in the early tracheostomy group (favoring early tracheostomy), while values greater than 0 favor the late tracheostomy group.

Supplement Figure S4 Funnel plot of standard error by log odds ratio for mortality (evidence from RCTs)

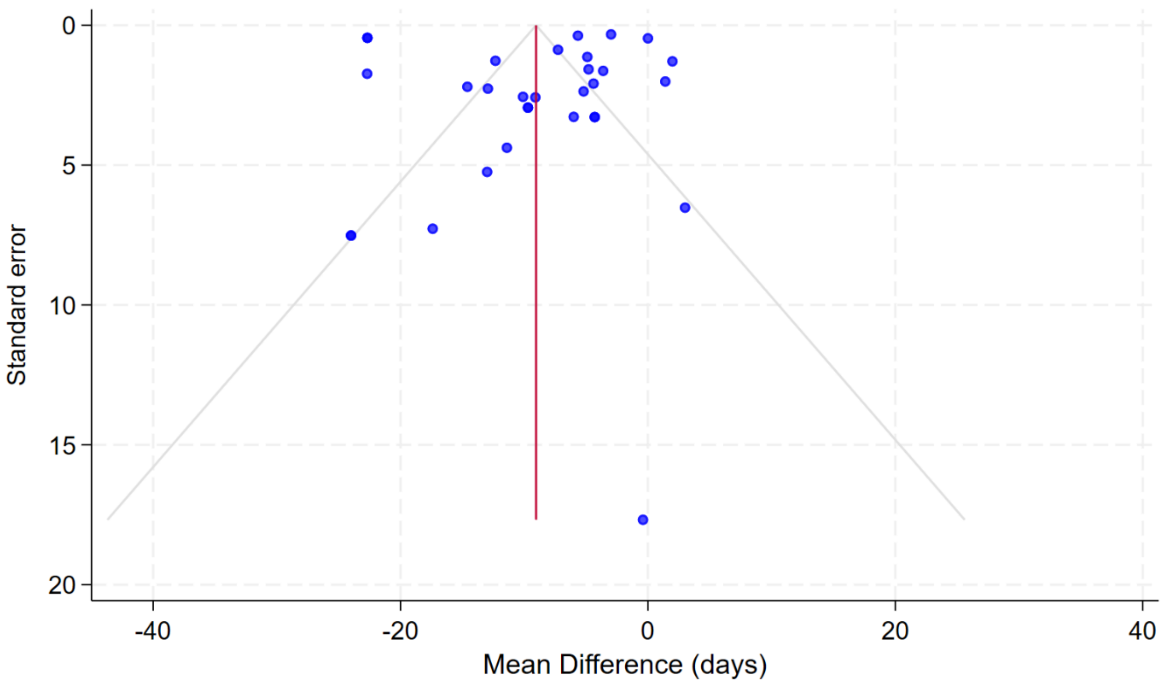

Note. Negative mean difference values indicate a shorter duration of mechanical ventilation in the early tracheostomy group, while positive values favor the late tracheostomy group.

Supplement Figure S5 Funnel plot of standard error by mean difference for mechanical ventilation duration (evidence from non-RCTs)

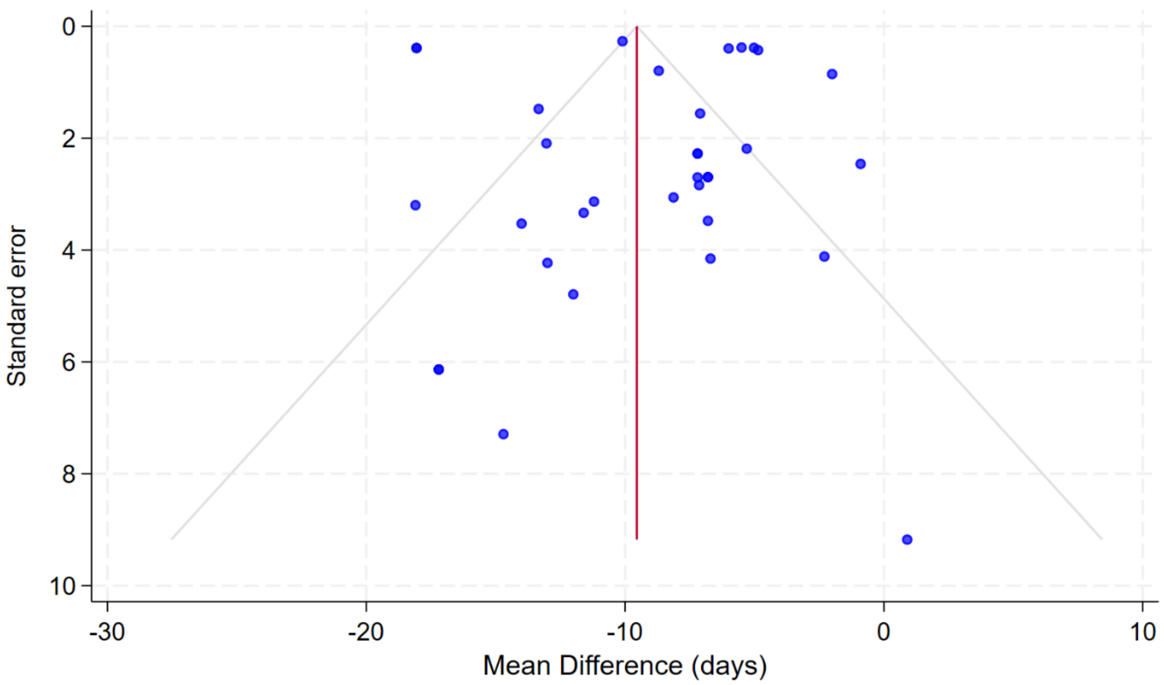

Note. Negative mean difference values indicate a shorter ICU length of stay for the early tracheostomy group, while positive values indicate a shorter stay for the late tracheostomy group.

Supplement Figure S6 Funnel plot of standard error by mean difference for ICU length of stay (evidence from non-RCTs)

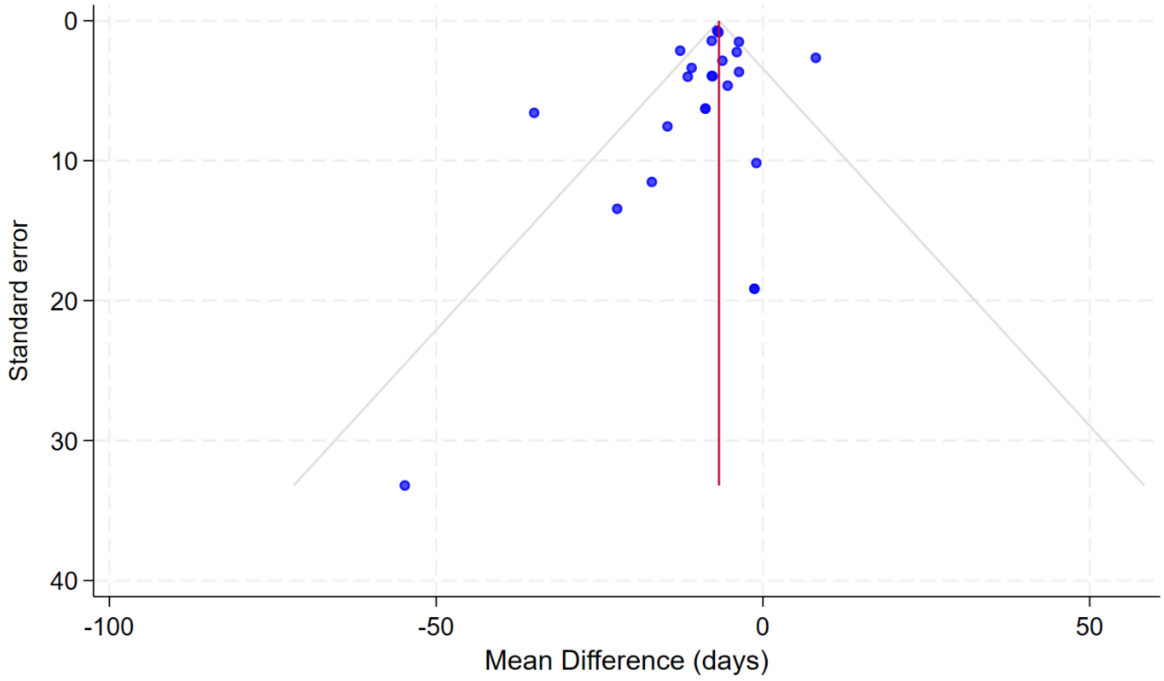

Note. Negative mean difference values indicate a shorter hospital length of stay for the early tracheostomy group, while positive values favor the late tracheostomy group.

Supplement Figure S7 Funnel plot of standard error by mean difference for hospital length of stay (evidence from non-RCTs)

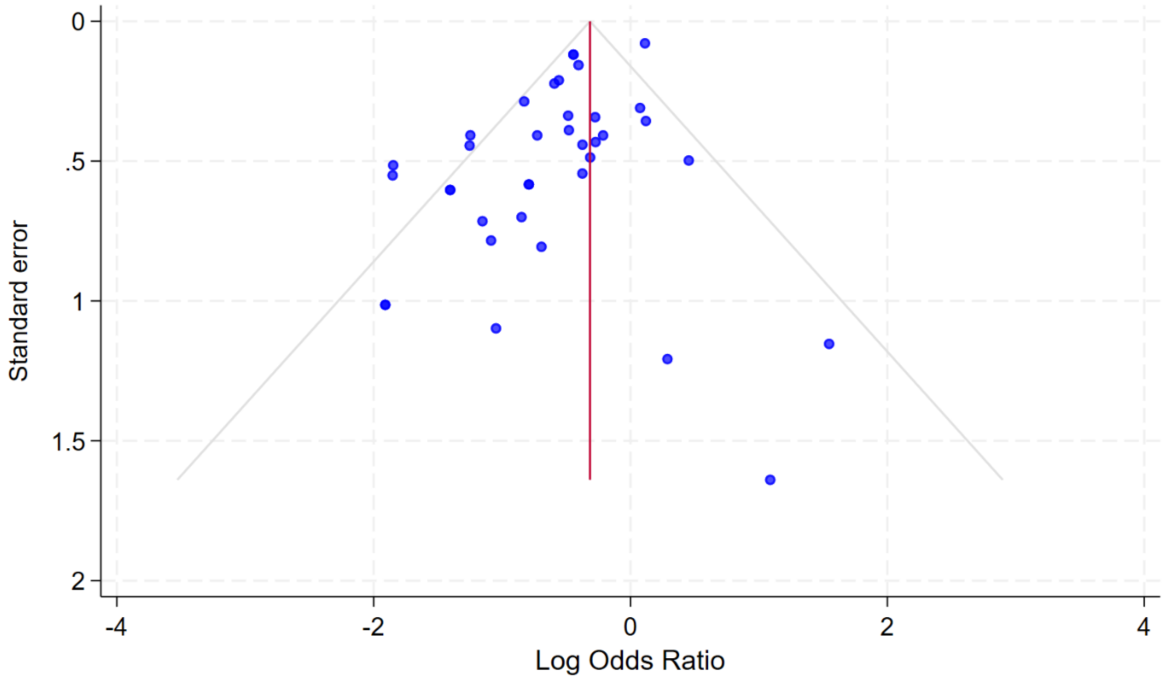

Note. Log odds ratio values less than 0 indicate a lower incidence of ventilator-associated pneumonia in the early tracheostomy group, while values greater than 0 favor the late tracheostomy group.

Supplement Figure S8 Funnel plot of standard error by log odds ratio for ventilator-associated pneumonia (evidence from non-RCTs)

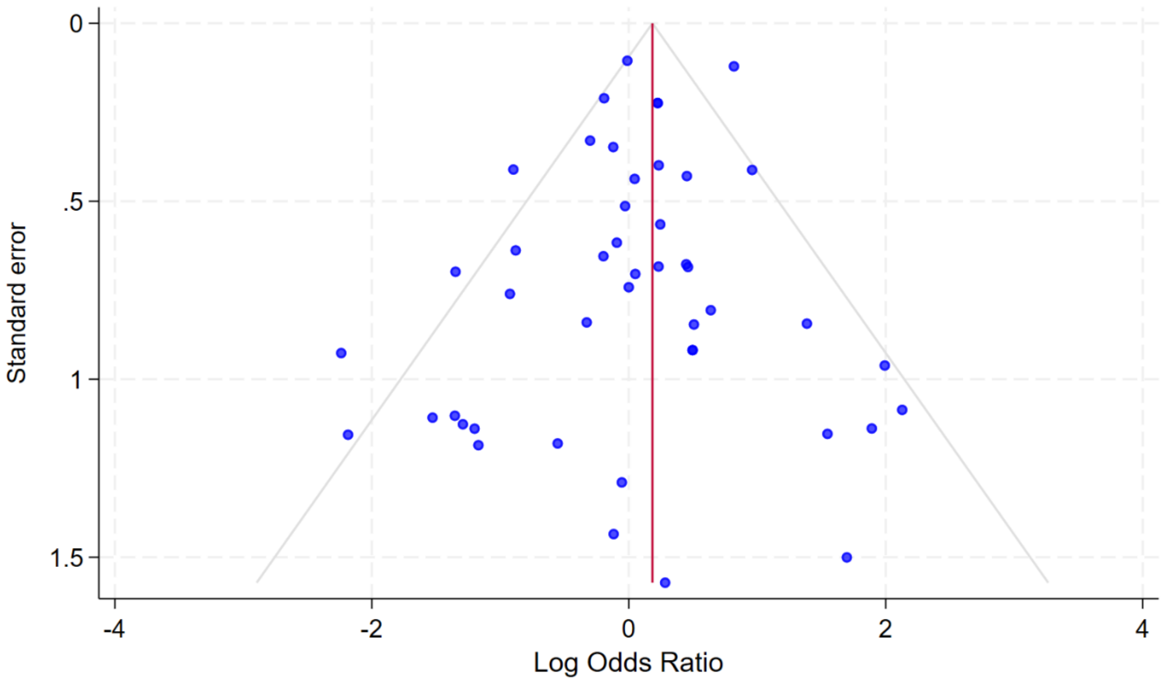

Note. Log odds ratio values less than 0 indicate a lower risk of mortality in the early tracheostomy group (favoring early tracheostomy), while values greater than 0 favor the late tracheostomy group.

Supplement Figure S9 Funnel plot of standard error by log odds ratio for mortality (evidence from non-RCTs)

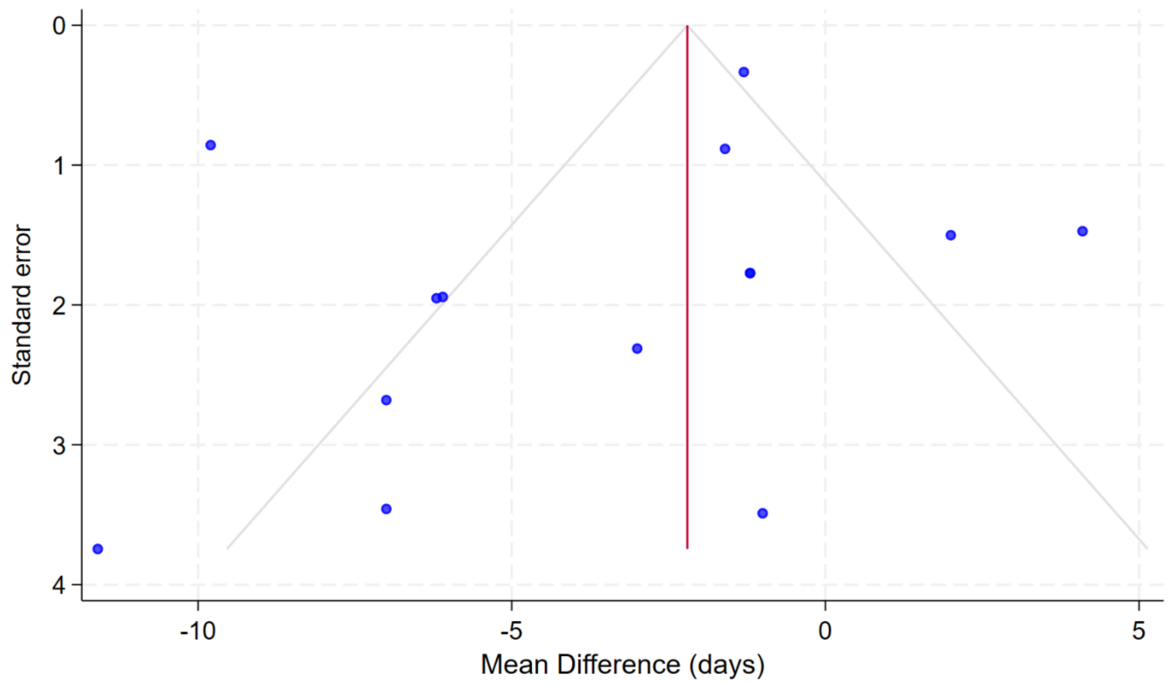

Note. Negative mean difference values indicate a shorter duration of mechanical ventilation in the early tracheostomy group, while positive values favor the late tracheostomy group.

Supplement Figure S10 Funnel plot of standard error by mean difference for mechanical ventilation duration: Subgroup analysis of critically ill patients (evidence from RCTs)

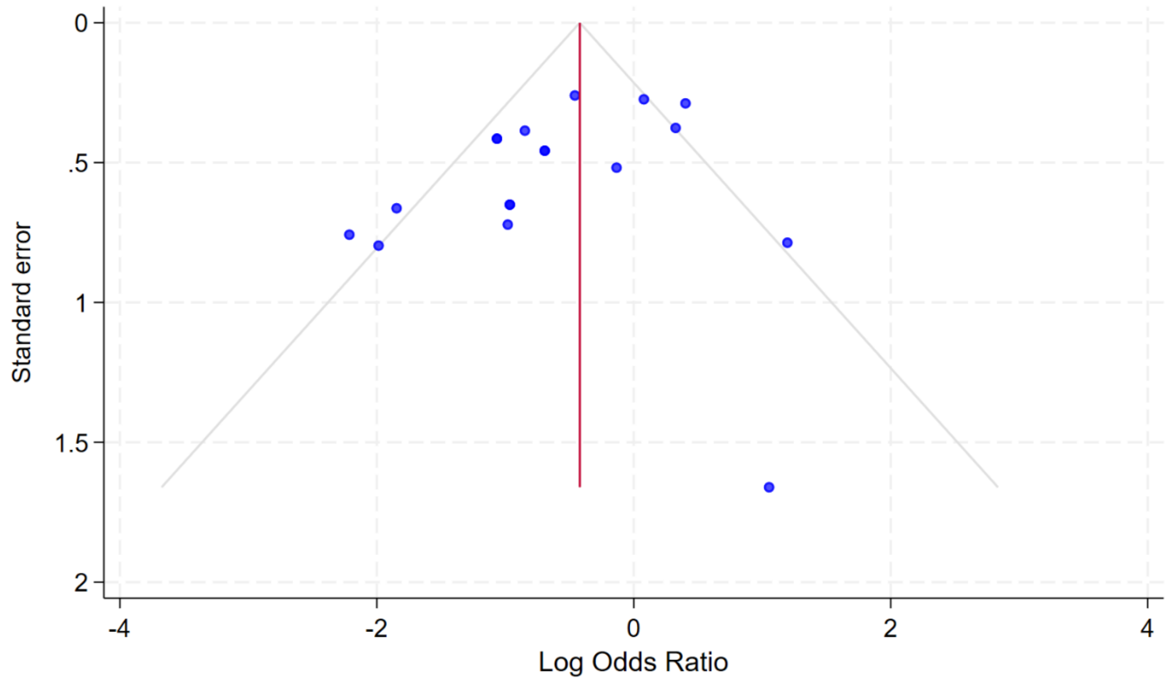

Note. Log odds ratio values less than 0 indicate a lower incidence of ventilator-associated pneumonia in the early tracheostomy group, while values greater than 0 favor the late tracheostomy group.

Supplement Figure S11 Funnel plot of standard error by log odds ratio for ventilator-associated pneumonia: Subgroup analysis of critically ill patients (evidence from RCTs)

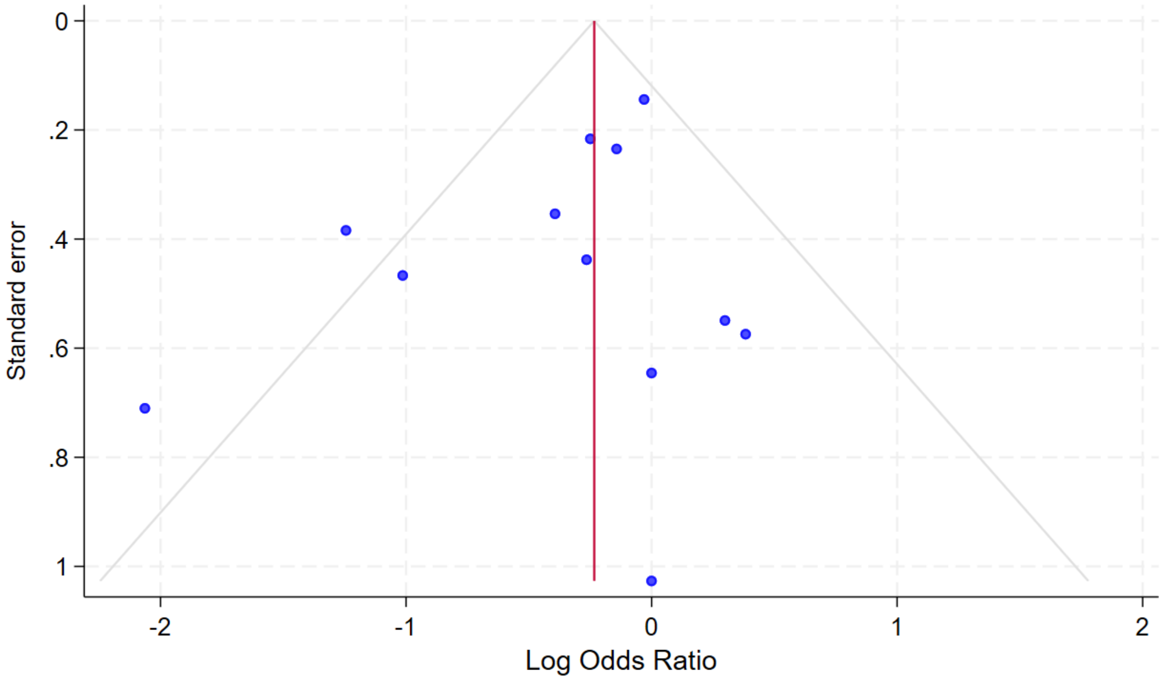

Note. Log odds ratio values less than 0 indicate a lower risk of mortality in the early tracheostomy group (favoring early tracheostomy), while values greater than 0 favor the late tracheostomy group.

Supplement Figure S12 Funnel plot of standard error by log odds ratio for mortality: Subgroup analysis of critically ill patients (evidence from RCTs)

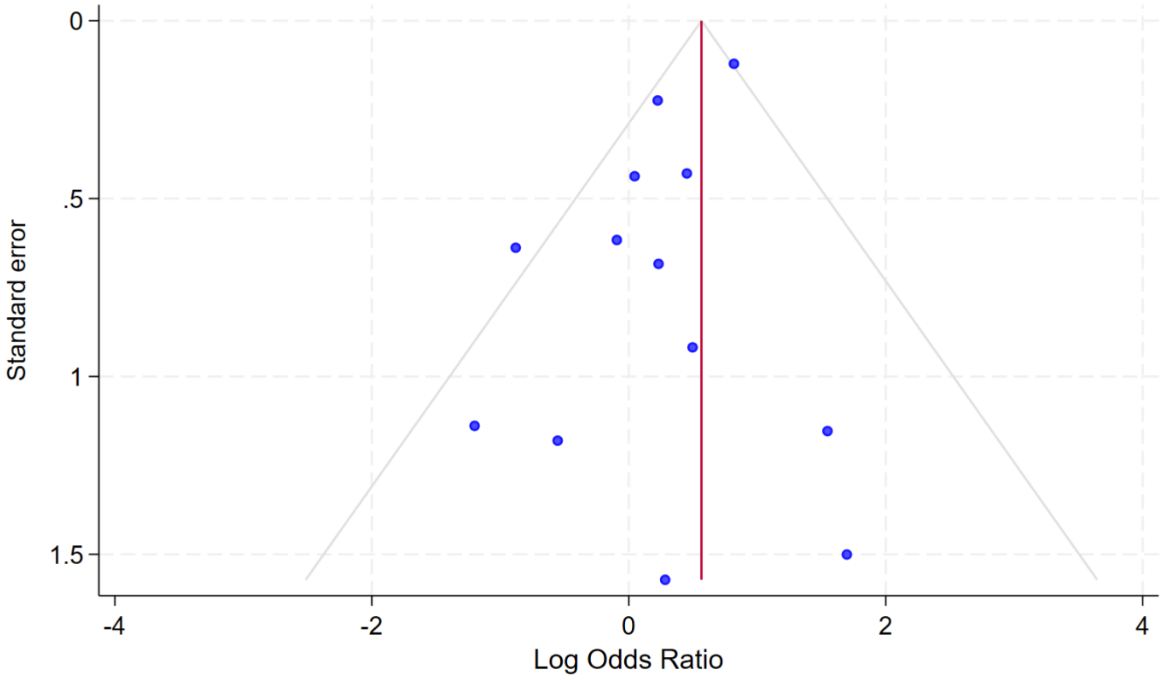

Note. Negative mean difference values indicate a shorter ICU length of stay for the early tracheostomy group, while positive values indicate a shorter stay for the late tracheostomy group.

Supplement Figure S13 Funnel plot of standard error by mean difference for ICU length of stay: Subgroup analysis of traumatic brain injury patients (evidence from non-RCTs)

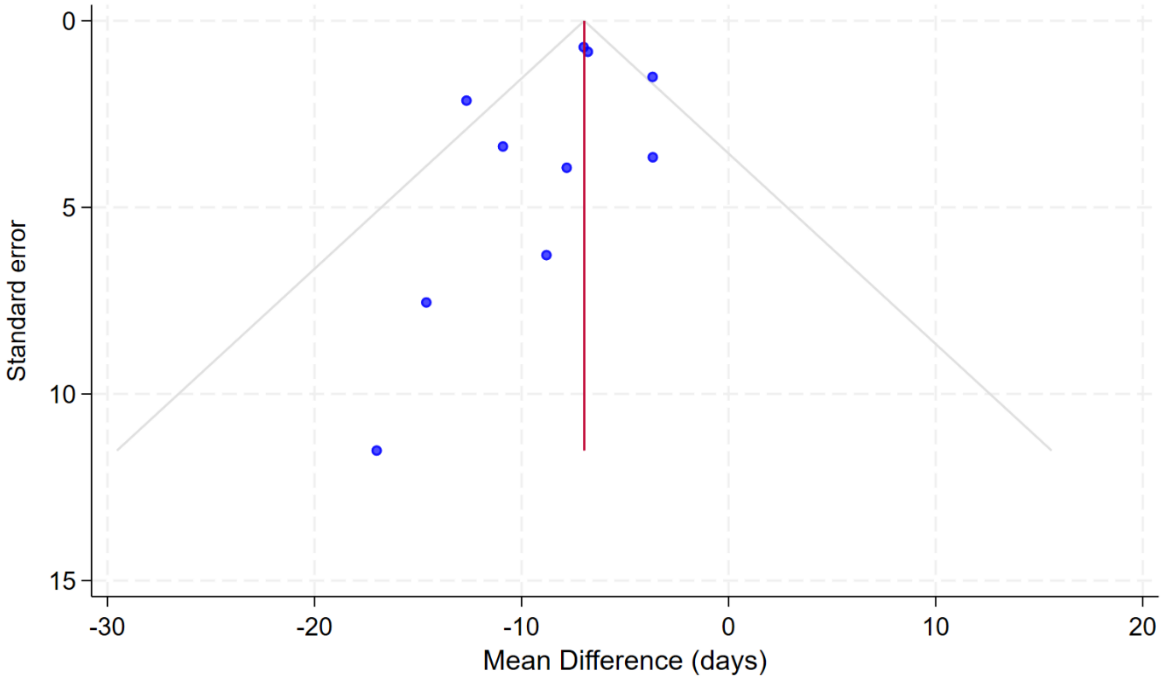

Note. Negative mean difference values indicate a shorter hospital length of stay for the early tracheostomy group, while positive values favor the late tracheostomy group.

Supplement Figure S14 Funnel plot of standard error by mean difference for hospital length of stay: Subgroup analysis of traumatic brain injury patients (evidence from non-RCTs)

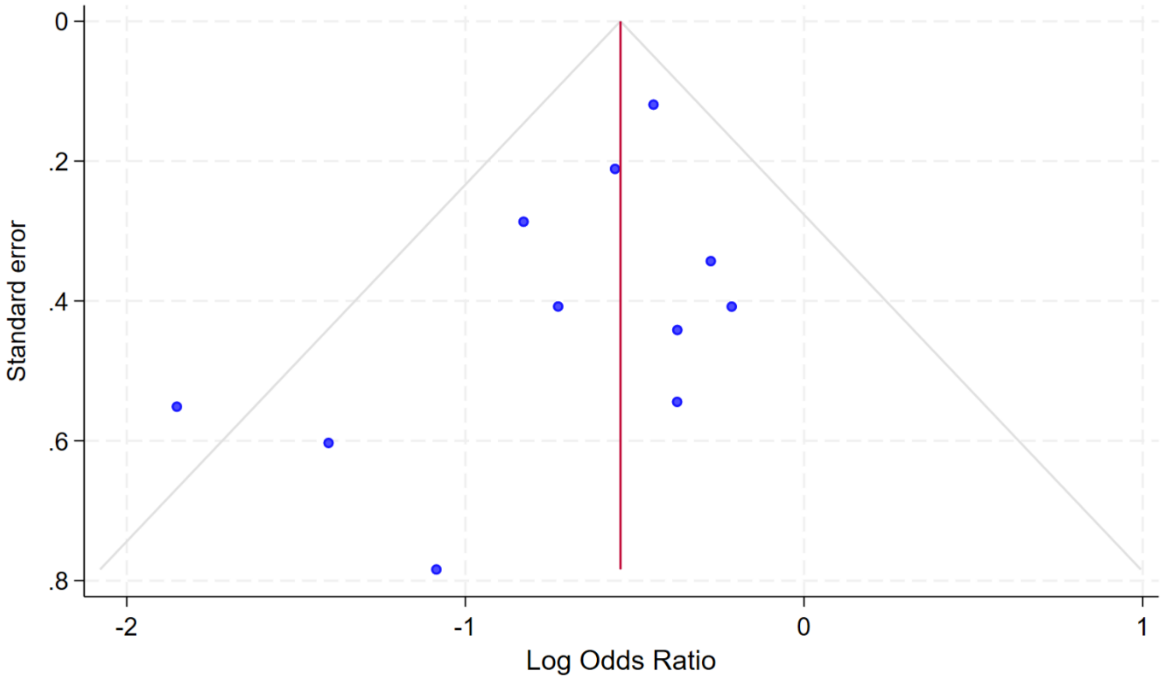

Note. Log odds ratio values less than 0 indicate a lower incidence of ventilator-associated pneumonia in the early tracheostomy group, while values greater than 0 favor the late tracheostomy group.

Supplement Figure S15 Funnel plot of standard error by log odds ratio for ventilator-associated pneumonia: Subgroup analysis of traumatic brain injury patients (evidence from non-RCTs)

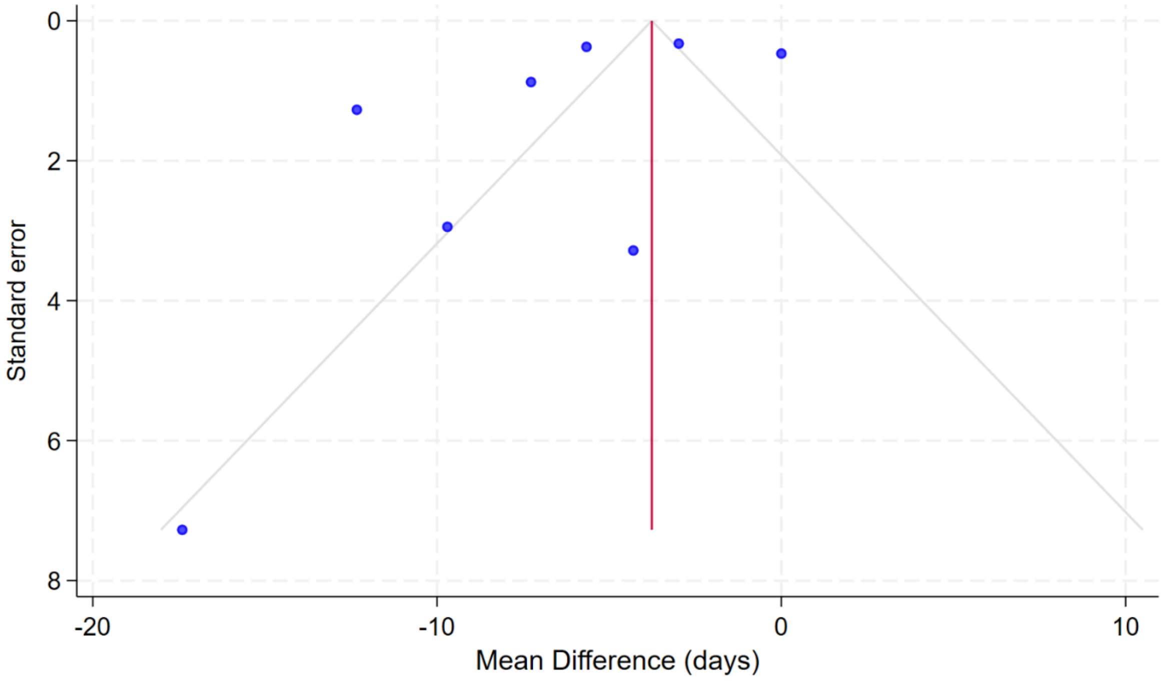

Note. Log odds ratio values less than 0 indicate a lower risk of mortality in the early tracheostomy group (favoring early tracheostomy), while values greater than 0 favor the late tracheostomy group.

Supplement Figure S16 Funnel plot of standard error by log odds ratio for mortality: Subgroup analysis of traumatic brain injury patients (evidence from non-RCTs)

Note. Negative mean difference values indicate a shorter duration of mechanical ventilation in the early tracheostomy group, while positive values favor the late tracheostomy group.

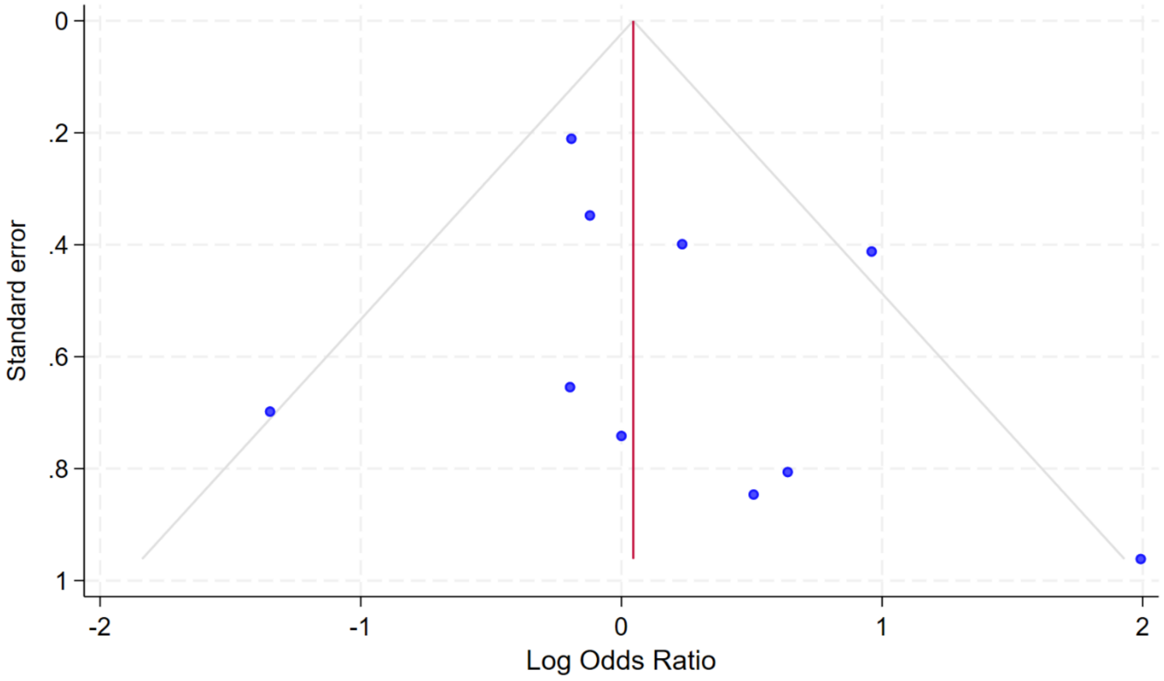

Note. Log odds ratio values less than 0 indicate a lower risk of mortality in the early tracheostomy group (favoring early tracheostomy), while values greater than 0 favor the late tracheostomy group.

Supplement Figure S17 Funnel plot of standard error by log odds ratio for mortality: Subgroup analysis of COVID-19 patients (evidence from non-RCTs)

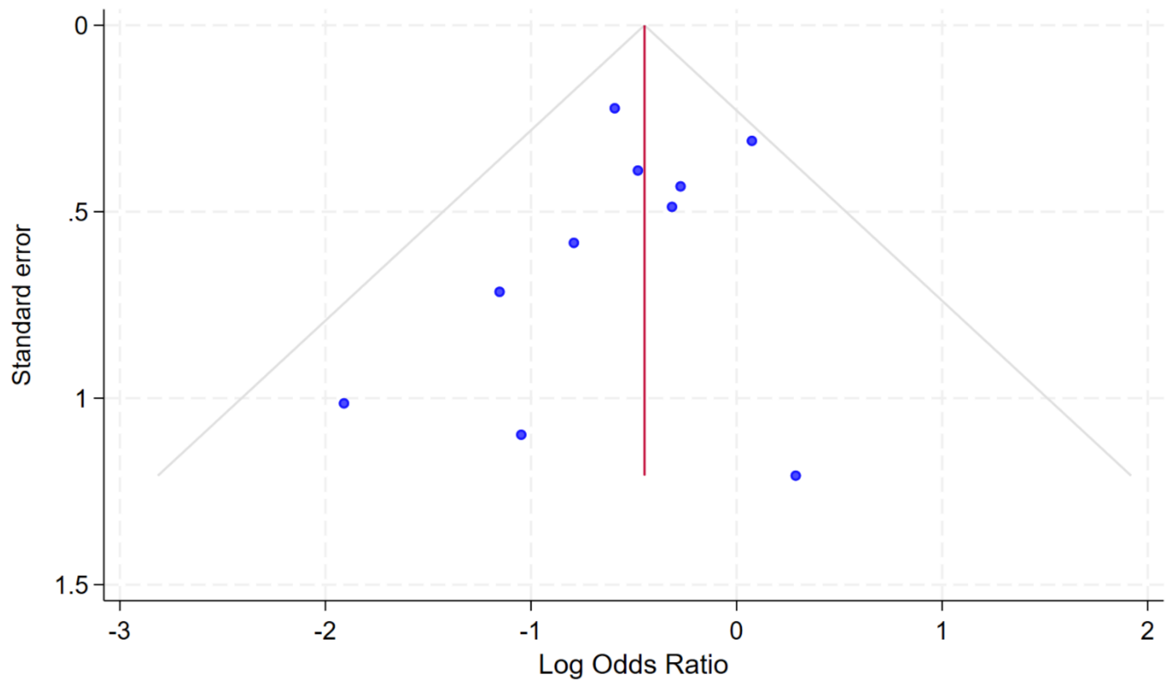

Note. Log odds ratio values less than 0 indicate a lower incidence of ventilator-associated pneumonia in the early tracheostomy group, while values greater than 0 favor the late tracheostomy group.

Supplement Figure S18 Funnel plot of standard error by log odds ratio for ventilator-associated pneumonia: Subgroup analysis of spinal cord injury patients (evidence from non-RCTs)

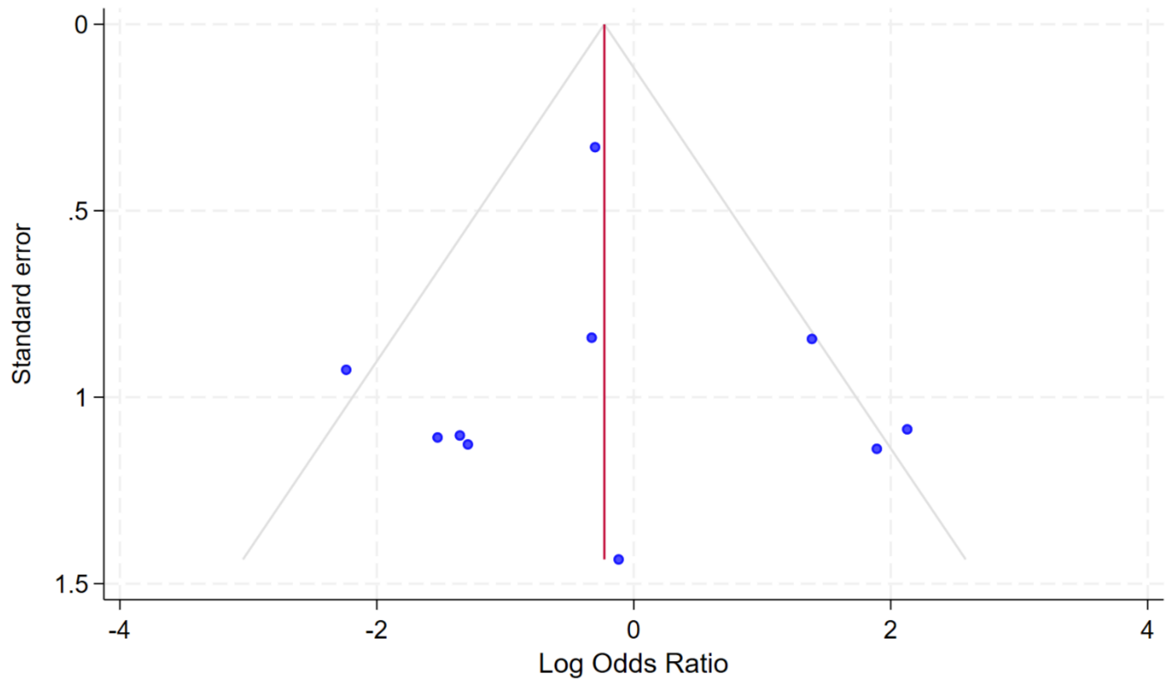

Note. Log odds ratio values less than 0 indicate a lower risk of short-term mortality in the early tracheostomy group (favoring early tracheostomy), while values greater than 0 favor the late tracheostomy group.

Supplement Figure S19 Funnel plot of standard error by log odds ratio for short-term mortality: Subgroup analysis of spinal cord injury patients (evidence from non-RCTs)

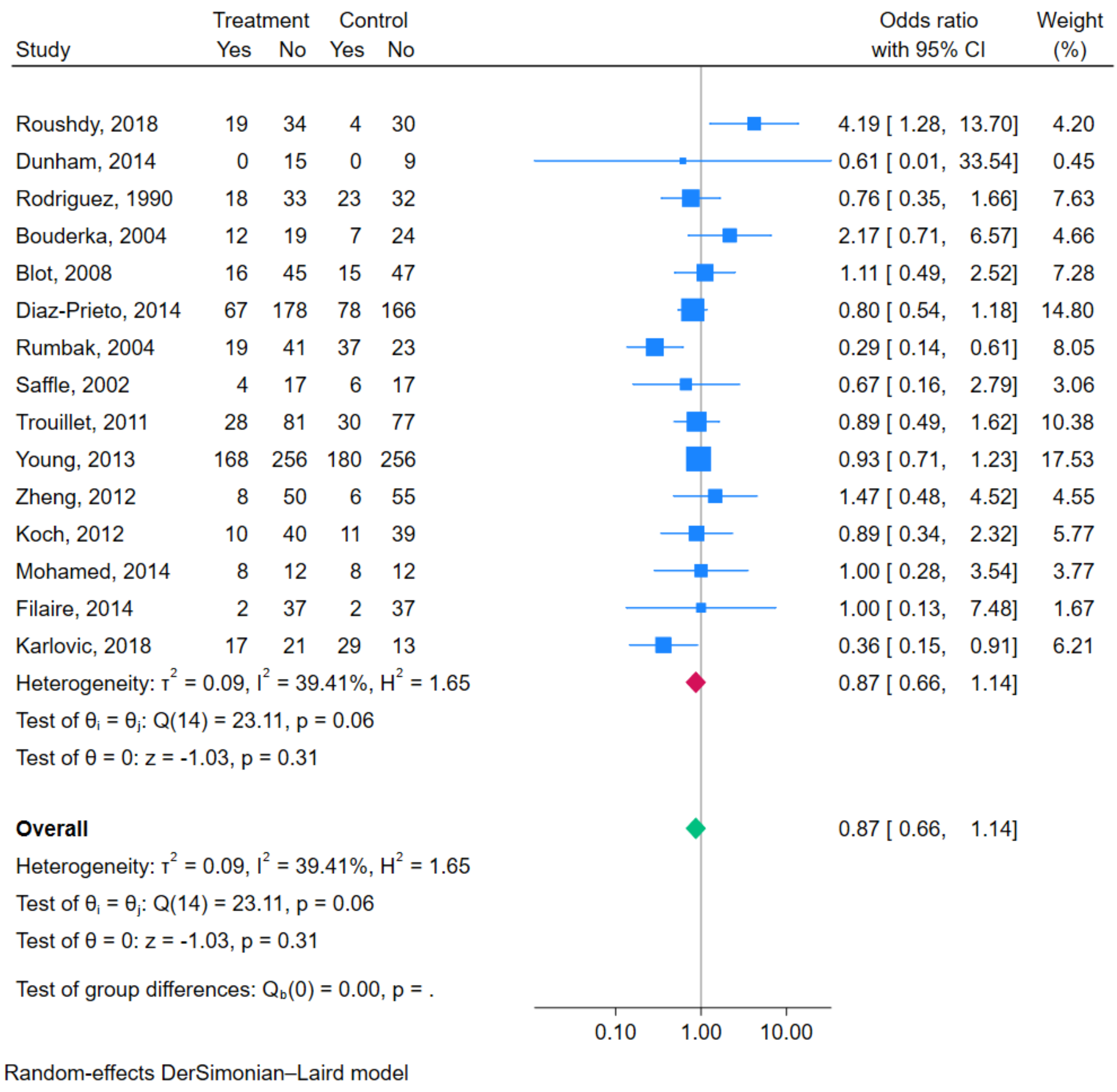

Supplement Figure S20 Forest plot of early versus late tracheostomy for mortality, restricted to RCTs reporting in-hospital mortality and 60-day follow-up.

References

1. Ansems K, Aleksandrova E, Szafran AS, Dahms K, Breuer T, Benstoem C. Early versus late tracheostomy in multiple trauma patients in the intensive care unit: systematic review and meta-analysis. Critical Care. 2023;27(8). doi: 10.1186/s13054-023-04377-x.
2. Arora S, Flower O, Murray NPS, Lee BB. Respiratory care of patients with cervical spinal cord injury: a review. Crit Care Resusc. 2012;14(1):64-73.
3. Baron DM, Hochrieser H, Metnitz PGH, Mauritz W. Tracheostomy is associated with decreased hospital mortality after moderate or severe isolated traumatic brain injury. Wien Klin Wochenschr. 2016;128(11-12):397-403. doi: 10.1007/s00508-016-1004-y. PubMed PMID: WOS:000378779800001.
4. Barquist ES, Amortegui J, Hallal A, Giannotti G, Whinney R, Alzamel H, et al. Tracheostomy in ventilator dependent trauma patients: a prospective, randomized intention-to-treat study. J Trauma. 2006;60(1):91-5. doi: 10.1097/01.ta.0000196743.37261.3f. PubMed PMID: WOS:000235066300021.
5. Bause H, Prause A. Ratings and complications with minimally invasive percutaneous tracheotomy [in German]. Stellenwert und Komplikationen der minimalinvasiven perkutanen Tracheotomieverfahren. 1999;34(10):659-64. doi: 10.1055/s-1999-211.
6. Benito DA, Bestourous DE, Tong JY, Pasick LJ, Sataloff RT. Tracheotomy in COVID-19 patients: a systematic review and meta-analysis of weaning, decannulation, and survival. Otolaryngol Head Neck Surg. 2021;165(3):398-405. doi: 10.1177/0194599820984780.
7. Bhasarkar A, Dolma K. Utility of early tracheostomy in critically ill COVID-19 patients: a systematic review. Indian J Otolaryngol Head Neck Surg. 2023;75(3):1546-56. doi: 10.1007/s12070-022-03280-1. PubMed PMID: WOS:000950046400001.
8. Bittner EA, Schmidt UH. The ventilator liberation process: update on technique, timing, and termination of tracheostomy. Respiratory care. 2012;57(10):1626-34. doi: 10.4187/respcare.01914. PubMed PMID: WOS:000310116200012.
9. Blackwood B, Burns KEA, Cardwell CR, O'Halloran P. Protocolized versus non-protocolized weaning for reducing the duration of mechanical ventilation in critically ill adult patients. Cochrane Database of Systematic Reviews. 2014;2014(11). doi: 10.1002/14651858.CD006904.pub3.
10. Blot F, Similowski T, Trouillet J-L, Chardon P, Korach J-M, Costa M-A, et al. Early tracheotomy versus prolonged endotracheal intubation in unselected severely ill ICU patients. Intensive care medicine. 2008;34(10):1779-87. doi: 10.1007/s00134-008-1195-4.
11. Burns KEA, Adhikari NKJ, Keenan SP, Meade M. Use of non-invasive ventilation to wean critically ill adults off invasive ventilation: meta-analysis and systematic review. BMJ. 2009;338:b1574. doi: 10.1136/bmj.b1574.
12. Burns KEA, Meade MO, Premji A, Adhikari NKJ. Noninvasive ventilation as a weaning strategy for mechanical ventilation in adults with respiratory failure: a Cochrane systematic review. CMAJ. 2014;186(3):E112-22. doi: 10.1503/cmaj.130974.
13. Camacho M, Certal V, Brietzke SE, Holty J-EC, Guilleminault C, Capasso R. Tracheostomy as treatment for adult obstructive sleep apnea: a systematic review and meta-analysis. The Laryngoscope. 2014;124(3):803-11. doi: 10.1002/lary.24433.
14. Cirillo F, Hinkelbein J, Romano GM, Piazza O, Servillo G, De Robertis E. Ventilator associated pneumonia and tracheostomy. Trends in Anaesthesia and Critical Care. 2015;5(6):184-7. doi: 10.1016/j.tacc.2015.10.003.
15. Cox CE, Carson SS. Medical and economic implications of prolonged mechanical ventilation and expedited post-acute care. Seminars in respiratory and critical care medicine. 2012;33(4):357-61. doi: 10.1055/s-0032-1321985.
16. Craven J, Slaughter A, Potter KF. Early tracheostomy: on the cutting edge, some benefit more than others. Current opinion in anaesthesiology. 2022;35(2):236-41. doi: 10.1097/ACO.0000000000001114.
17. Damas P, Frippiat F, Ancion A, Canivet JL, Lambermont B, Layios N, et al. Prevention of ventilator-associated pneumonia and ventilator-associated conditions: a randomized controlled trial with subglottic secretion suctioning. Critical care medicine. 2015;43(1):22-30. doi: 10.1097/CCM.0000000000000674. PubMed PMID: WOS:000346409100025.
18. Damuth E, Mitchell JA, Bartock JL, Roberts BW, Trzeciak S. Long-term survival of critically ill patients treated with prolonged mechanical ventilation: a systematic review and meta-analysis. Lancet Respir Med. 2015;3(7):544-53. doi: 10.1016/S2213-2600(15)00150-2.

19. Delaney A, Bagshaw SM, Nalos M. Percutaneous dilatational tracheostomy versus surgical tracheostomy in critically ill patients: a systematic review and meta-analysis. Crit Care. 2006;10(2):R55. doi: 10.1186/cc4887.
20. Dettmer M, Damuth E, Bartock J, Mitchell J, Trzeciak S. Predictors of mortality in patients with prolonged mechanical ventilation: a systematic review. Critical care medicine. 2015;43(12):180. doi: 10.1097/01.ccm.0000474542.73442.05.
21. Devanand NA, Thiruvenkatarajan V, Liu WM, Sirisinghe I, Court-Kowalski S, Pryor L, et al. Outcomes of percutaneous versus surgical tracheostomy in an Australian quaternary intensive care unit: an entropy-balanced retrospective study. J Intensive Care Soc. 2024;25(3):279-87. Epub 2024/09/03. doi: 10.1177/17511437241238877. PubMed PMID: 39224423; PubMed Central PMCID: PMC11366180.
22. Ding C, Zhang Y, Yang Z, Wang J, Jin A, Wang W, et al. Incidence, temporal trend and factors associated with ventilator-associated pneumonia in mainland China: a systematic review and meta-analysis. BMC infectious diseases. 2017;17(1):468. doi: 10.1186/s12879-017-2566-7.
23. Dunham CM, Cutrona AF, Gruber BS, Calderon JE, Ransom KJ, Flowers LL. Early tracheostomy in severe traumatic brain injury: evidence for decreased mechanical ventilation and increased hospital mortality. Int J Burns Trauma. 2014;4(1):14-24.
24. Filice G, Patel P, Kata P, Kanukuntla A, Patel V, Gallagher N, et al. An overview of outcomes associated with early versus late tracheostomy from a national standpoint. Cureus. 2021;13(7). doi: 10.7759/cureus.16325. PubMed PMID: WOS:000675289600003.
25. Freeman BD, Isabella K, Lin N, Buchman TG. A meta-analysis of prospective trials comparing percutaneous and surgical tracheostomy in critically ill patients. Chest. 2000;118(5):1412-8. doi: 10.1378/chest.118.5.1412.
26. Gadkaree SK, Schwartz D, Gerold K, Kim Y. Use of bronchoscopy in percutaneous dilational tracheostomy. JAMA Otolaryngol Head Neck Surg. 2016;142(2):143-9. doi: 10.1001/jamaoto.2015.3123. PubMed PMID: WOS:000370688300007.
27. Gandía-Martínez F, Martínez-Gil I, Andaluz-Ojeda D, de Lamo FB, Parra-Morais L, Diez-Gutiérrez F. Analysis of early tracheostomy and its impact on development of pneumonia, use of resources and mortality in neurocritically ill patients. Neurocirugia. 2010;21(3):211-21. PubMed PMID: WOS:000279752400003.
28. Garrubba M, Turner T, Grieveson C. Multidisciplinary care for tracheostomy patients: a systematic review. Crit Care. 2009;13(6):R177. doi: 10.1186/cc8159.
29. Gastmeier P, Geffers C. Prevention of ventilator-associated pneumonia: analysis of studies published since 2004. Journal of Hospital Infection. 2007;67(1):1-8. doi: 10.1016/j.jhin.2007.06.011.
30. Ghanima MMA, El Kholy AA, El-Lawaty WM, Zamzam KM, Farhat AA. Comparative study of percutaneous tracheostomy versus standard surgical tracheostomy. Egypt J Chest Dis Tuberc. 2024;73(1):71-7. doi: 10.4103/ecdt.ecdt_47_23. PubMed PMID: WOS:001174354200010.
31. Goligher E, Ferguson ND. Mechanical ventilation: epidemiological insights into current practices. Current opinion in critical care. 2009;15(1):44-51. doi: 10.1097/mcc.0b013e3283220df2.
32. Groves DS, Durbin CG, Jr. Tracheostomy in the critically ill: indications, timing and techniques. Current opinion in critical care. 2007;13(1):90-7. doi: 10.1097/MCC.0b013e328011721e.
33. Gupta S, Dixit S, Choudhry D, Govil D, Mishra RC, Samavedam S, et al. Tracheostomy in adult intensive care unit: an ISCCM expert panel practice recommendations. Indian J Crit Care Med. 2020;24:31-42. doi: 10.5005/jp-journals-10071-G23184.
34. He J, Liu Y, Li L, Yang J, Zhang X, Chen Q, et al. Effect of different durations of prone ventilation on the efficacy of patients with acute respiratory distress syndrome: a small meta-analysis [in Chinese]. Zhonghua Wei Zhong Bing Ji Jiu Yi Xue. 2024;36(5):508-13. doi: 10.3760/cma.j.cn121430-20231108-00958.
35. Herritt B, Chaudhuri D, Thavorn K, Kubelik D, Kyeremanteng K. Early vs. late tracheostomy in intensive care settings: impact on ICU and hospital costs. Journal of critical care. 2018;44:285-8. doi: 10.1016/j.jcrc.2017.11.037.
36. Jerjes W, Hamdoon Z, Abbas S, Algamal Z. Percutaneous dilational tracheostomy and open surgical tracheostomy: systematic review. British Journal of Oral and Maxillofacial Surgery. 2011;49:S69. doi: 10.1016/j.bjoms.2011.03.129.
37. Johnson-Obaseki S, Veljkovic A, Javidnia H. Complication rates of open surgical versus percutaneous tracheostomy in critically ill patients. The Laryngoscope. 2016;126(11):2459-67. doi: 10.1002/lary.26019.
38. Khan ZH, Sasaa MA, Mohammadi M, Alipour A, Hajipour A. Mortality related to intubation in adult general ICUs: a systematic review and meta-analysis. Arch Neurosci. 2020;7(3). doi: 10.5812/ans.89993. PubMed PMID: WOS:000560661000009.

39. Lamb CR, Desai NR, Angel L, Chaddha U, Sachdeva A, Sethi S, et al. Use of tracheostomy during the COVID-19 pandemic: American College of Chest Physicians/American Association for Bronchology and Interventional Pulmonology/Association of Interventional Pulmonology Program Directors expert panel report. Chest. 2020;158(4):1499-514. doi: 10.1016/j.chest.2020.05.571. PubMed PMID: WOS:000579331500056.
40. Lou J, Tian S, Kang X, Lian H, Liu H, Zhang W, et al. Airway management in out-of-hospital cardiac arrest: a systematic review and network meta-analysis. The American journal of emergency medicine. 2023;65:130-8. doi: 10.1016/j.ajem.2022.12.029.
41. Lu Y-h, Qiu X-H, Guo F-m, Yang Y, Qiu H-b. Timing of tracheotomy on the prognosis of patients with prolonged mechanical ventilation: a meta-analysis of randomized controlled trials [in Chinese]. Zhonghua Wai Ke Za Zhi. 2011;49(2):166-71. doi: 10.3760/cma.j.issn.0529-5815.2011.02.015.
42. Mohammadi M, Khamseh AKP, Varpaei HA. Invasive airway "intubation" in COVID-19 patients; statistics, causes, and recommendations: a review article. Anesthesiology and Pain Medicine. 2021;11(3). doi: 10.5812/aapm.115868.
43. Muscedere J, Rewa O, McKechnie K, Jiang X, Laporta D, Heyland DK. Subglottic secretion drainage for the prevention of ventilator-associated pneumonia: a systematic review and meta-analysis. Critical care medicine. 2011;39(8):1985-91. doi: 10.1097/CCM.0b013e318218a4d9.
44. Nkwonta C. Does timing of tracheostomy influence outcomes in the critically ill? Canadian Journal of Respiratory Therapy. 2017;53(3):49.
45. Ramirez P, Ferrer M, Torres A. Prevention measures for ventilator-associated pneumonia: a new focus on the endotracheal tube. Current opinion in infectious diseases. 2007;20(2):190-7. doi: 10.1097/QCO.0b013e328014daac.
46. Roquilly A, Marret E, Abraham E, Asehnoune K. Pneumonia prevention to decrease mortality in intensive care unit: a systematic review and meta-analysis. Clinical infectious diseases : an official publication of the Infectious Diseases Society of America. 2015;60(1):64-75. doi: 10.1093/cid/ciu740.
47. Saffle JR, Morris SE, Edelman L. Early tracheostomy does not improve outcome in burn patients. J Burn Care Rehabil. 2002;23(6):431-8. doi: 10.1097/00004630-200211000-00009. PubMed PMID: WOS:000179239400009.
48. Schreiber AF, Garlasco J, Vieira F, Hui Lau Y, Stavi D, Rigamonti A, et al. Outcomes of weaning from mechanical ventilation after spinal cord injury: a systematic review and meta-analysis. Eur Respir J. 2021;58(suppl 65):PA2434. doi: 10.1183/13993003.congress-2021.PA2434.
49. Sharma A, D Goel A, Bhardwaj P, Kothari N, Goyal S, Kumar D, et al. Tracheostomy outcomes in coronavirus disease 2019: a systematic review and meta-analysis. Anaesthesiology intensive therapy. 2021;53(5):418-28. doi: 10.5114/ait.2021.111594.
50. Shen Y, Cao Q, Zhuo H, Hu M, Chen S. Early versus late tracheostomy in stroke patients: a retrospective analysis. Neuropsychiatric Disease and Treatment. 2022;18:2713-23. doi: 10.2147/NDT.S388062.
51. Szakmany T, Russell P. Impact of early tracheostomy on the ICU patient: Meta-analysis. Critical care medicine. 2013;41(12):A94-A5. doi: 10.1097/01.ccm.0000439539.05170.4c.
52. The Editors Of The Lancet Respiratory M. Retraction and republication-Effect of early versus late or no tracheostomy on mortality of critically ill patients receiving mechanical ventilation: a systematic review and meta-analysis. The Lancet Respiratory medicine. 2015;3(2):102. doi: 10.1016/S2213-2600(15)00005-3.
53. Toeg H, French D, Gilbert S, Rubens F. Incidence of sternal wound infection after tracheostomy in patients undergoing cardiac surgery: a systematic review and meta-analysis. The Journal of thoracic and cardiovascular surgery. 2017;153(6):1394-400.e7. doi: 10.1016/j.jtcvs.2016.11.040.
54. Wu Y, Wang Y, Shen F, Liu B, Qian H, Yang H, et al. Sevoflurane inhalation sedation could shorten the duration of endotracheal intubation and the total length of hospital stay of critical patients after surgery as compared with propofol intravenous sedation: a meta-analysis of 537 patients [in Chinese]. Zhonghua Wei Zhong Bing Ji Jiu Yi Xue. 2019;31(1):44-9. doi: 10.3760/cma.j.issn.2095-4352.2019.01.010.
55. Zheng Y, Sui F, Chen XK, Zhang GC, Wang XW, Zhao S, et al. Early versus late percutaneous dilational tracheostomy in critically ill patients anticipated requiring prolonged mechanical ventilation. Chin Med J. 2012;125(11):1925-30. doi: 10.3760/cma.j.issn.0366-6999.2012.11.016. PubMed PMID: WOS:000305259700017.
56. Araujo de Franca S, Tavares WM, Salinet ASM, Paiva WS, Teixeira MJ. Early tracheostomy in stroke patients: a meta-analysis and comparison with late tracheostomy. Clin Neurol Neurosurg. 2021;203:106554. doi: 10.1016/j.clineuro.2021.106554.

57. Battaglini D, Premraj L, White N, Sutt A-L, Robba C, Cho S-M, et al. Tracheostomy outcomes in critically ill patients with COVID-19: a systematic review, meta-analysis, and meta-regression. Br J Anaesth. 2022;129(5):679-92. doi: 10.1016/j.bja.2022.07.032.
58. Liu CC, Livingstone D, Dixon E, Dort JC. Early versus late tracheostomy: a systematic review and meta-analysis. Otolaryngol Head Neck Surg. 2014;151(1):P39. doi: 10.1177/0194599814541627a34.
59. de Franca SA, Tavares WM, Salinet ASM, Paiva WS, Teixeira MJ. Early tracheostomy in severe traumatic brain injury patients: a meta-analysis and comparison with late tracheostomy. Crit Care Med. 2020;48(4):e325-e31. doi: 10.1097/CCM.0000000000004239.
60. McCredie VA, Alali AS, Scales DC, Adhikari NKJ, Rubenfeld GD, Cuthbertson BH, et al. Effect of early versus late tracheostomy or prolonged intubation in critically ill patients with acute brain injury: a systematic review and meta-analysis. Neurocrit Care. 2017;26(1):14-25. doi: 10.1007/s12028-016-0297-z.
61. Mubashir T, Arif AA, Ernest P, Maroufy V, Chaudhry R, Balogh J, et al. Early versus late tracheostomy in patients with acute traumatic spinal cord injury: a systematic review and meta-analysis. Anesth Analg. 2021;132(2):384-94. doi: 10.1213/ANE.0000000000005212.
62. Foran SJ, Taran S, Singh JM, Kutsogiannis DJ, McCredie V. Timing of tracheostomy in acute traumatic spinal cord injury: a systematic review and meta-analysis. J Trauma Acute Care Surg. 2022;92(1):223-31. doi: 10.1097/TA.0000000000003394.
63. Marra A, Vargas M, Buonanno P, Iacovazzo C, Coviello A, Servillo G. Early vs. late tracheostomy in patients with traumatic brain injury: systematic review and meta-analysis. J Clin Med. 2021;10(15). doi: 10.3390/jcm10153319.
64. Wahlster S, Sharma M, Chu F, Granstein JH, Johnson NJ, Longstreth WT, et al. Outcomes after tracheostomy in patients with severe acute brain injury: a systematic review and meta-analysis. Neurocrit Care. 2021;34(3):956-67. doi: 10.1007/s12028-020-01109-9.
65. Bertini P, Marabotti A, Paternoster G, Sangalli F, Costanzo D, Isirdi A, et al. Early versus late tracheostomy for traumatic brain injury: a systematic review and meta-analysis. Minerva Anestesiol. 2023;89(5):455-67. doi: 10.23736/S0375-9393.23.17176-8. PubMed PMID: WOS:000994260000011.
66. Premraj L, Camarda C, White N, Godoy DA, Cuthbertson BH, Rocco PRM, et al. Tracheostomy timing and outcome in critically ill patients with stroke: a meta-analysis and meta-regression. Crit Care. 2023;27(1):132. doi: 10.1186/s13054-023-04417-6.
67. Selvakumar S, Chan K, Ngatuvai M, Newsome K, Ang D, Bilski T, et al. Timing of tracheostomy in patients with severe traumatic brain injuries: the need for tailored practice management guidelines. Injury. 2022;53(8):2717-24. doi: 10.1016/j.injury.2022.06.031.
68. Qiu Y, Yin Z, Wang Z, Xie M, Chen Z, Wu J, et al. Early versus late tracheostomy in stroke-related patients: a systematic review and meta-analysis. J Clin Neurosci. 2023;114:48-54. doi: 10.1016/j.jocn.2023.06.004.
69. Wang R, Pan C, Wang X, Xu F, Jiang S, Li M. The impact of tracheotomy timing in critically ill patients undergoing mechanical ventilation: a meta-analysis of randomized controlled clinical trials with trial sequential analysis. Heart Lung. 2019;48(1):46-54. doi: 10.1016/j.hrtlng.2018.09.005.
70. Tavares WM, Araujo de Franca S, Paiva WS, Teixeira MJ. Early tracheostomy versus late tracheostomy in severe traumatic brain injury or stroke: a systematic review and meta-analysis. Aust Crit Care. 2023;36(6):1110-6. doi: 10.1016/j.aucc.2022.12.012.
71. Ferro A, Kotecha S, Auzinger G, Yeung E, Fan K. Systematic review and meta-analysis of tracheostomy outcomes in COVID-19 patients. Br J Oral Maxillofac Surg. 2021;59(9):1013-23. doi: 10.1016/j.bjoms.2021.05.011.
72. Adly A, Youssef TA, El-Begermy MM, Younis HM. Timing of tracheostomy in patients with prolonged endotracheal intubation: a systematic review. Eur Arch Otorhinolaryngol. 2018;275(3):679-90. doi: 10.1007/s00405-017-4838-7.
73. Andriolo BN, Andriolo RB, Saconato H, Atallah AN, Valente O. Early versus late tracheostomy for critically ill patients. Cochrane Database Syst Rev. 2015;1:CD007271. doi: 10.1002/14651858.CD007271.pub3.
74. Chong WH, Tan CK. Clinical outcomes of early versus late tracheostomy in coronavirus disease 2019 patients: a systematic review and meta-analysis. J Intensive Care Med. 2022;37(9):1121-32. doi: 10.1177/08850666221098930.
75. Deng H, Fang Q, Chen K, Zhang X. Early versus late tracheotomy in ICU patients: a meta-analysis of randomized controlled trials. Medicine. 2021;100(3):e24329. doi: 10.1097/MD.0000000000024329.
76. Gomes Silva BN, Andriolo RB, Saconato H, Atallah AN, Valente O. Early versus late tracheostomy for critically ill patients. Cochrane Database Syst Rev. 2012(3):CD007271. doi: 10.1002/14651858.CD007271.pub2.

77. Hosokawa K, Nishimura M, Egi M, Vincent J-L. Timing of tracheotomy in ICU patients: a systematic review of randomized controlled trials. Crit Care. 2015;19:424. doi: 10.1186/s13054-015-1138-8.
78. Ji Y, Fang Y, Cheng B, Li L, Fang X. Tracheostomy timing and clinical outcomes in ventilated COVID-19 patients: a systematic review and meta-analysis. Crit Care. 2022;26(1):40. doi: 10.1186/s13054-022-03904-6.
79. Kishihara Y, Yasuda H, Ozawa H, Fukushima F, Kashiura M, Moriya T. Effects of tracheostomy timing in adult patients receiving mechanical ventilation: a systematic review and network meta-analysis. J Crit Care. 2023;77:154299. doi: 10.1016/j.jcrc.2023.154299.
80. Liu X, Wang H-C, Xing Y-W, He Y-L, Zhang Z-F, Wang T. The effect of early and late tracheotomy on outcomes in patients: a systematic review and cumulative meta-analysis. Otolaryngol Head Neck Surg. 2014;151(6):916-22. doi: 10.1177/0194599814552415.
81. Maziak DE, Meade MO, Todd TR. The timing of tracheotomy: a systematic review. Chest. 1998;114(2):605-9. doi: 10.1378/chest.114.2.605.
82. Meng L, Wang C, Li J, Zhang J. Early vs late tracheostomy in critically ill patients: a systematic review and meta-analysis. Clin Respir J. 2016;10(6):684-92. doi: 10.1111/crj.12286.
83. Quinn L, Veenith T, Bion J, Hemming K, Whitehouse T, Lilford R. Bayesian analysis of a systematic review of early versus late tracheostomy in ICU patients. Br J Anaesth. 2022;129(5):693-702. doi: 10.1016/j.bja.2022.08.012.
84. Shan L, Hao P, Xu F, Chen Y-G. Benefits of early tracheotomy: a meta-analysis based on 6 observational studies. Respiratory care. 2013;58(11):1856-62. doi: 10.4187/respcare.02413.
85. Staibano P, Levin M, McHugh T, Gupta M, Sommer DD. Association of tracheostomy with outcomes in patients with COVID-19 and SARS-CoV-2 transmission among health care professionals: a systematic review and meta-analysis. JAMA Otolaryngol Head Neck Surg. 2021;147(7):646-55. doi: 10.1001/jamaoto.2021.0930.
86. Szafran A, Dahms K, Ansems K, Skoetz N, Monsef I, Breuer T, et al. Early versus late tracheostomy in critically ill COVID-19 patients. Cochrane Database Syst Rev. 2023;11:CD015532. doi: 10.1002/14651858.CD015532.
87. Szakmany T, Russell P, Wilkes AR, Hall JE. Effect of early tracheostomy on resource utilization and clinical outcomes in critically ill patients: meta-analysis of randomized controlled trials. Br J Anaesth. 2015;114(3):396-405. doi: 10.1093/bja/aeu440.
88. Cai S-Q, Hu J-W, Liu D, Bai X-J, Xie J, Chen J-J, et al. The influence of tracheostomy timing on outcomes in trauma patients: a meta-analysis. Injury. 2017;48(4):866-73. doi: 10.1016/j.injury.2017.02.023.
89. Chorath K, Hoang A, Rajasekaran K, Moreira A. Association of early vs late tracheostomy placement with pneumonia and ventilator days in critically ill patients: a meta-analysis. JAMA Otolaryngol Head Neck Surg. 2021;147(5):450-9. doi: 10.1001/jamaoto.2021.0025.
90. Dunham CM, Ransom KJ. Assessment of early tracheostomy in trauma patients: a systematic review and meta-analysis. Am Surg. 2006;72(3):276-81. doi: 10.1177/000313480607200316.
91. Griffiths J, Barber VS, Morgan L, Young JD. Systematic review and meta-analysis of studies of the timing of tracheostomy in adult patients undergoing artificial ventilation. BMJ. 2005;330(7502):1243. doi: 10.1136/bmj.38467.485671.E0.
92. Huang HB, Li Y, Ariani F, Chen XL, Lin JD. Timing of tracheostomy in critically ill patients: a meta-analysis. PLoS One. 2014;9(3). doi: 10.1371/journal.pone.0092981. PubMed PMID: WOS:000333675600118.
93. Lu Q, Xie Y, Qi X, Li X, Yang S, Wang Y. Is early tracheostomy better for severe traumatic brain injury? A meta-analysis. World Neurosurg. 2018;112:e324-e30. doi: 10.1016/j.wneu.2018.01.043.
94. Siempos II, Ntaidou TK, Filippidis FT, Choi AMK. Effect of early versus late or no tracheostomy on mortality and pneumonia of critically ill patients receiving mechanical ventilation: a systematic review and meta-analysis. Lancet Respir Med. 2015;3(2):150-8. doi: 10.1016/S2213-2600(15)00007-7.
95. Villemure-Poliquin N, Lessard-Bonaventure P, Costerousse O, Lauzier F, Turgeon AF. Impact of early tracheostomy in non-neurologically injured patients: a systematic review and meta-analysis. Otolaryngol Head Neck Surg. 2020;163(1 SUPPL):P51. doi: 10.1177/0194599820934780.
96. Wang F, Wu Y, Bo L, Lou J, Zhu J, Chen F, et al. The timing of tracheotomy in critically ill patients undergoing mechanical ventilation: a systematic review and meta-analysis of randomized controlled trials. Chest. 2011;140(6):1456-65. doi: 10.1378/chest.11-2024.
