# Supplementary material for: Early Versus Late Tracheostomy: An Umbrella Review and Meta-Analysis
Source: J Clin Med. 2026 Aug 28;15(17):6685. doi: 10.3390/jcm15176685 (PMC13566620; doi:10.3390/jcm15176685)
Supplement: Supplementary file 1 [file jcm-15-06685-s001.zip › jcm-4451223-supplementary/6_File S1.pdf]

File S1

Review title	Impact of Tracheostomy Timing on Clinical Outcomes: An Umbrella Review and Meta-Analysis

Research Question	How does tracheostomy timing influence clinical outcomes and mortality in patients with critical illness, COVID-19, traumatic brain injury, stroke, trauma, and spinal cord injury.

Search strategy developed by
Sven Mostberger, MSc, University of Lucerne; Swiss Paraplegic Research<br>Dr. Gabi Mueller, University of Lucerne; Swiss Paraplegic Research<br>Dr. Marija Glisic, University of Bern; Swiss Paraplegic Research<br>Dr. Magda Gamba, medical subject specialist, ZHB Luzern

Research Question Framework	PICO
1. Population	Tracheostomized patient
2. Intervention	Early tracheostomy
3. Comparison	Late tracheostomy
4. Outcomes	Ventilator-associated pneumonia (VAP)<br>Duration of mechanical ventilation (MV)<br>Intensive care unit/hospital length of stay (LOS)<br>Mortality

Information sources	Last date of search	Records retrieved
Embase.com	09.07.24	2037
Medline (R) (Ovid)	09.07.24	2441
Cochrane Library	10.07.24	151
Web of Science Core Collection	10.07.24	1463
Total before deduplication		6092
**Total after deduplication**		4610

Sentinel papers (PMID & title)	
29255970	Timing of tracheostomy in patients with prolonged endotracheal intubation: a systematic review
36988408	Early versus late tracheostomy for traumatic brain injury: a systematic review and meta-analysis
33546065	Early versus late tracheotomy in ICU patients: A meta-analysis of randomized controlled trials
34508010	Timing of tracheostomy in acute traumatic spinal cord injury: A systematic review and meta-analysis
33009136	Early Versus Late Tracheostomy in Patients With Acute Traumatic Spinal Cord Injury: A Systematic Review and Meta-analysis
36775675	Early tracheostomy versus late tracheostomy in severe traumatic brain injury or stroke: A systematic review and meta-analysis
16553133	Assessment of Early Tracheostomy in Trauma Patients: A Systematic Review and Meta-Analysis
24624310	Early tracheostomy in severe traumatic brain injury: evidence for decreased mechanical ventilation and increased hospital mortality
26635016	Timing of tracheotomy in ICU patients: a systematic review of randomized controlled trials

Embase.com

#1	('respiratory tract intubation'/exp OR 'endotracheal intubation'/exp OR tracheostomy/exp OR (tracheost* OR tracheot* OR ((respiratory OR 'respiratory tract' OR 'respiratory-tract' OR lung OR pulmonar* OR intratracheal* OR intra-tracheal* OR endotracheal* OR orotracheal* OR trachea*) NEAR/3 (intubate* OR tube* OR tubes)) OR ((open OR surgical* OR 'open surgical' OR 'open-surgical') NEAR/3 (tracheost* OR tracheot* OR airway*)) OR ((intubat* OR 'mechanical ventilat*' OR 'mechanical-ventilat*') NEAR/3 (prolong*))):ab,ti,kw)	201,267
#2	('ventilator associated pneumonia'/exp OR 'mechanical ventilation time'/exp OR 'length of stay'/exp OR 'mortality'/exp OR (pneumon* OR VAP OR ((ventilat* OR 'ventilat* associated' OR 'ventilat*-associated' OR 'ventilat* assisted' OR 'ventilat*-assisted' OR 'ventilat* hypersensitivity' OR 'ventilat* acquired' OR 'ventilat*-acquired' OR 'ventilat* induced' OR 'ventilat*-induced' OR 'ventilat* related' OR 'ventilat*-related' OR 'hospital acquired' OR 'hospital-acquired' OR 'healthcare associated' OR 'healthcare-associated' OR 'nosocomial') NEAR/3 (pneumon*)) OR MV OR MVT OR (('mechanical ventilat*' OR 'artificial* ventilat*') NEAR/3 (duration OR time)) OR 'length of stay' OR (('intensive care unit' OR ICU OR hospital*) NEAR/3 ('length of stay' OR day OR days OR stay OR duration)) OR mortality OR survival OR death OR dead OR deceased):ab,ti,kw)	5,089,418
#3	#1 AND #2	51,986
#4	((‘meta analysis (topic)’/exp OR ‘meta analysis’/exp OR (meta NEXT/1 analy*):ab,ti OR metaanaly*:ab,ti OR ‘systematic review (topic)’/exp OR ‘systematic review’/exp OR (systematic NEXT/1 review*):ab,ti OR (systematic NEXT/1 overview*):ab,ti) OR (cancerlit:ab,ti OR cochrane:ab,ti OR embase:ab,ti OR psychlit:ab,ti OR psyclit:ab,ti OR psychinfo:ab,ti OR psycinfo:ab,ti OR cinahl:ab,ti OR cinhal:ab,ti OR ‘science citation index’:ab,ti OR bids:ab,ti) OR ((reference NEXT/1 list*):ab,ti OR bibliograph*:ab,ti OR handsearch*:ab,ti OR (manual NEXT/1 search*):ab,ti OR ‘relevant journals’:ab,ti) OR ((‘data extraction’:ab,ti OR ‘selection criteria’:ab,ti) AND review/it))	889,343
#5	#3 AND #4	2,037
	**TEST WITH SENTINEL ARTICLES**	
#6	'timing of tracheostomy in patients with prolonged endotracheal intubation: a systematic review':ti	1
#7	#5 AND #6	1
#8	'early versus late tracheostomy for traumatic brain injury: a systematic review and meta-analysis':ti	1
#9	#5 AND #8	1
#10	'early versus late tracheotomy in icu patients: a meta-analysis of randomized controlled trials':ti	1
#11	#5 AND #10	1
#12	'early tracheostomy versus late tracheostomy in severe traumatic brain injury or stroke: a systematic review and meta-analysis':ti	1
#13	#5 AND #12	1
#14	'timing of tracheotomy in icu patients: a systematic review of randomized controlled trials':ti	1
#15	#5 AND #14	1
#16	'assessment of early tracheostomy in trauma patients: a systematic review and meta-analysis':ti	1
#17	#5 AND #16	1
#18	'early tracheostomy in severe traumatic brain injury: evidence for decreased mechanical ventilation and increased hospital mortality':ti	1
#19	#5 AND #18	1
#20	'timing of tracheostomy in acute traumatic spinal cord injury: a systematic review and meta-analysis':ti	1
#21	#5 AND #20	1
#22	'early versus late tracheostomy in patients with acute traumatic spinal cord injury: a systematic review and meta-analysis':ti	1
#23	#5 AND #22	1

Medline (R) (Ovid)

#1	exp Tracheostomy/ or respiratory tract intubation/ or endotracheal intubation/ or tracheotomy/	54614
#2	(tracheost* or tracheot* or ((respiratory or respiratory tract or respiratory-tract or lung or pulmonar* or intratracheal* or intra-tracheal* or endotracheal* or orotracheal* or trachea*) adj3 (intubate* or tube* or tubes)) or ((open or surgical* or open surgical or open-surgical) adj3 (tracheost* or tracheot* or airway*)) or ((intubat* or mechanical ventilat* or mechanical-ventilat*) adj3 prolong*)).ti,ab.	79132
#3	1 or 2	113221
#4	exp Pneumonia, Ventilator-Associated/ or exp Healthcare-Associated Pneumonia/ or exp "Length of Stay"/ or exp Mortality/	523611
#5	(pneumon* or VAP or ((ventilat* or ventilat* associated or ventilat*-associated or ventilat* assisted or ventilat*-assisted or ventilat* hypersensitivity or ventilat* acquired or ventilat*-acquired or ventilat* induced or ventilat*-induced or ventilat* related or ventilat*-related or hospital acquired or hospital-acquired or healthcare associated or healthcare-associated or nosocomial) adj3 pneumon*) or MV or MVT or ((mechanical ventilat* or artificial* ventilat*) adj3 (duration or time)) or length of stay or ((intensive care unit or ICU or hospital*) adj3 (length of stay or day or days or stay or duration)) or mortality or survival or death or dead or deceased).ti,ab.	2719987
#6	4 or 5	2874616
#7	3 and 6	19734
#8	meta analysis.mp,pt. or review.pt. or search:.tw.	3391899
#9	7 and 8	2477
#10	9 not (letter or news or comment or editorial or congress).pt. not (exp animals/ not humans/)	2441
	**TEST WITH SENTINEL ARTICLES**	
#11	("29255970" or "36988408" or "33546065" or "34508010" or "33009136").ui.	5
#12	10 and 11	5

Cochrane Library

#1	MeSH descriptor: [Tracheostomy] explode all trees	256
#2	MeSH descriptor: [Intubation, Intratracheal] explode all trees	5763
#3	MeSH descriptor: [Tracheotomy] explode all trees	103
#4	(tracheost* OR tracheot* OR ((respiratory OR respiratory NEXT tract OR respiratory-tract OR lung OR pulmonar* OR intratracheal* OR intra-tracheal* OR endotracheal* OR orotracheal* OR trachea*) NEAR/3 (intubat* OR tube* OR tubes)) OR ((open OR surgical* OR open NEXT surgical OR open-surgical) NEAR/3 (tracheost* OR tracheot* OR airway*)) OR ((intubat* OR mechanical NEXT ventilat* OR mechanical-ventilat*) NEAR/3 prolong*):ti,ab,kw)	23012
#5	#1 OR #2 OR #3 OR #4	23662
#6	MeSH descriptor: [Pneumonia, Ventilator-Associated] explode all trees	630
#7	MeSH descriptor: [Healthcare-Associated Pneumonia] explode all trees	674
#8	MeSH descriptor: [Length of Stay] explode all trees	9572
#9	MeSH descriptor: [Mortality] explode all trees	18908
#10	(pneumon* OR VAP or ((ventilat* OR ventilat* NEXT associated OR ventilator-associated OR ventilat* NEXT assisted OR ventilator-assisted OR ventilat* NEXT hypersensitivity OR ventilat* NEXT acquired OR ventilator-acquired OR ventilat* NEXT induced OR ventilator-induced OR ventilat* NEXT related OR ventilator-related OR hospital NEXT acquired OR hospital-acquired OR healthcare NEXT associated OR healthcare-associated OR nosocomial) NEAR/3 pneumon*):ti,ab,kw)	26350
#11	(MV OR MVT OR ((mechanical NEXT ventilat* OR artificial* NEXT ventilat*) NEAR/3 (duration OR time)):ti,ab,kw)	6613
#12	("length of stay" OR (("intensive care unit" OR ICU OR hospital*) NEAR/3 ("length of stay" OR day OR days OR stay OR duration)):ti,ab,kw)	62175
#13	(mortality OR survival OR death OR dead OR deceased):ti,ab,kw	261603
#14	#10 OR #11 OR #12 OR #13	317281

#15	#5 AND #14 in Cochrane Reviews	151

Web of Science Core Collection

#1	TS=(tracheost* OR tracheot* OR ((respiratory OR "respiratory tract" OR respiratory-tract OR lung OR pulmonar* OR intratracheal* OR intra-tracheal* OR endotracheal* OR orotracheal* OR trachea*) NEAR/3 (intubat* OR tube* OR tubes)) OR ((open OR surgical* OR "open surgical" OR open-surgical) NEAR/3 (tracheost* OR tracheot* OR airway*)) OR ((intubat* OR "mechanical ventilat*" OR mechanical-ventilat*) NEAR/3 prolong*))	97114
#2	TS=(pneumon* OR VAP OR ((ventilat* OR ventilat*associated OR ventilat*-associated OR "ventilat* assisted" OR ventilat*-assisted OR "ventilat* hypersensitivity" OR "ventilat* acquired" OR ventilat*-acquired OR "ventilat* induced" OR ventilat*-induced OR "ventilat* related" OR ventilat*-related OR "hospital acquired" OR hospital-acquired OR "healthcare associated" OR healthcare-associated OR nosocomial) NEAR/3 pneumon*))	295741
#3	TS= (MV OR MVT OR (("mechanical ventilat*" OR "artificial* ventilat*") NEAR/3 (duration OR time)))	179889
#4	TS=("length of stay" OR (("intensive care unit" OR ICU OR hospital*) NEAR/3 ("length of stay" OR day OR days OR stay OR duration))) and Length Of Stay (Search within topic) and Intensive Care Unit (Should – Search within topic) and Length Of Hospital Stay (Should – Search within topic)	85303
#5	TS=(mortality OR survival OR death OR dead OR deceased)	3777550
#6	#5 OR #4 OR #3 OR #2	4204938
#7	#6 AND #1	23304
#8	TS=("systematic review*" OR metaanaly* OR "meta analy*" OR meta-analy* OR synthes*)	3970579
#9	#7 AND #8	1489
#10	TS=(((animal* OR rat OR rats OR mouse OR mice OR rodent OR squirrel OR murine OR nonhuman* OR primate*) NOT (human* OR patient* OR women OR woman OR men OR man)))	3740519
#11	#9 NOT #10	1467
#12	DT=("Conference Abstract" OR Letter OR Note OR Editorial OR Preprint)	4087324
#13	#11 NOT #12	1463
